# Moving beyond the surface: Comparative head and neck myology of threadsnakes (Epictinae, Leptotyphlopidae, Serpentes), with comments on the ‘scolecophidian’ muscular system

**DOI:** 10.1371/journal.pone.0219661

**Published:** 2019-07-18

**Authors:** Angele Martins, Paulo Passos, Roberta Pinto

**Affiliations:** 1 Departamento de Vertebrados, Museu Nacional, Universidade Federal do Rio de Janeiro, Rio de Janeiro, Brazil; 2 Departamento de Ciências Fisiológicas, Instituto de Biociências, Universidade de Brasília, Brasília, Distrito Federal, Brazil; 3 Museu de Arqueologia da Universidade Católica de Pernambuco, Universidade Católica de Pernambuco, Boa Vista, Recife, Pernambuco, Brazil; Laboratoire Arago, FRANCE

## Abstract

Studies on the cephalic myology of snakes provide a series of relevant data on their biology and systematics. Despite the great amount of descriptive studies currently available for the group, much of the knowledge remains obscure for most scolecophidian taxa. This study aimed to describe in detail the cephalic (head and neck) myology of members of the tribe Epictinae, Leptotyphlopidae. We provide the first report of the presence of extrinsic ocular muscles, and a double *Musculus pterygoideus acessorius* in Leptotyphlopidae. A well-developed *M*. *levator anguli oris* is exclusive to the subtribes Renina and Epictina, being reduced in Tetracheilostomina species. Both inter- and intraspecific variations are reported for the head and neck muscles, and such results provide additional data and raise an interesting discussion on the neck-trunk boundaries in snakes. We also provide a discussion on the terminology of a few head muscles in Leptoyphlopidae in comparison to the other lineages of ´Scolecophidia´ (Anomalepididae and Typhlopoidea).

## Introduction

The threadsnakes of the family Leptotyphlopidae currently comprise about 140 recognized species that occur in the sub-Saharan Africa (Leptotyphlopinae and Rhinoleptini) and in the New World (Americas and Antilles), with the subfamily Epictinae containing about 90 species allocated in nine currently recognized genera [1]. Despite the ancient cladogenesis event and the separation between these two main lineages of Leptotyphlopidae [2], all living species present an entirely fossorial lifestyle, actively eating on larvae or adults of social insects [3–5]. Leptotyphlopids fully ingest their prey through a very specialized food intake mechanism named mandibular raking [6], contrasting with the feeding mechanism of the alethinophidian snakes called pterygoidal walk (see [4]). The leptotyphlopid feeding mechanism most likely enables relatively fast food intake compared to alethinophidian snakes, and possibly evolved due to the huge retaliatory response of social insects within their nests [7]. The differences in the leptotyphlopid feeding mechanism and their foraging pattern/diet is visible in the distinct phenotypes of, for example, head muscles and skull and jaw elements [4,7]. Such morphological changes might have played a fundamental role in the diversification of leptotyphlopid lineages and may be interpreted as an adaptation for feeding on social insects, considering the spectacular radiation of ants and termites during the Mesozoic age [8–10]. In this sense, the modification of the muscular-osteological system could represent a key innovation (or even an ecological break) leading potentially to the opening of a new niche for snakes.

Despite the great amount of cephalic myology studies available for snakes (e.g., [11–17]), much of the knowledge remain obscure for several key taxa, with new muscles still being identifyied and described [18]. Some studies on the cephalic myology of snakes provide a series of relevant data regarding their biology (e.g., functional morphology), as well as several systematic issues [19]. Regarding scolecophidians (*sensu lato*, i.e., Anomalepididae + Leptotyphlopidae + Typhlopoidea; *sensu* Vidal et al. [20]), the main difficulties of providing comparative cephalic myology data and hypothesized primary homologies from muscular complexes are caused by the completely different muscular system and innervation patterns as compared to Alethinophidia (e.g., scolecophidian snakes lacking an aponeurotic system in adductor muscles; [21,22,23]). Additionally, the numerous terminologies utilized to describe the cephalic myology of ‘Scolecophidia’ hamper a precise comparative study amongst taxa of this infraorder.

Studies on the post-cephalic muscles in snakes are extremely scarce in comparison to other anatomical complexes such as cephalic myology, cephalic glands, cranial and axial osteology, cartilaginous elements, hemipenes, external morphology, viscera, etc. Besides the classical descriptions of the trunk myology by Mosauer [24] and Gasc [25], very few additional studies are available, which are mostly focused on Alethinophidia (e.g., [26–31]). Descriptive and/or comparative studies of the craniovertebral myology in snakes have received even less attention in the past (e.g., [18,32–36]).

Haas’ seminal studies on the head muscles of both leptotyphlopids and typhlopids [13,14,37] were the first contributions on the head myology of Leptotyphlopidae, and some additional descriptions on the myology of ‘Scolecophidia’ were published later [6,12,21,38,39,40–45]. Regarding the craniovertebral myology of ‘Scolecophidia’, the current available data are those by Jayne [31] and Tsuihiji [33,34], with individuals of Typhlopidae (mostly) and Leptotyphlopidae (only *Rena dulcis*; [34]) included in the samples. However, such studies represent a global approach within Serpentes, and do not provide detailed descriptions for the craniovertebral myology of less inclusive taxa of Leptotyphlopidae.

For that reason, we provide herein a detailed cephalic (head and neck) myology for members of the tribe Epictinae in comparison to other lineages of ‘Scolecophidia’ (*sensu lato*, i.e., Anomalepididae and Typhlopoidea). Additionally, we discuss terminologies and possible functional aspects for this group.

## Materials and methods

We examined 21 specimens from 18 species (S1 Appendix) housed in 15 collections: Coleção Herpetológica da Universidade de Brasília (CHUNB); the Field Museum of Natural History, Chicago, USA (FMNH); Fundación Miguel Lillo (FML); Instituto Butantan (IBSP); the Museum für Naturkunde, Berlin, Germany (ZMB); Natural History Museum of Los Angeles (LACM); Laboratório de Zoologia de Vertebrados, Universidade Federal de Ouro Preto (LZV); the Museum of Comparative Zoology, Cambridge, USA (MCZ); Museu Nacional, Universidade Federal do Rio de Janeiro (MNRJ); Museu de Zoologia da Universidade de São Paulo (MZUSP); Museu Paraense Emílio Goeldi (MPEG); the Natural History Museum, University of Kansas, Lawrence, USA (KU); Oklahoma Museum of Natural History (OMNH); the NMW, San Diego Museum of Natural History (SDMNH); the National Museum of Natural History (USNM); Coleção Zoológica da Universidade Federal do Mato Grosso (UFMT); Illinois Natural History Survey (UIMNH). Each specimen was skinned with the aid of a scalpel and the head was posteriorly immersed in a 2% molecular iodine solution. To describe the origin of craniovertebral muscles, we use “V” + number of the vertebrae, as follows: V1 = atlas, V2 = axis, V3 = first thoracolumbar vertebrae and so on. The photographs were taken with a DFC 450 camera attached to a Leica stereomicroscope M205C and a Zeiss Axiocam (with Axiovision Z-stack software) attached to a Zeiss Discovery V12 stereomicroscope. Schematic drawings based on digital photos were prepared with Inkscape 0.92.

Anatomical terminology follows Haas, Kardong and Cundall [16,21,46] for adductor muscles and Gibson [11] for other head muscles, exept for the intermandibular muscles where we follow Langebartel [43] with the modifications by Groombridge [12] and Cundall [46]. For the craniovertebral myology we follow Tsuihiji [47,48] and Tsuihiji et al. [33,34]. Terminology for the skull description follows Rieppel et al. [49], for the mandible Kley [50], and for the vertebrae Holman [51].

**Fig 1:**
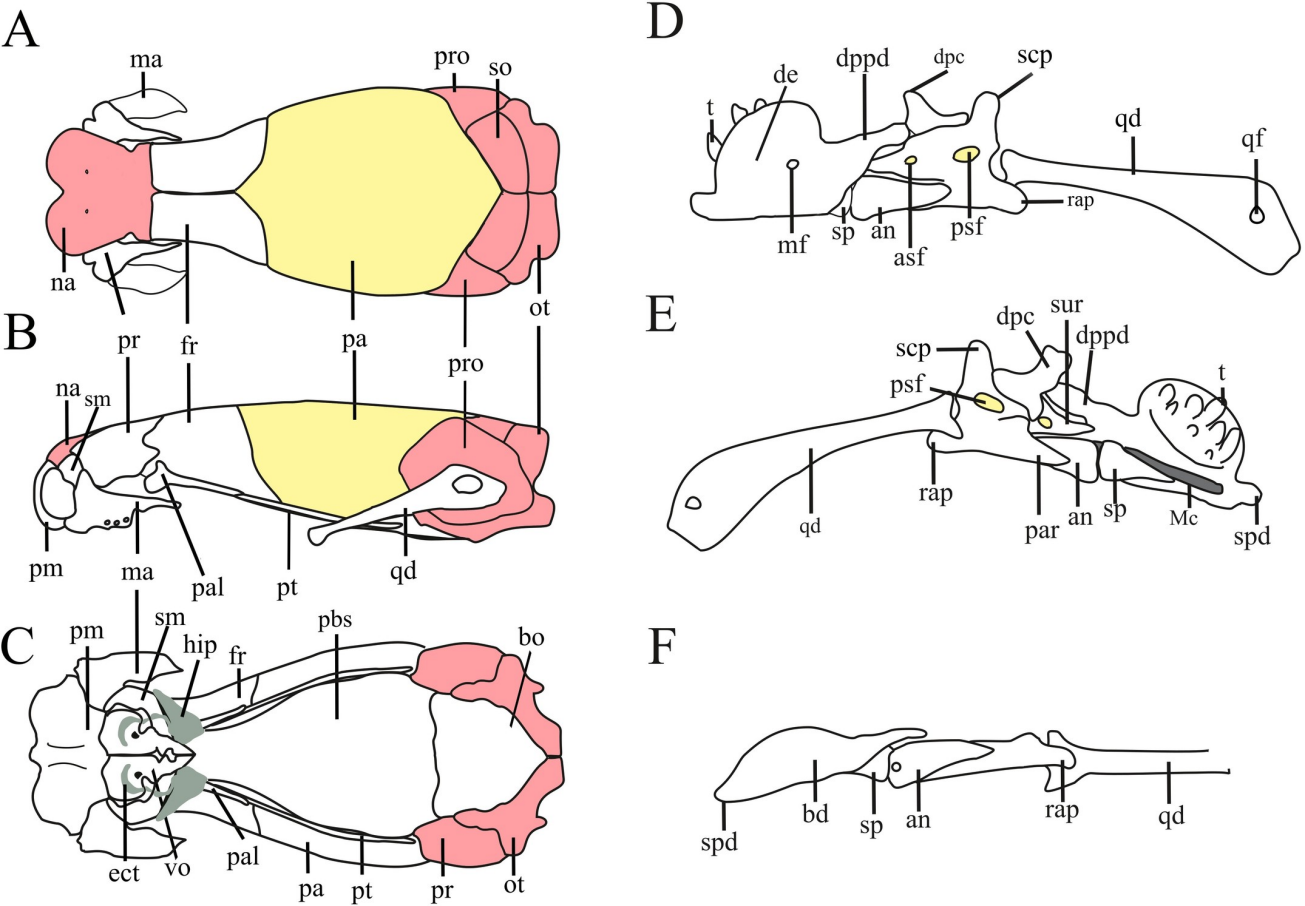
Schematic view of the skull of *Epictia* sp. in dorsal (A), lateral (B), and ventral (C) views, and lower jaw in lateral (D), medial (E), and ventral (F) views as a model for skull general morphology. Red elements present interspecific variation regarding their condition (presence, absence, and fusion of elements). Yellow elements present interspecific variation regarding its level of development amongst Leptotyphlopidae. Abbreviations: an = angular, asf = anterior surangular foramen, bo = basioccipital, dc = dental concha, de = dentary, dpc = dorsal process of coronoid, dppd = dorsoposterior process of dentary, fr = frontal, ma = maxilla, Mc = Meckel’s cartilage, mf = mental foramen, na = nasal, ot = otooccipitals, pa = parietal, pal = palatine, par = prearticular lamina of compound bone, pbs = parabasisphenoid, pm = premaxilla, pr = prefrontal, pro = prootics, psf = posterior surangular foramen, pt = pterygoid, qd = quadrate, qf = quadrate foramen, rap = retroarticular process, scp = supracotylar process of surangular, sm = septomaxilla, so = supraoccipital, spd = symphyseal process of dentary, sur = surangular lamina of compound bone, t = tooth, vo = vomer.

We identified the specimens based on the original descriptions and recent taxonomic studies addressing Epictinae species [52–58]. In many instances, we examined the type material, topotypes, and relevant comparative material in order to refine species’ identifications. The supraspecific taxonomy adopted herein follows Wallach & Boundy [59] and Uetz et al. [1], except for *Rena unguirostris* (*sensu* [2]), which herein is considered as from the genus *Rena* and not *Siagonodon* [59]. A schematic illustration of the skull and lower jaw of *Epictia* sp. is provided in Fig 1 in order to facilitate the identification of muscle origins and insertions. We declare that dissections were performed exclusively in specimens previously deposited in museum collections, thus no approval by ethical committee was required.

## Results

### Head myology

At the beginnig of each description a general overview is given, followed by a genus- or species-specific description if applicable.

#### *Musculus levator anguli oris* (Fig 2; S1 Table)

The muscle originates from the dorsolateral edges of the frontal and parietal bones, or exclusively from the frontal bone, thereby covering the posterior unit of the lateral face of the frontal and the anterior portion of the descending lateral face of the parietal. Fibers converge ventrally, penetrating the ascending portion of the infralabial gland, and insert onto the tip of the dorsoposterior process of the dentary via a rectangular and flat tendon. It extends between the *M*. *adductor mandibulae externus superficialis* and the *M*. *adductor mandibulae externus medialis* portion A, being superficial and laterally covering the posterior portion of the *M*. *adductor externus superficialis*. This muscle is well developed in *Rena*, *Siagonodon* and *Trilepida*, moderately developed in *Epictia*, and reduced in *Tetracheilostoma* and *Mitophis* (see inter- and intraspecific variations in Table 1).

**Fig 2:**
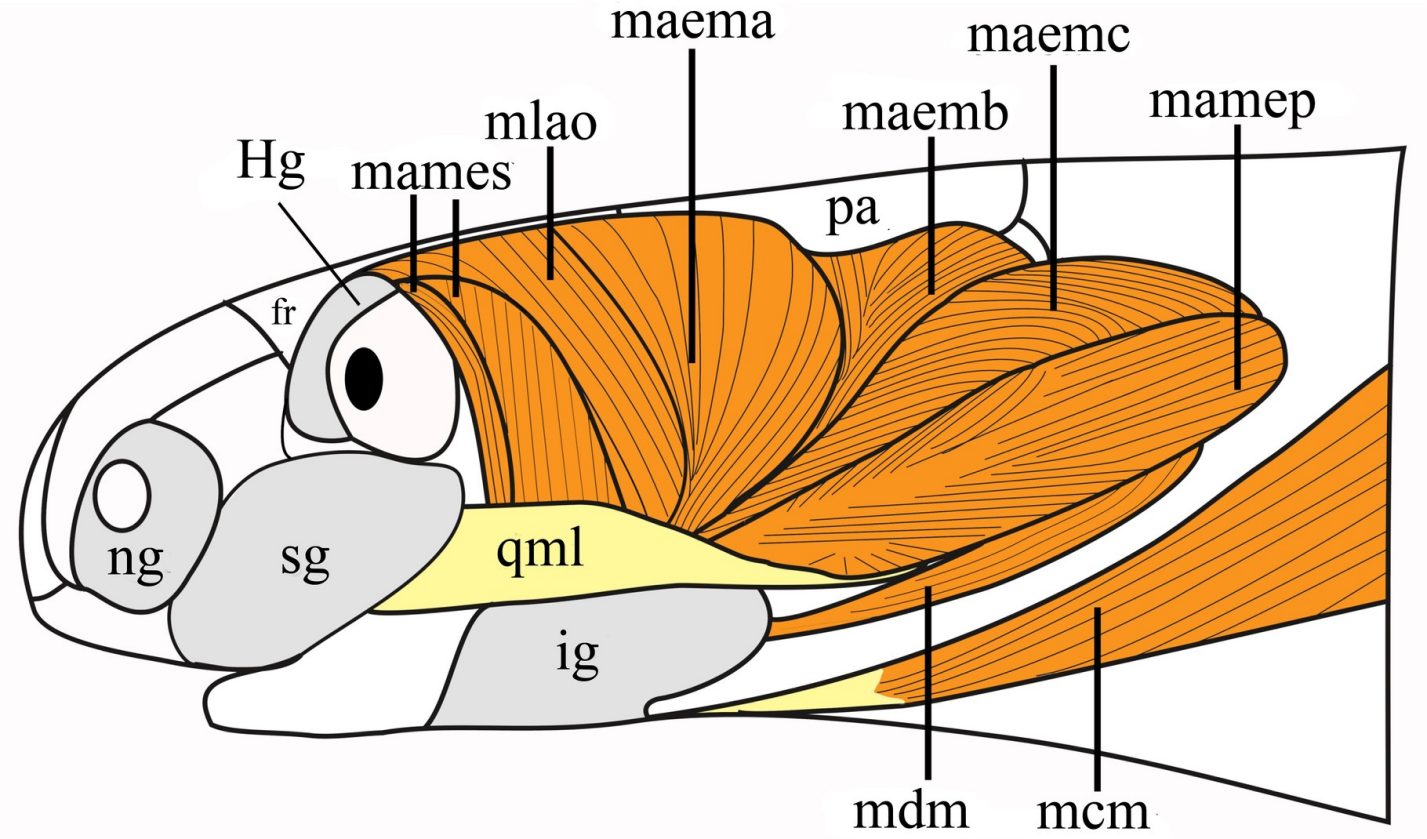
Schematic view of the head muscles in Epictinae; lateral view with no muscle removal. Abbreviations: fr = frontal, Hg = Harderian gland, ig = infralabial gland, maema = *M*. *adductor externus medialis* A, maemb = *M*. *adductor externus medialis* B, maemc = *M*. *adductor externus medialis* C, mamep = *M*. *adductor externus profundus*, mames = *M*. *adductor externus superficialis*, mcm = *M*. *cervicomandibularis*, mdm = *M*. *depressor mandibulae*, mlao = *M*. *levator anguli oris*, na = nasal, ng = nasal gland, pa = parietal, qml = quadratomaxilar ligament, sg = supralabial gland.

**Table 1:** Variability of extrinsic eye muscle presence (P) and absence (A) for members of the subfamily Epictinae. Numbers represent: 1: *M*. *obliquus superior*, 2: *M*. *obliquus inferior*, 3: *M*. *rectus superior*, 4: *M*. *rectus posterior* 5: *M*. *rectus inferior*.

	1	2	3	4	5
*Epictia ater*	A	A	A	A	A
*Epictia phenops*	A	P	P	P	A
*Epictia tenella*	P	P	P	A	A
*Mitophis lepitepileptus*	P	A	P	A	A
*Rena dulcis*	P	P	P	A	A
*Rena humilis*	A	P	A	P	A
*Rena segrega*	A	P	A	A	A
*Rena unguirostris*	A	A	A	A	A
*Siagonodon cupinensis*	P	A	A	A	A
*Tetracheilostoma billineatum*	P	P	A	A	A
*Trilepida brasiliensis*	P	P	A	A	A
*Trilepida dimidiata*	A	A	A	A	A
*Trilepida fuliginosa*	P	A	A	A	A
*Trilepida jani*	A	P	P	A	P
*Trilepida joshuai*	P	A	P	P	A
*Trilepida koppesi*	P	P	P	A	P
*Trilepida macrolepis*	P	P	A	A	A
*Trilepida salgueiroi*	P	P	A	A	A

#### *Musculus adductor mandibulae externus superficialis* (Figs 2 and 3)

This muscle is composed of two portions, with the posterior one being broad and almost completely covered by the *M*. *levator anguli oris*. The anterior portion (when present) is visible anteriorly to the *M*. *levator anguli oris* and posterior to the eye. The anterior portion might be indistinct in some species (see variation below). When present, it originates from the dorsolateral edges of the frontal and prefrontal and, in some specimens, fibers originate from the conjunctive tissue covering the posterior region of the eye. The posterior portion is wide and extends from the posterior region of the frontal bone to the anterior region of the parietal bone; its fibers narrow anteroventrally and insert onto the wide rictal plate. Its ventralmost part and its insertion are covered by the ascending portion of the infralabial gland or the rictal gland (when present; see 7). Variations are described below.

**Fig 3:**
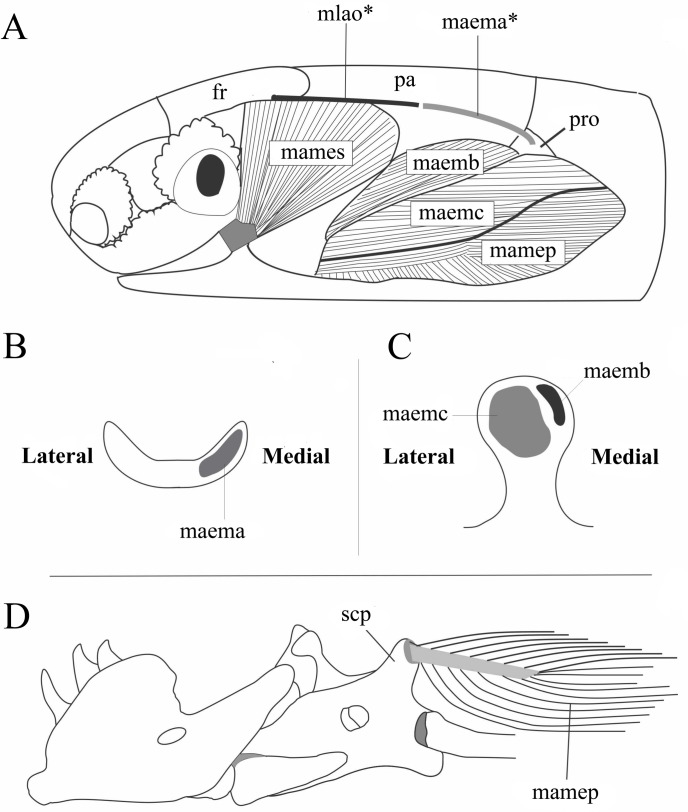
Schematic view of the location and insertion areas of the *M*. *adductor mandibulae externus medialis* portions B and C, *M*. *adductor externus superficialis* and *M*. *aductor externus profundus* in Epictinae. A: Location of the adductor muscles with removal of the *M*. *levator anguli oris* and *M*. *adductor externus medialis* portion A (origins of removed muscles are indicated with gray lines). B: Insertion area of the *M*. *adductor externus medialis* portion A onto the coronoid (dorsal view) portion. C: Insertion area of the *M*. *adductor externus medialis* portions B and C onto the coronoid (posterior view) portion. D: Lateral view of the mandible with illustration of the *M*. *adductor externus profundus* tendon inserting onto the supracotylar process of the compound bone. Abbreviations: fr = frontal, maema = *M*. *adductor externus medialis* A, maemb = *M*. *adductor externus medialis* B, maemc = *M*. *adductor externus medialis* C, mamep = *M*. *adductor externus profundus*, mames = *M*. *adductor externus superficialis*, mlao = *M*. *levator anguli oris*, pa = parietal, pro = prootic, scp = supracotylar process of the compound bone.

**Genus *Epictia*—*Origin*:** The anterior portion is narrow, extending from the anterior part of the posterior half of the frontal. The origin of the posterior portion may extend (1) from the posterolateral region of the prefrontal, the posterior half of the frontal and the conjunctive tissue covering the eye (*Epictia tenella* MCZ 60801) or (2) exclusively from the posterior half of the frontal, immediately posterior to the posterior limit of the prefrontal (*Epictia tenella* MNRJ 16827, *E*. *ater*, *E*. *phenops*). In both conditions, its origin extends posteriorly through the dorsolateral face of the parietal until reaching either only its anterior third (*E*. *tenella* MNRJ 16827), reaching half its length (*E*. *tenella* MCZ 60801, *E*. *ater*), or exceeding half of parietal (*E*. *phenops*). ***Insertion*:** In *E*. *ater* fibers expand laterally in its anterior area, inserting onto the dorsal and dorsolateral region of the rictal plate, as well as onto the fascia that covers the infralabial gland adjacent to the rictal plate. Its posterior portion inserts onto the medial region of the rictal plate, slightly ventrally to the insertion of its anterior region. In *E*. *tenella*, its anterior portion inserts exclusively onto the lateral region of the rictal plate. Its posterior portion inserts both onto the medial face of the rictal plate, as well as onto the posterolateral region of the rictal plate. This character could not be observed in *E*. *phenops* because of damage in that region.

***Mitophis lepitepileptus*—*Origin*:** Its anterior portion originates from the anterior half of the frontals, bending anteroventrally towards the lateral region of the wide rictal plate. The origin of its posterior portion is distinct from its anterior one by the subtriangular exposed surface of the Harderian gland. The posterior portion originates from the last third of the posterolateral region of the frontal, extending to the end of the first half of the parietal. ***Insertion*:** Fibers of the anterior and posterior portions are indistinct ventrally inserting onto the dorsal and lateral faces of the rictal plate.

**Genus *Rena*—*Origin*:** The origin of the anterior portion is narrow, from the anterolateral region of the frontal (*R*. *humilis*), or from the posterior region of the frontal (*R*. *dulcis*, *R*. *segrega*, *R*. *unguirostris*), and might include a short region of the conjunctive tissue covering the eye posteriorly (*R*. *dulcis*, 50%, *n* = 1; *R*. *segrega*), or even a posterolateral region of the prefrontal (*R*. *dulcis*, 50%; *n* = 1). The origin of the posterior portion is from the posterior half of the frontal (*R*. *dulcis*, *R*. *segrega*, *R*. *unguirostris*) or its anterior half (*R*. *humilis*); or immediately after the posterior limit of the prefrontal, extending posteriorly covering half of the parietal (*R*. *dulcis*, *R*. *segrega*, *R*. *humilis*). Alternatively, the origin can be narrow, not even covering half of the parietal (*R*. *unguirostris*). ***Insertion*:** Its anterior fibers extend ventrally and insert onto the dorsal and dorsolateral region of the supralabial region of the rictal plate (*R*. *dulcis*). The muscle might have an anteroventral portion at its anterior part, but no portions can be distinguished at the origin (*R*. *dulcis*). Additionally, the posteriormost fibers of the anterior portion might insert onto the anterodorsal portion of the infralabial gland (*R*. *dulcis*, *R*. *humilis*, *R*. *segrega*). Its posterior portion inserts laterally (*R*. *dulcis*, *R*. *segrega*) or medially (*R*. *humilis*) onto the rictal plate, and posteroventrally onto the infralabial surface of the rictal plate. Some posterior and medial fibers might insert onto the dorsal portion of the infralabial gland (*R*. *humilis*, *R*. *segrega*), or a thick bundle of fibers might attach to the middorsal portion of that gland by a discrete tendinous plate (*R*. *segrega*). In *R*. *humilis*, the fibers of the anterior and posterior portions are indistinct ventrally and insert onto the posterior region of the rictal plate.

***Siagonodon cupinensis*—*Origin*:** The anterior and posterior portions of the *M*. *adductor mandibulae externus superficialis* are indistinct. The muscle originates from the posterior limit of the prefrontal and extends posteriorly over the posterior region of the frontal, to reach half of the parietal. ***Insertion*:** The anterior half descends ventrally to insert onto the dorsal portion of the infralabial gland, while the posterior half descends anteroventrally, covering the posterior half of the eye. The posterior half also expands ventrally to insert onto the posterior edge of the rictal plate.

***Tetracheilostoma bilineatum*—*Origin*:** Its anterior portion originates from the posterior half of the frontal, while its posterior portion, almost completely exposed, extends from the posterior half of the frontal to the posterior 1/3 of the parietal. ***Insertion*:** Its anterior portion inserts dorsally and laterally onto the supralabial face of the rictal plate, while its posterior portion inserts medially onto the rictal plate.

**Genus *Trilepida*—*Origin*:** The origin of the anterior portion is from the posterior region of the first third of the frontal (*Tr*. *salgueiroi*), from the posterior region of the second third of the frontal (*Tr*. *brasiliensis*, *Tr*. *dimidiata*, *Tr*. *koppesi*), along the posterior half (*Tr*. *joshuai*) or posterior end (*Tr*. *macrolepis*) of the frontal, and might even cover the posterolateral region of the prefrontal (*Tr*. *brasiliensis*). The posterior portion originates from the posterior half of the frontal (*Tr*. *brasiliensis*, *Tr*. *dimidiata*, *Tr*. *koppesi*, *Tr*. *salgueiroi*), or its last third (*Tr*. *koppesi*, *Tr*. *macrolepis*), extending posteriorly from the laterodorsal face of half the parietal (*Tr*. *brasiliensis*, *Tr*. *joshuai*, *Tr*. *koppesi*. *Tr*. *macrolepis*, *Tr*. *salgueiroi*), or even reaching its posterior limit, bending ventrally (*Tr*. *dimidiata*). In *Tr*. *fuliginosa* and *Tr*. *jani*, the anterior and posterior portions are indistinct, originating from the posterior half of the frontal, extending posteriorly to reach half of the parietal. ***Insertion*:** Its anterior portion extends ventrally to insert onto the dorsal and laterodorsal regions of the supralabial surface of the rictal plate (*Tr*. *brasiliensis*, *Tr*. *dimidiata*, *Tr*. *joshuai*, *Tr*. *koppesi*). Its posterior portion inserts onto the medial surface of the rictal plate (*Tr*. *brasiliensis*, *Tr*. *dimidiata*, *Tr*. *joshuai*, *Tr*. *koppesi*). In species where the anterior portion is distinct, the fibers descend and insert onto the lateral and medial face of the rictal plate (*Tr*. *fuliginosa*, *Tr*. *jani*). The insertion of the anterior portion in *Tr*. *macrolepis* was damaged in the course of the dissection, while the posterior portion inserts onto the medial surface of the infralabial region of the rictal plate, and also medially onto the dorsal portion of the infralabial gland.

#### *Musculus adductor mandibulae externus medialis* (Figs 2 and 3)

This muscle is composed of three dintinguishable portions of the *M*. *adductor mandibulae externus medialis*: *M*. *adductor mandibulae externus medialis* portion A (MAEMA), *M*. *adductor mandibulae externus medialis* portion B (MAEMB) and *M*. *adductor mandibulae externus medialis* portion C (MAEMC). Located on the lateral and temporal areas of the head, it covers the lateral region of the parietal (MAEMA) and prootic (MAEMB and MAEMC). The MAEMA represents the anteriormost portion of the medial external adductors, covering a posterior region of the *M*. *adductor externus superficialis*. The MAEMB is almost completely covered by the MAEMC, the latter representing the stoutest portion of the medial external adductors, being located dorsally to the *M*. *adductor externus profundus*. MAEMA originates from the dorsolateral surface of the parietal, posterior to the branch of the trigeminal nerve that emerges on the dorsolateral surface of the head, posteriorly and continuous to the posterior limit of the *M*. *levator anguli oris*. MAEMB originates from the posterior extremity of the lateral edge of the parietal and might extend to the prootic (see variation below). Fibers are anteriorly oriented and can be completely or partially covered by the MAEMA throughout its length. MAEMC represents a stouter portion of the external medial muscles, covering most of the lateral surface of the mandible (quadrate and compound bone = preaticular lamina+surangular lamina+retroarticular process). MAEMC originates from the posterior surface of the articular portion of the quadrate, with many fibers originating from the dorsal region of the cartilage associated with the proximal head of the quadrate, as well as including a smaller region of the prootics and along the dorsal and medial surface of the quadrate. MAEMA inserts onto a restricted area, which is exclusively on the dorsomedial face of the coronoid. MAEMB inserts onto a small area of the dorsomedial region of the posterior face of the coronoid. MAEMC inserts onto a wide region of the coronoid, extending over almost its complete posterior surface (except areas where MAEMB already inserted). Variations are described below.

**Genus *Epictia—Origin*:** The origin of MAEMA extends from the frontoparietal suture posteriorly to half of the parietal. MAEMB originates from the posterolateral area of the parietal but does not reach the prootic posteriorly. MAEMC originates from the proximal epiphysis, dorsal and medial face of the quadrate, as well as from the lateral face of the prootic. ***Insertion*:** MAEMA inserts onto a narrow area of the dorsal face of the coronoid. MAEMB insertion is also narrow, in a vertical and medial area of the posterior face of the coronoid, while MAEMC inserts onto all the posterior surface of the coronoid.

***Mitophis lepitepileptus—Origin*:** The origin of MAEMA lies more anteriorly due to the short extension of the *M*. *levator anguli oris*. MAEMA originates anterior and adjacent to the frontoparietal suture, extending posteriorly to an anterior region of the parietal. MAEMB originates exclusively from the posterolateral portion of the parietal, while MAEMC originates from the cartilage associated with the proximal epiphysis of the quadrate and throughout its dorsal, dorsolateral and medial face. ***Insertion*:** MAEMA insertion was damaged during dissection of the specimen. MAEMB insertion is wide, from all the dorsoposterior surface of the coronoid, while MAEMC insertion is narrow and limited to a more lateroventral area of the coronoid posterior face.

**Genus *Rena—Origin*:** MAEMA originates from the posterior half of the parietal (*R*. *dulcis*, *R*. *humilis* SDSNH 34302, *R*. *segrega*) or from the frontoparietal suture (*R*. *humilis* SDSNH 33950). Its origin can also extend posteriorly almost reaching the parietal-prootic suture in lateral view (*R*. *dulcis*), half the parietal (*R*. *humilis*), or a small anterior area of the prootics (*R*. *segrega*). MAEMB originates from the lateral region of the lateral surface of the parietal, as well as the anterolateral region of the prootic (*R*. *dulcis*, *R*. *humilis*, *R*. *segrega*, *R*. *unguirostris*). MAEMC originates from the cartilage associated with the proximal epiphysis of the quadrate (*R*. *dulcis*, *R*, *humilis*, *R*. *unguirostirs*) and from its dorsal, dorsolateral and medial face (*R*. *dulcis*, *R*. *segrega*). In *R*. *unguirostris*, MAEMA is indistinct or absent. ***Insertion*:** MAEMA inserts onto a small medial area of the coronoid’s dorsal face (*R*. *dulcis*) or only onto a restricted area at the level of the coronoid’s dorsal face (*R*. *humilis*, *R*. *regrega*). MAEMB has a small circular insertion onto the medial area of the posterior face of the coronoid (*R*. *dulcis*, *R*. *humilis*, *R segrega*) or wide throughout the dorsoposterior area of the coronoid’s posterior face (*R*. *unguirostris*). MAEMC inserts onto all the posterior face of the coronoid, including its dorsal area (*R*. *dulcis*, *R*. *humilis*) or it is restricted to a more dorsal area of this element (*R*. *unguirostris*).

***Siagonodon cupinensis—Origin*:** MAEMA is indistinct or absent. MAEMB originates from the posterolateral region of the parietal, as well as the anterolateral area of the prootic. MAEMC originates from the cartilage of the proximal epiphysis of the quadrate, from the quadrate’s dorsal region, or from its dorsolateral and medial face. ***Insertion*:** MAEMB inserts onto a small vertical area of the coronoid’s posteromedial face. MAEMC inserts onto a wide circular region of the dorsoposterior face of the coronoid.

***Tetracheilostoma bilineatum—Origin*:** MAEMA originates from an anterior area of the posterior half of the parietal. MAEMB has a wide origin, extending from lateral at the posterior region of the parietal to the anterior region of the prootics. MAEMC originates exclusively from the cartilage of the proximal epiphysis of the quadrate. ***Insertion*:** MAEMA inserts dorsally onto the medial region of the coronoid. MAEMB inserts onto a narrow region middorsally on the coronoid’s posterior face. MAEMC inserts onto a wide circular area restricted posteriorly to the dorsal and dorsolateral region of the coronoid.

**Genus *Trilepida—Origin*:** MAEMA originates from the posterior region of the frontal, ventrally to the *M*. *levator anguli oris* (*Tr*. *brasiliensis*); from the posterior region of the first quarter of the frontal (*Tr*. *dimidiata*, *Tr*. *koppesi*) or from half the parietal (*Tr*. *fuliginosa*, *Tr*. *jani*, *Tr*. *joshuai*, *Tr*. *macrolepis*, *Tr*. *salgueiroi*). Its origin extends posteriorly reaching the posterior region of the parietal (*Tr*. *jani*, *Tr*. *dimidiata*, *Tr*. *koppesi*, *Tr*. *macrolepis*) or reaching a small anterior region of the prootics (*Tr*. *brasiliensis*, *Tr*. *fuliginosa*, *Tr*. *joshuai*, *Tr*. *salgueiroi*). MAEMB originates from the posterolateral face of the parietal and from an anterolateral region of the prootics (*Tr*. *brasiliensis*, *Tr*. *fuliginosa*, *Tr*. *jani*, *Tr*. *dimidiata*, *Tr*. *joshuai*, *Tr*. *koppesi*), or from a vertical area in the parietal-prootics suture (*Tr*. *salgueiroi*). MAEMC originates exclusively from the cartilage associated to the proximal epiphysis of the quadrate (*Tr*. *brasiliensis*, *Tr*. *joshuai*, *Tr*. *koppesi*). In *Tr*. *macrolepis*, MAEMC is bifid with fibers originating from a central tendon that attaches to the cartilage of the quadrate’s proximal epiphysis. ***Insertion*:** MAEMA inserts throughout all the dorsal face of the coronoid (*Tr*. *brasiliensis*, *Tr*. *fuliginosa*, *Tr*. *jani*, *Tr*. *dimidiata*, *Tr*. *joshuai*, *Tr*. *Koppesi*, *Tr*. *macrolepis*) or only onto the most medial half of its dorsal face (*Tr*. *salgueiroi*). In all species analyzed, the insertion of MAEMB onto the posterior face of the coronoid is narrow. The fibers are vertical in the most medial region as well as the dorsalmost region of the posterior face of the coronoid. MAEMC inserts onto the dorsal, dorsolateral or medial face of the quadrate (*Tr*. *brasiliensis*, *Tr*. *jani*, *Tr*. *salgueiroi*). In *Tr*. *fuliginosa*, the insertion onto the dorsal and dorsomedial face of the quadrate extends exclusively along its posterior half.

#### *Musculus adductor mandibulae externus profundus* (Figs 2 and 3; S1 Table)

The *M*. *adductor mandibulae externus profundus* (MAMEP) is a wide bipennate muscle that extends to the medially located *Musculus adductor mandibulae externus medialis* muscles. In lateral view, MAMEP occupies most of the posterodorsal skull region. This muscle covers the lateral, dorsal, and ventrolateral areas of the quadrate. It is dorsally in contact with the *Musculus adductor mandibulae externus medialis* muscles and ventrally with the *M*. *depressor mandibulae* and the *M*. *cervicomandibularis*. The lateralmost and dorsalmost fibers originate via a tendon from the proximal epyphisis of quadrate, as well as from the cartilage associated with the epiphysis. In some species, fibers might originate from the dorsal and medial edges of the quadrate (but see discussion; see variation in S1 Table). Fiber orientation varies from (1) anteroposterior to (2) dorsoventral with and anterior orientation. It inserts (Fig 3D) via a flat rectangular tendon onto the supracotylar process of the compound bone. In a few species it and might also insert onto the posterior, lateral or dorsal areas of the supracotylar process of the compound bone (see variation in S1 Table).

#### *Musculus adductor mandibulae posterior* (S1 Table)

The muscle originates from the quadrates’ whole medial face or from specific areas of the bone (see variation in S1 Table). It provides a midventral coverage of the quadrate and of the posteromedial face of the compound bone. This muscle extends anteriorly or anterodorsally and inserts onto the medial prearticular face of the compound bone. Some fibers may attach onto the ventromedial face of the supracotylar process of the compound bone. It is present in some species of the genera *Epictia*, *Rena* and *Trilepida*, and in *Mitophis lepitepileptus* and *Tetracheilostoma bilineatum*, but absent in *Siagonodon cupinensis*. Inter- and intraspecific variations are listed in S1 Table.

#### *Musculus pseudotemporalis* (Fig 4A and 4C; S1 Table)

**Fig 4:**
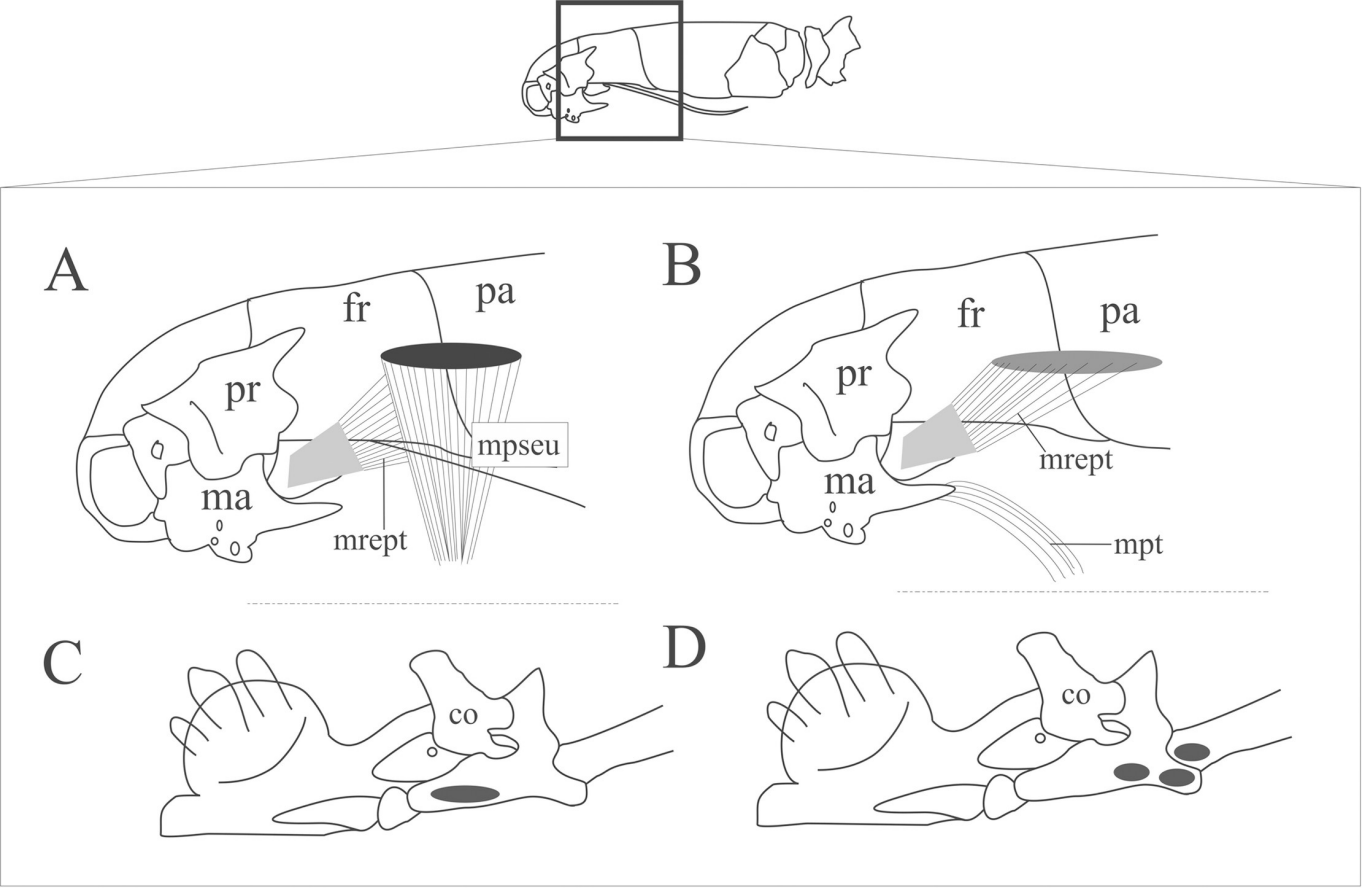
Schematic view of the location, origin and insertion sites of the *M*. *pseudotemporalis*, *M*. *retractor pterygoidei* and *M*. *pterygoideus* in Epitinae. The Harderian gland and all muscles lateral to it are removed. A: Lateral view of the head with origin area of the *M*. *pseudotemporalis* and insertion area of the *M*. *retractor pterygoidei*. B: Lateral view of the head with location, origin, and insertion areas of the *M*. *retractor pterygoidei* (*M*. *pesudotemporalis* removed) and location and site of origin of the *M*. *pterygoideus*. C: Medial view of the mandible with insertion site of the *M*. *pseudotemporalis* (black ellipse). D: medial view of mandible with the three possible insertion sites of the *M*. *pterygoideus*. Abbreviations: co = coronoid, fr = frontal, ma = maxilla, mpseu = *M*. *pseudotemporalis*, mpt = *M*. *pterygoideus*, mrept = *M*. *retractor pterygoidei*, pa = parietal, pr = prefrontal.

The *M*. *pseudotemporalis* is located medially to the Harderian gland (Fig 4A), providing a lateral coverage to the region of the skull that is immediately posterior to the orbit. It covers laterally a wide area of the *M*. *retractor pterygoidei* origin. The *M*. *pseudotemporalis* arises from the lateral descending face of the frontal and/or parietal (Fig 4A). The fibers of the muscle converge ventrally from its origin to a narrower insertion onto the ventromedial face of the prearticular lamina of the compound bone (Fig 4C). Inter- and intraspecific variations are listed in S1 Table.

#### *Musculus protractor pterygoidei* (Fig 5; S1 Table)

**Fig 5:**
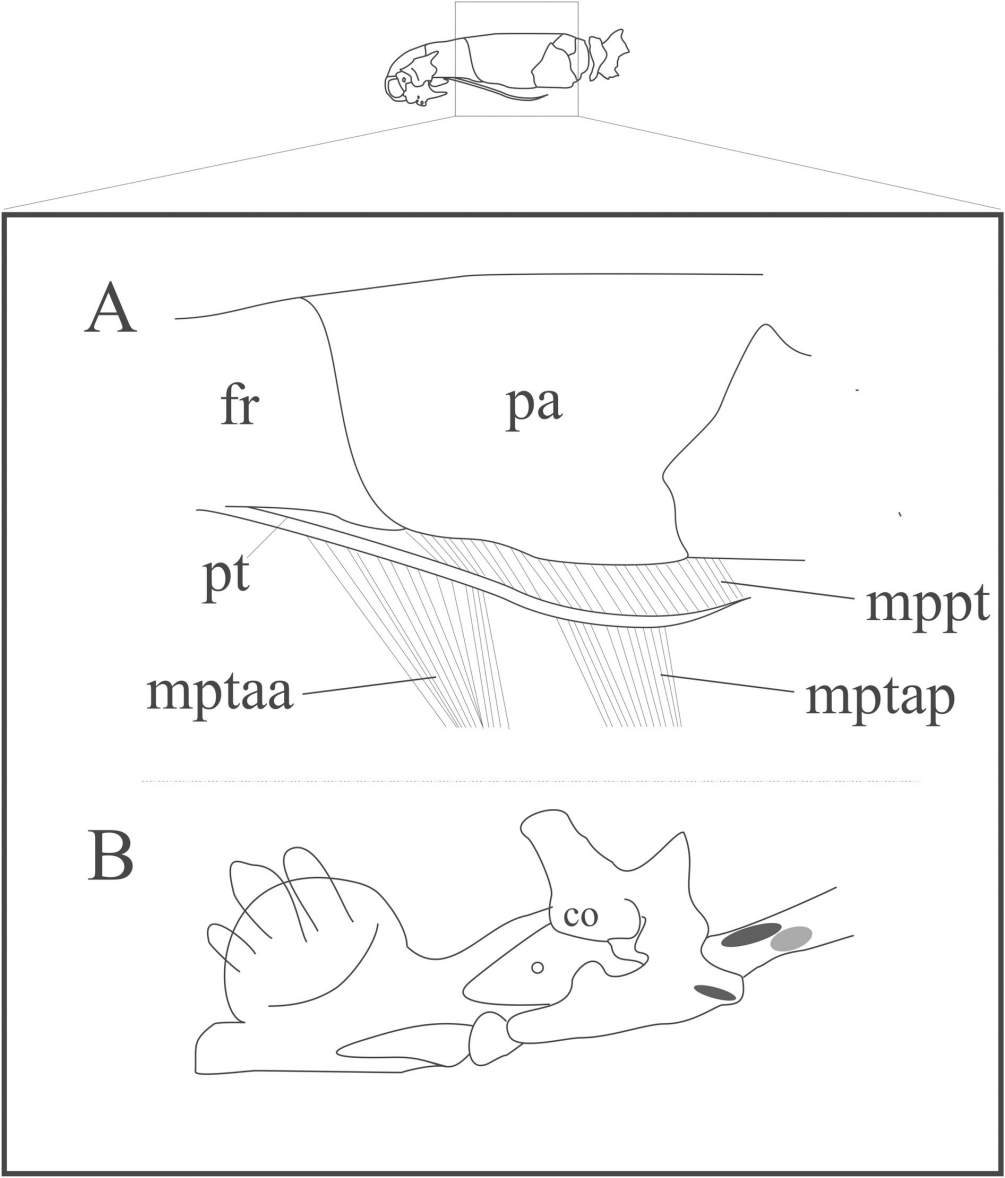
Schematic view of the location, origin and insertion sites of the *M*. *protractor pterygoidei*, *M*. *pterygoideus acessorius anterior* and *M*. *pterygoideus acessorius posterior* in Epictinae. A: Lateral view of the head with the location, origin, and insertion of the *M*. *protractor pterygoidei* and origins of the *M*. *pterygoideus acessoris anterior* and *posterior*. B: Medial view of the mandible with the possible insertion sites of the *M*. *pterygoideus acessorius anterior* (dark gray) onto the quadrate or compound bone, and the insertion site of the *M*. *pterygoideus acessorius posterior* (light gray) onto the quadrate. Abbreviations: co = coronoid, fr = frontal, mptaa = *M*. *pterygoideus acessorius anterior*, mptap = *M*. *pterygoideus acessorius posterior*, mppt = *M*. *protractor pterygoidei*, pa = parietal, pt = pterigoyd.

The muscle is flat and lies medially to the adductor muscles and the Harderian gland. Its thin and short fibers attach to the dorsal face of the pterygoid and to the lateroventral surface of the skull (Fig 5A). The origin extends from the ventrolateral face of the parietal and/or the parabasisphenoid, from the frontoparietal suture (in ventral view) to the posterior limit of the pterygoid (Fig 5A). Fibers are oriented posteroventrally inserting onto the dorsal face of the pterygoid. The insertion area may vary intra- and interspecifically (see S1 Table).

#### *Musculus retractor pterygoidei* (Fig 4B; S1 Table)

The muscle is located medially and posteriorly to the eye and the Harderian gland, providing a medial and ventral cover to these structures. Its origin is almost completely covered by the descending dorsal area of the *M*. *pseudotemporalis* (Fig 4A and 4B). The origin is either exclusively from the lateroventral face of the parietal (*E*. *ater*, *E*. *phenops*, *R*. *unguirostris*, *S*. *cupinensis*), exclusively from the frontal (*E*. *tenella*, *Tetracheilostoma bilineatum*), from both parietal and frontal (*M*. *lepitepileptus*) or from the parietal, frontal and pterygoid (*R*. *dulcis*, *R*. *humilis*, *R*. *segrega*, *Trilepida* spp.). Fibers insert onto the wide sheet of fibrous tissue located ventromedially to the eye, while the fibrous tissue attaches to the maxillary process of the palatine. Additional inter- and intraspecific variations are provided in S1 Table.

#### *Musculus pterygoideus* (Fig 4B and 4D; S1 Table)

The *M*. *pterygoideus* is a fusiform muscle almost completely covered by the lateral adductor mandibulae muscles as well as the Harderian gland. The muscle is located ventrally in the basicranium floor. Its origin is from the midposterior face of the maxilla and might be restricted to its posterior process (*M*. *lepitepileptus*, *Te*. *bilineatum*, *Trilepida* spp. except for *Tr*. *brasiliensis*), or to its posterior process as well as a narrow area anterior to it (*E*. *ater*, *E*. *phenops*, *S*. *cupinensis*; Fig 4B), or the origin is exclusively from an area anterior to the posterior process of maxilla (*E*. *tenella*, *Tr*. *brasiliensis*). It inserts (Fig 4D) onto the medial face of the retroarticular process (*Tr*. *jani*), retroarticular process + medially onto the articular face of the compound bone (*Tr*. *macrolepis*), or onto the quadrate (*R*. *unguirostris*, *S*. *cupinensis*, *Te*. *bilineatum*, *Tr*. *brasiliensis*, *Tr*. *dimidiata*, *Tr*. *fuliginosa*, *Tr*. *koppesi*, *Tr*. *salgueiroi*). Inter- and intraspecific variations are provided in S1 Table.

#### *Musculus pterygoideus acessorius anterior* (Fig 5; S1 Table)

This muscle represents a flat bundle of fibers that is located medially to the postorbital lobe of the Harderian gland, participating in the lateral protection of the skull-mandible gap. It originates from the ventral surface of the pterygoid process of the palatine or from the anteriormost surface of the pterygoid (Fig 5A). It inserts onto the retroarticular process of the compound bone or onto the anterior dorsomedial face of the quadrate (Fig 5B). Inter- and intraspecific variations are provided in S1 Table.

#### *Musculus pterygoideus acessorius posterior* (Fig 5)

This muscle represents a flat bundle of fibers that is located posteromedially to the postorbital lobe of the Harderian gland on the ventrolateral and posterior region of the skull, posterior to the *M*. *pseudotemporalis* and *M*. *retractor pterygoidei*. It covers the posterolateral region of the skull, medially and posteriorly to the postorbital lobe of the Harderian gland (Fig 5A). It is laterally covered by the *M*. *adductor externus medialis* portion C and *M*. *addcutor externus medialis profundus*. This muscle originates anteriorly from the ventrolateral surface of the parietal or from half its length, with some fibers possibly originating from the medial or ventral surface of the pterygoid or being restricted to this bone (Fig 5A). Fibers converge ventrally to insert onto the anterior region of the ventromedial surface of the quadrate, posterior to insertion of the *M*. *pterygoideus* (Fig 5B). It is absent in *Epictia ater*, *E*. *phenops*, *M*. *lepitepileptus*, *R*. *unguirostris*, *Te*. *bilineatum*, *Tr*. *dimidiata*, *Tr*. *fuliginosa*, *Tr*. *jani*, *Tr*. *joshuai*, *Tr*. *koppesi*, and *Tr*. *macrolepis*. Inter- and intraspecific variations are provided in S1 Table.

### Extrinsic ocular muscles (Fig 6; Table 1)

The extrinsic ocular muscles are thin and delicate bundles of parallel and rectangular muscle fibers located medially to eye. Five extrinsic muscles were identified in Epictinae: *M*. *obliquus superior*, *M*. *obliquus inferior*, *M*. *rectus* s*uperior*, *M*. *rectus posterior* and *M*. *rectus inferior* (Table 1); that is *M*. *rectus anterior* is missing in Epictinae. None of those muscles were recorded in *E*. *ater*, *R*. *unguirostris* and *Tr*. *dimidiata*.

**Fig 6:**
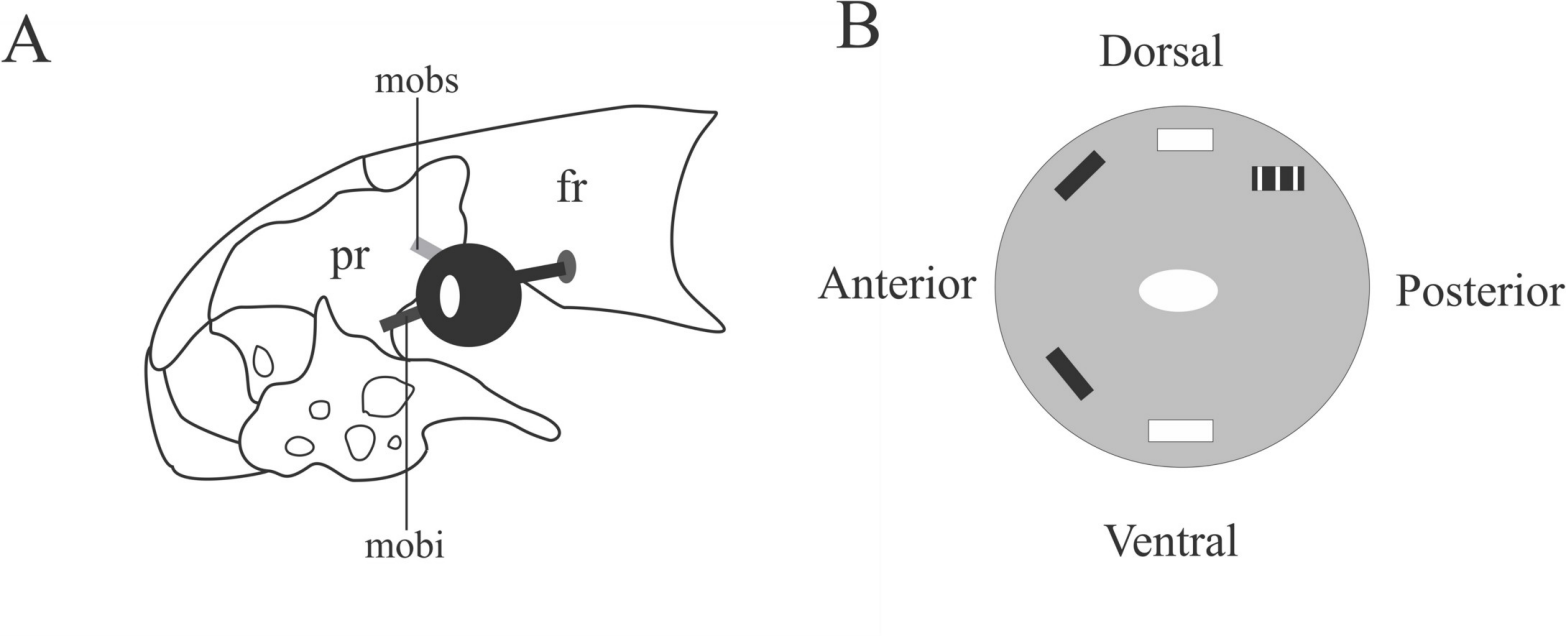
Schematic view of the location and insertion sites of the extrinsic eye muscles in Epictinae. A: Lateral view of the head with the location of extrinsic eye muscles. B: Posterior view of the eye with muscle insertion sites. fr: frontal; mobs: *Musculus obliquus superior*; mobi: *M*. *obliquus inferior*; pr: prefrontal. Black rectangle: insertion sites for the *M*. *obliquus superior* (top) and *M*. *obliquus inferior* (bottom). White rectangles: insertion sites for the *M*. *rectus superior* (top) *M*. *rectus inferior* (bottom). Black and white rectangle: insertion site of the *M*. *rectus posterior*. White ellipse: insertion of the optic nerve.

#### *Musculus obliquus superior* (Fig 6A and 6B; Table 1)

This muscle is located anterodorsally to eye. When present, it originates from the descendent posterodorsal face of the prefrontal or from the ventral face of the posterior process of the prefrontal. It extends posteroventrally or ventrally and inserts onto the medial face of the eye. This muscle is absent or indistinct in *E*. *ater*, *E*. *phenops*, *R*. *humilis*, *R*. *segrega*, *Tr*. *dimidiata* and *Tr*. *jani*. Interspecific variations are listed in Table 1.

#### *Musculus obliquus inferior* (Fig 6; Table 1)

The muscle is located anteroventrally to the eye. When present, it originates from the posterodorsal face of the prefrontal at its ventralmost region. The fibers extend dorsoposteriorly, ventroposteriorly or completely ventrally to inserting onto the medial face of the eye at its anteroventral area. This muscle is absent or indistinct in *M*. *lepitepileptus* and *S*. *cupinensis*, as well as in some *Trilepida* species (*Tr*. *fuliginosa*, *Tr*. *dimidiata*, *Tr*. *joshuai*). Interspecific variations are provided in Table 1.

#### *Musculus rectus superior* (Fig 6B; Table 1)

The muscle is located dorsoposteriorly to eye. It originates from the dorsoposterior process of the prefrontal, extending ventroposteriorly to insert onto the medial face of the eye at its dorsoposterior area. It is present in *Tr*. *joshuai*, *Tr*. *koppesi*, *Tr*. *jani*, *R*. *dulcis*, *E*. *phenops*, *E*. *tenella* and *M*. *lepitepileptus*. Interspecific variations are provided in Table 1.

#### *Musculus rectus posterior* (Fig 6B; Table 1)

The muscle is located dorsoposteriorly to the eye. Its occurrence varies interspecifically amongst Epictinae, being present in *E*. *phenops*, *R*. *humilis* and *Tr*. *joshuai*. We could not determine its precise origin through manual dissections, but it lies adjacent to the optic nerve, descends anteriorly to insert onto the midposterior face of the eye. Interspecific variations are provided in Table 1.

#### *Musculus rectus inferior* (Fig 6B; Table 1)

The muscle is located ventromedially to the eye. It is exclusively present in *Tr*. *jani* and *Tr*. *koppesi*. We could not determine its precise origin through manual dissections, but it lies adjacent to the optic nerve. It descends anteriorly and inserts onto the medial posterior surface of the eye. Interspecific variations are provided in Table 1.

#### *Musculus intermandibularis anterior* (Fig 7; S1 Table)

The *M*. *intermandibularis anterior* is a flat and narrow bundle of fibers located on the anteroventral region of the jaw posterior to the mandibular symphysis. This muscle is ventrally covered by the *M*. *costocutaneus superior* and it covers a small anterior region of the *M*. *intermandibularis posterior*, *pars anterior*. The *intermandibularis anterior* muscle provides, together with the ventral consctrictor muscles, a ventral support to the mouth The *M*. *intermandibularis anterior* originates from the ventromedial surface of the dentary. It inserts onto the subcutaneous muscles of the skull medial line, at the level of dentary’s dorsoposterior process until the posterior limit of the compound bone. We were not able to precisely obtain its exact site of insertion considering the damage that occured with skin removal; thus, in some specimens, this muscle was completely damaged. Inter- and intraspecific variations are provided in S1 Table.

**Fig 7:**
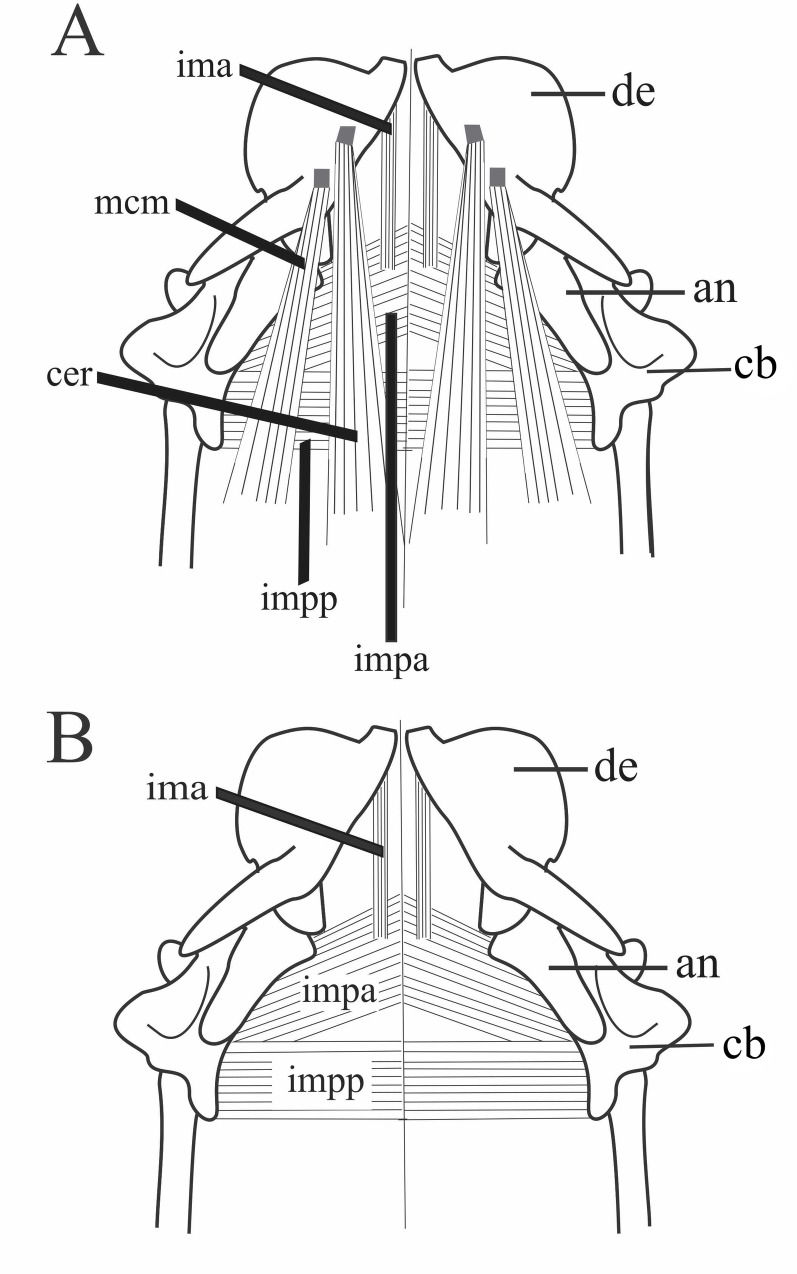
Schematic view of the head in ventral view, illustrating the ventral muscles after removal of the *M*. *costocutaneus superior* (A) and after removal of the *M*. *cervicomandibularis* and *M*. *ceratomandibularis* (B). Abbreviations: an = angular, cer = *M*. *ceratomandibularis*, de = dentary, ima = *M*. *intermandibularis anterior*, impa = *M*. *intermandibularis posterior pars anterior*, impp = *M*. *intermandibularis posterior*, *pars posterior*, mcm = *M*. *cervicomandibularis*, cb = compound bone.

#### *Musculus intermandibularis posterior*, *pars anterior* (Fig 7; S1 Table)

In most of the species, except for *S*. *cupinensis*, the muscle is flat and wide, located on the anteroventral region of the jaw. It is ventrally covered by the anterior fibers and the tendon of the *M*. *cervicomandibularis* and by the *M*. *ceratomandibularis*. Furthermore, it is covered anteriorly by the *M*. *intermandibularis anterior* and partially posteriorly by the *M*. *intermandibularis posterior*, *pars posterior*. This muscle provides a ventral support to the mouth together with the *constrictor* muscles adjacent to it. It originates laterally and inserts medially. The fibers are oriented from posterolateral to anteromedial and the muscle inserts into a medial raphe with its contralateral part via a tendinous portion. Two distinct portions of the *M*. *intermandibularis posterior*, *pars anterior* are present in *S*. *cupinensis*.

#### *Musculus intermandibularis posterior*, *pars posterior* (Fig 7; S1 Table)

This is a wide and flat muscle located superficially on the ventral region of the head. Its superficial location and its loose connection to adjacent connective tissue hampers its dissection and description. Consequently, this muscle was damaged during dissection in several specimens and the only valuable data are described below. The *M*. *intermandibularis posterior*, *pars posterior* rests under the *M*. *costocutaneus superior*. It extends transversally from the lateroventral border of the mandible, crossing over an anterior aponeurotic area of the *M*. *depressor mandibulae* and under anterior aponeurotic areas of the *M*. *cervicomandibularis*. The muscle fibers also extend over the *M*. *ceratomandibularis* until inserting into the medial raphe Interspecific variations are provided in S1 Table.

#### Musculus costocutaneus superior

The *M*. *costocutaneus superior* is a hypobranchial-spinal muscle with fibers coalescing and comprising a continuous subcutaneous layer on the head. The muscle description was based exclusively on *Tr*. *koppesi* and *R*. *humilis*, because these were the only specimens where the muscle was not damaged during dissection. This muscle covers the ventral and lateral area of the head. It originates from (1) the frontoparietal suture, (2) laterally via a narrow aponeurosis attaches to the dorsolateral face of frontal, and (3) ventrally via a wide aponeurosis that attaches to the ventral face of mandible. It inserts onto the adjacent scales posterior to the head.

#### *Musculus cervicomandibularis* (Figs 2 and 7; S1 Table)

The *M*. *cervicomandibularis* is a triangular muscle usually covered by the *M*. *costocutaneus superior*. It covers the posteroventral region of the skull at the level of the compound bone, extending posteriorly to a short a dorsolateral area that lies posterior to the skull (= i.e cervical or anterior trunk vertebrae). It also covers the *M*. *depressor mandibulae* laterally. Its origin varies and might occur via (1) a single tendon from the dentary, (2) a bifid tendon from two distinct areas of dentary or (3) a wide tendon from the dentary or retroarticular process of the compound bone (see S1 Table). Its insertion might be single or double onto the cutaneous muscles at the level of the anterior thoracolumbar vertebra. When double, the anterior portion inserts onto the subcutaneous muscles adjacent to the cervical vertebrae, while the posterior portion exceeds the posterior limits of the cervical vertebrae.

#### *Musculus depressor mandibulae* (Fig 2)

The *Musculus depressor mandibulae* is a fusiform muscle with most of its fibers covered by the adductor muscles that lie posteroventrally to the skull, the *M*. *cervicomandibularis*, *M*. *aducctor mandibulae externus profundus* and *M*. *cervicoquadratus*. It is located ventrally to the quadrate and originates from the posteromedial edge of the proximal epiphysis of the quadrate and medially from the cartilage associated with the proximal epiphysis. This muscle inserts *via* a tendon onto the posteromedial face of the retroarticular process of the compound bone.

### Unnamed muscle 1

This fusiform muscle named herein as “Unnamed muscle 1” is only present in *R*. *humilis* (SDSNH 33950) and *E*. *tenella* (MCZ 60801). It is located medially to Harderian gland and the muscles *M*. *pseudotemporalis* and *M*. *retractor pterygoidei*, and crosses the mouth dorsally to reach the anterior area of the glottis. It originates from the medial face of the posterior process of the maxilla, from the tendon of the *M*. *pterygoideus*. It extends posteriorly and medially over the *M*. *pseudotemporalis* and *M*. *retractor pterygoidei* inserting onto the dorsal layer of the glottis, posterior to the parabasisphenoid.

### Unnamed muscle 2 (Table 2)

This delicate and thin muscle herein called “Unnamed muscle 2” is immersed in the anterior intermandibular area and contributes to the dorsal coverage of the trachea. It originates from the medial face of the symphyseal process of the dentary. This muscle enlarges as it extends medially towards the ventral intermandibular muscles (*M*. *intermandibularis anterior* and *M*. *intermandibularis posterior*, *pars anterior*), inserting dorsally onto the anterior rings of trachea from the 1^st^ to 7^th^ tracheal ring, with different areas of insertion (see Table 2 for variations). In most individuals, the precise description of insertion (correspondent to ring number) was not possible due to the small size of specimens.

**Table 2:** Summary of inter- and intraspecific variability of the insertion of the *“*Unnamed muscle 2” onto the tracheal rings. n = number of specimens.

Species	Tracheal rings
*Epictia tenella*	2nd– 5th (n = 1) or 4th to 7th (n = 1)
*Rena humilis*	1st– 4th (n = 1)
*Rena segrega*	2nd– 3rd (n = 1)
*Rena unguirostris*	1st– 3rd (n = 1)
*Trilepida brasiliensis*	3rd– 5th (n = 1)
*Trilepida dimidiata*	3rd– 5th (n = 1)
*Trilepida fuliginosa*	3rd– 4th (n = 1)
*Trilepida jani*	2nd– 6th (n = 1)
*Trilepida joshuai*	1st– 4th (n = 1)
*Trilepida koppesi*	3rd– 6th (n = 1)
*Trilepida macrolepis*	4th– 6th (n = 1)
*Trilepida salgueiroi*	4th– 6th (n = 1)

#### *Musculus geniotrachealis* (Fig 8; Table 3)

The *M*. *geniotrachealis* is flat and, as the *M*. *geniomucosalis*, crosses the inner region of the mouth floor towards the trachea. This muscle originates from the medial face of the dental concha, enlarging towards its insertion. It inserts onto the tracheal rings (4th–14th ring), with both inter- and intraspecific variation (see Table 3 for variations).

**Fig 8:**
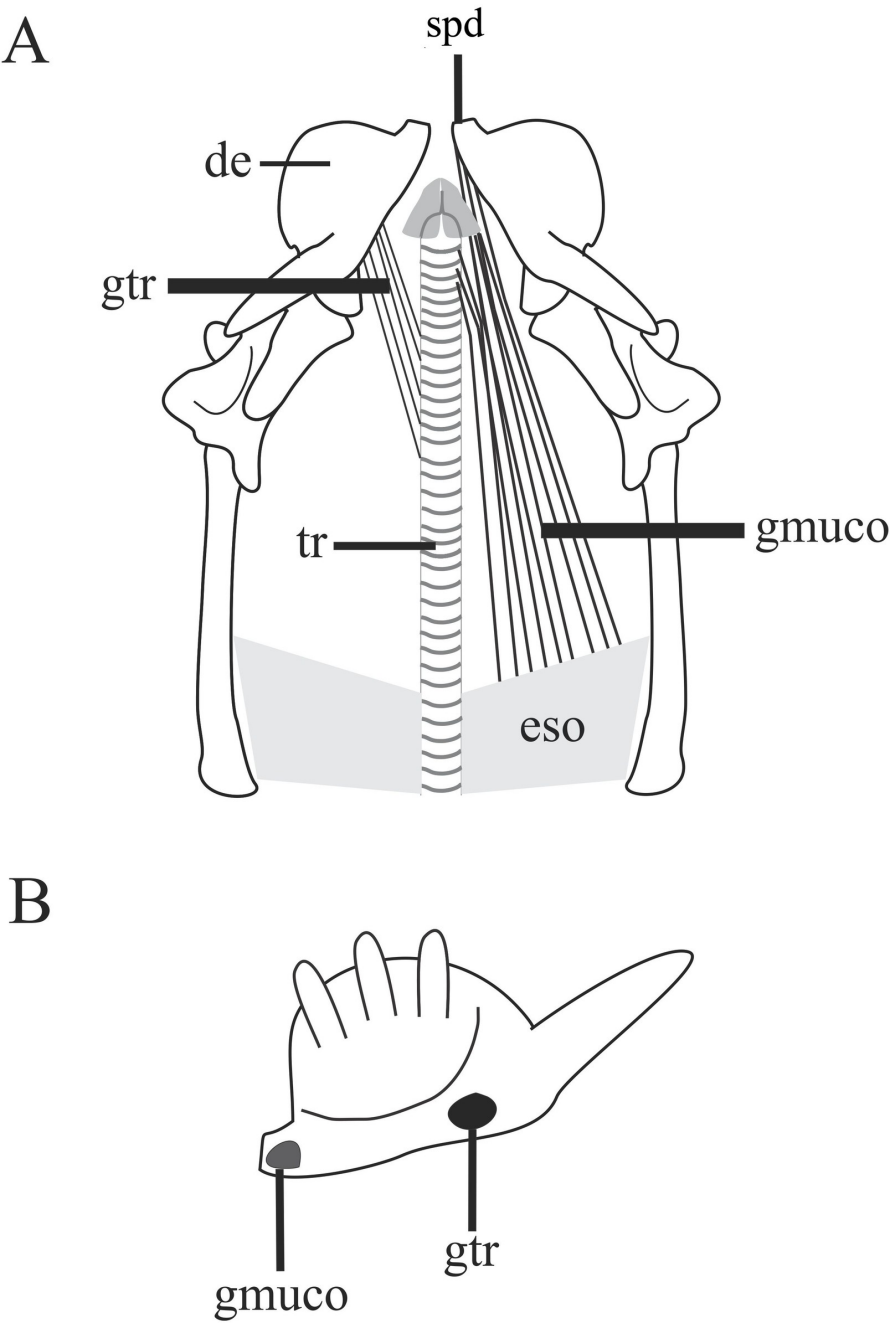
Schematic view of the head in ventral view with removal of the *M*. *contrictores ventrales*, *M*. *ceratomandibularis*, *M*. *cervicomandibularis*, *M*. *genioglossus* and tongue to illustrate the insertion and location of the *M*. *geniotrachealis* and *M*. *geniomucosalis* (A) and their origin sites from the medial face of the dentary (B). Abbreviations: de = dentary, gmuco = *M*. *geniomucosalis*, gtr = *M*. *geniotrachealis*, spd = symphyseal process, tr = trachaea. N = number of specimens.

**Table 3:** Summary of inter- and intraspecific variability of the insertion of the *Musculus geniotrachealis* onto the tracheal rings. n = number of specimens.

Species	Tracheal rings
*Epictia ater*	4th– 5th (n = 1)
*Epictia tenella*	7th– 10th (n = 1) or 10th -18th (n = 1)
*Epictia phenops*	11th– 15th (n = 1)
*Mitophis lepitepileptus*	Damaged or absent (n = 1)
*Rena dulcis*	6th– 9th (n = 1) or 8th– 10th (n = 1)
*Rena humilis*	7th– 11th (n = 1)
*Rena segrega*	4th– 9th (n = 1)
*Rena unguirostris*	8th– 10th (n = 1)
*Siagonodon cupinensis*	9th– 10th (n = 1)
*Tetracheilostoma bilineatum*	8th– 13th (n = 1)
*Trilepida brasiliensis*	6th– 11th (n = 1)
*Trilepida dimidiata*	8th– 12th (n = 1)
*Trilepida fuliginosa*	3rd– 4th (n = 1)
*Trilepida jani*	11th– 14th (n = 1)
*Trilepida joshuai*	9th– 14th (n = 1)
*Trilepida koppesi*	3rd– 6th (n = 1)
*Trilepida macrolepis*	6th– 13th (n = 1)
*Trilepida salgueiroi*	6th– 13th (n = 1)

#### *Musculus genioglossus* (Fig 9)

The *M*. *genioglossus* is wide and flat, and surrounds the ventral and lateral sides of the tongue at the level of a tendinous sheet. Fibers of the *M*. *genioglossus* do not blend with the tongue fibers. This muscle is located ventral to the head with each *M*. *genioglossus* extending posteriorly into the tongue, from the anterior limit of the mandible until the hyoid. It originates from the mandible symphysis through a long and tendon that narrow posteriorly inserting onto the hyoid cornua. No inter- or intraspecific variations were observed.

**Fig 9:**
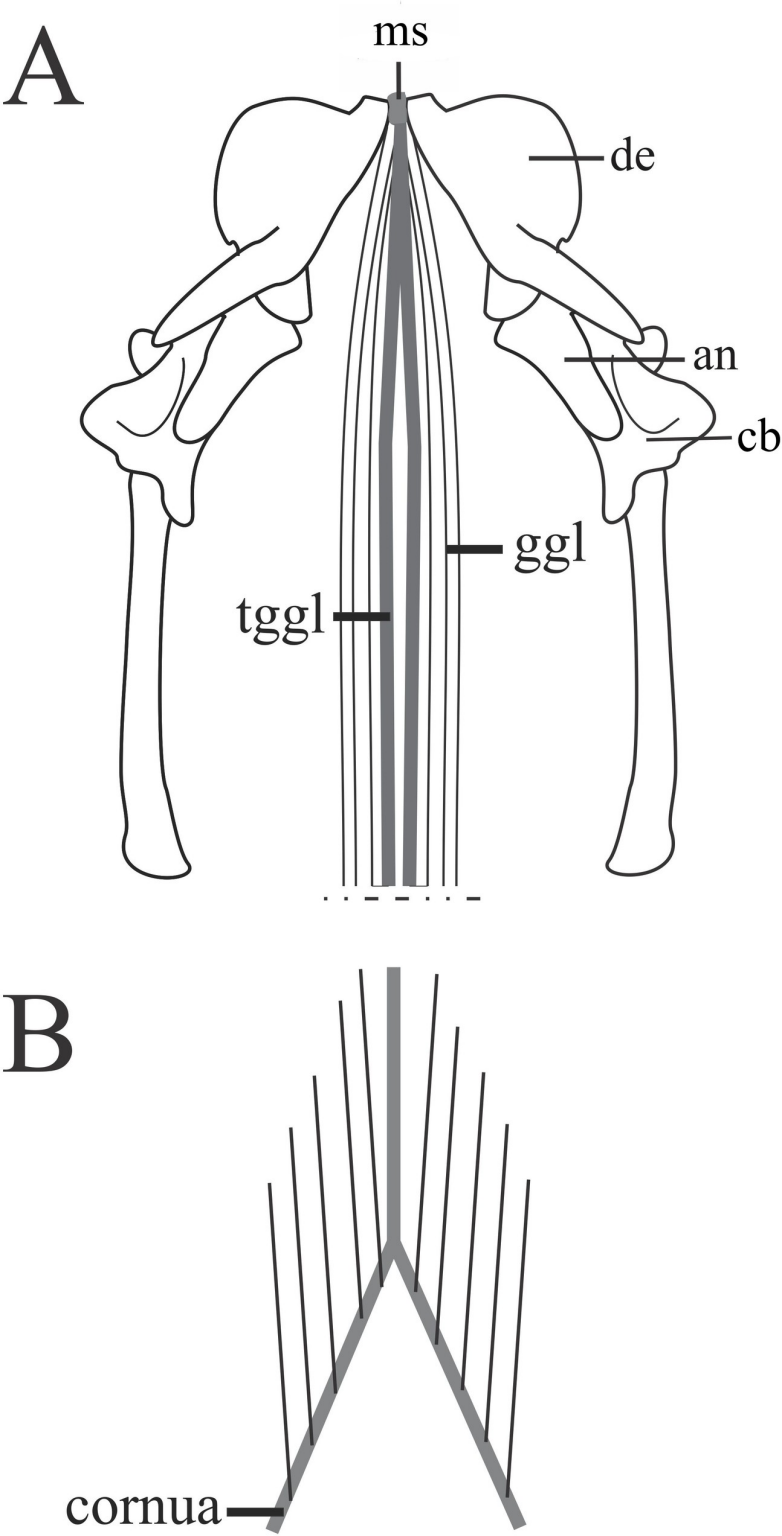
Schematic view of the head in ventral view after removal of the ventral constrictors, *M*. *ceratomandibularis*, *M*. *cervicomandibularis*, illlustrating the location, origin, and insertion sites of the *M*. *genioglossus*. A: Origin of the *M*. *genioglossus*. B: insertion of the *M*. *genioglossus* in the hyoid. Abbreviations: an = angular, de = dentary, cb = compound bone, ggl = *M*. *genioglossus*, ms = mandibular symphysis, tggl = *M*. *genioglossus* tendon.

#### *Musculus geniomucosalis* (Fig 8; Table 4)

The *M*. *geniomucosalis* is a flat and bifurcated, extending lateroventrally layer to the esophagus and trachea. It is immersed in the bundle of muscles around the tongue, being completely covered by the *constrictores ventrales*. Its lateral portion originates from the medial face of the symphyseal process of the dentary, anterior to the *M*. *geniotrachealis* insertion (Fig 8A). Its medial portion originates from the ventrolateral layer of the glottal tube and extends anteriorly to the mandibular symphysis or arythenoid cartilages of the larynx. Its area of origin varies both inter- and intraspecifically (see Table 4). Its insertion is anteriorly onto the mouth roof and extends posteriorly to esophagus and nuchal muscles (Fig 8A and 8B).

**Table 4:** Summary of inter- and intraspecific variability of the origin of the *Musculus geniomucosalis* from the larynx and tracheal rings.

Species	Origin on larynx and tracheal rings
*Epictia ater*	Arythenoid cartilages, 1st– 10th ring
*Epictia tenella*	Arythenoid cartilages, 1st– 3rd ring
*Epictia phenops*	Damaged or absent
*Mitophis lepitepileptus*	Damaged but present
*Rena dulcis*	Crycoid cartilage
*Rena humilis*	Crycoid cartilage
*Rena segrega*	Crycoid cartilage
*Rena unguirostris*	Crycoid cartilage, 1st– 7th ring
*Siagonodon cupinensis*	4th– 1oth ring
*Tetracheilostoma bilineatum*	Cricoid cartilage, 1st– 10th ring
*Trilepida brasiliensis*	1st ring
*Trilepida dimidiata*	Cricoid cartilage, 1st– 5th ring
*Trilepida fuliginosa*	Cricoid cartilage, 1st– 2nd ring
*Trilepida jani*	Cricoid cartilage, 1st– 13th ring
*Trilepida joshuai*	Cricoid cartilage, 1st– 5th ring
*Trilepida koppesi*	Cricoid cartilage, 1st– 8th ring
*Trilepida macrolepis*	Damaged but present
*Trilepida salgueiroi*	Cricoid cartilage, 1st– 5th ring

#### *Musculus ceratomandibularis* (Fig 7A; S1 Table)

The *M*. *ceratomandibularis* is located in the ventral region of the head and neck, being supported by the tongue muscles, and dorsally and laterally by the *M*. *genioglossus*. This muscle is completely covered by *M*. *costocutaneus superior* and *M*. *intermandibularis posterior*, *pars posterior*. It originates from the lateral face of the dentary via a tendon that is usually short, flat and rectangular and attaches to the dental concha, the dorsoposterior process of the dentary or the body of the dentary. The *M*. *ceratomandibularis* is composed of two (*M*. *lepitepileptus*, *R*. *unguirostris*, *Tr*. *jani*) or three (*Epictia* spp., *R*. *dulcis*, *R*. *humilis*, *R*. *segrega*, *S*. *cupinensis*, *Te*. *bilineatum*, *Trilepida* spp.) portion of fibers. There can be up to three portions: lateral, middle and medial. Its middle portion is present in all examined species (except for *Tr*. *jani*), extends and narrows posteriorly and inserts onto the cornua or onto the lingual process of the hyoid. When present, a medial portion inserts into a median raphe with the contralateral *ceratomandibularis*. A lateral portion might be present inserting onto the subcutaneous muscles and onto the muscles associated with the ribs. Interspecific variations are provided in S1 Table.

### Neck myology

#### *Musculus cervicoquadratus* (Fig 10; S1 Table)

This triangular and flat muscle is located in the occipital and anteroventral nuchal areas, being covered by the *M*. *cervicomandibularis* in its proximal area. It covers the *M*. *ceratomandibularis* on the ventral nuchal region. It originates from the proximal epiphysis of the quadrate and/or from the lateral face of the prootic. Inter- and intraspecific variations are provided in S1 Table.

**Fig 10:**
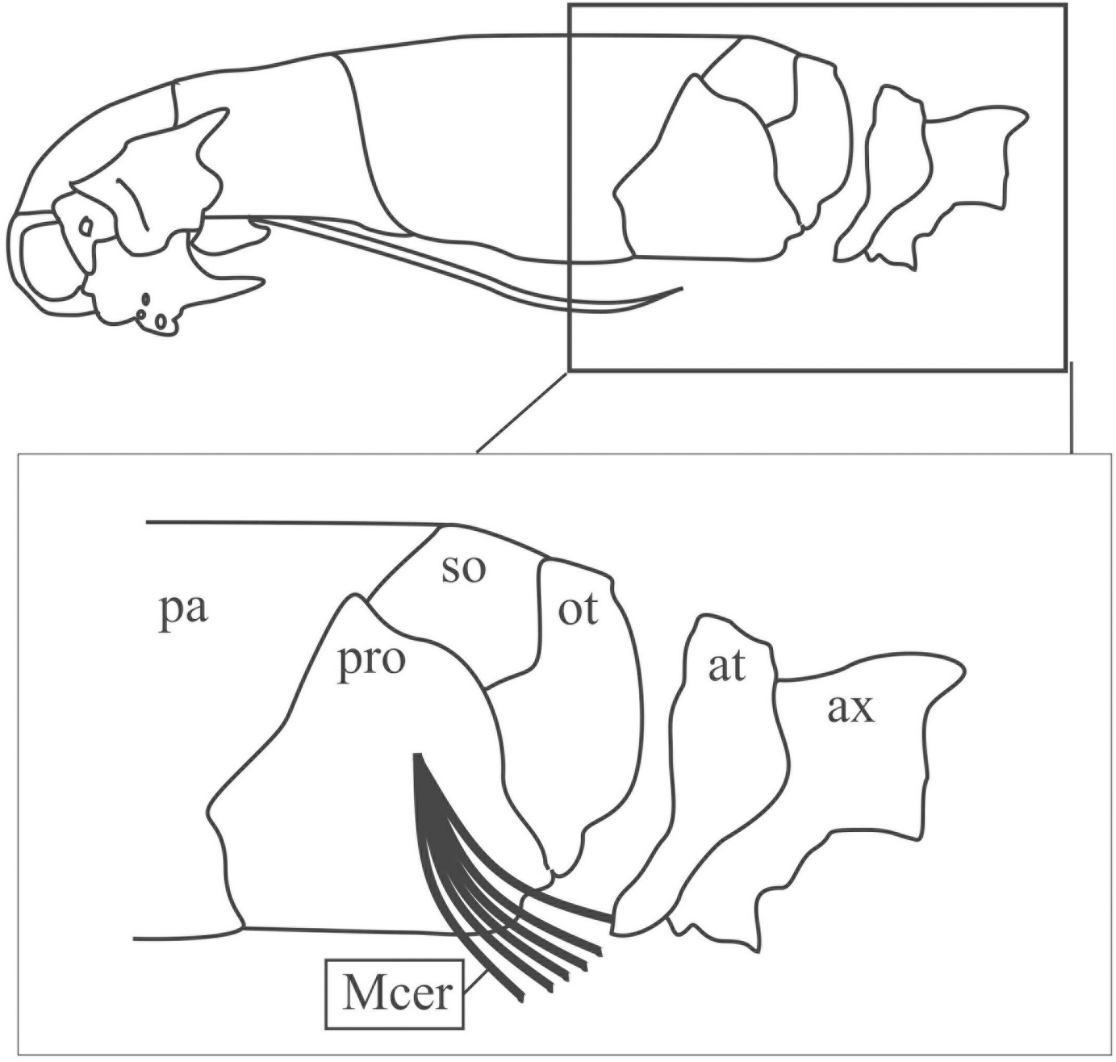
Schematic view of the head in lateral view with insertion site of the *M*. *cervicoquadratus*. Abbreviations: at = atlas, ax = axis, mcer = *M*. *cervicoquadratus*, ot = otooccipital, pa = parietal, pro = prootic, so = supraoccipital.

#### *Musculus spinalis et semispinalis capitis* (Fig 11; Table 5)

This is a neck muscle that belongs to the *M*. *transversospinalis* group muscles and represents the most medial muscle of the epaxial musculature. It is composed of the *M*. *spinalis capitis* and *M*. *semispinalis capitis*. This complex is located ventral to the *M*. *costocutaneus superior* and the subcutaneous muscles, covering the post-cranial dorsal and dorsolateral areas of the cervical and anterior thoracolumbar vertebrae. The *M*. *spinalis capitis* is medial and dorsal to the *M*. *semispinalis capitis*. The *M*. *spinalis capitis* originates from the *M*. *semispinalis* tendon, which attaches to the spinal process of the axis (cervical vertebrae C2 = V2). The extension of fibers that originate from the tendon varied interspecifically in the species analyzed herein (see Table 5). The *M*. *semispinalis capitis* originates from the *M*. *longissimus* tendon that attaches to the anterior area of the vertebrae’s *centrum*; the expansion of origin also shows variation from V3 to V27 (Table 6). The variations in the origins of both *M*. *spinalis capitis* and *M*. *semispinalis capitis* are listed in Table 5. The *M*. *spinalis capitis* inserts via a wide and flat tendon that may be “P”-shaped, "T"-shaped or triangular (Table 5) onto the posterior limit of the parietal.

**Fig 11:**
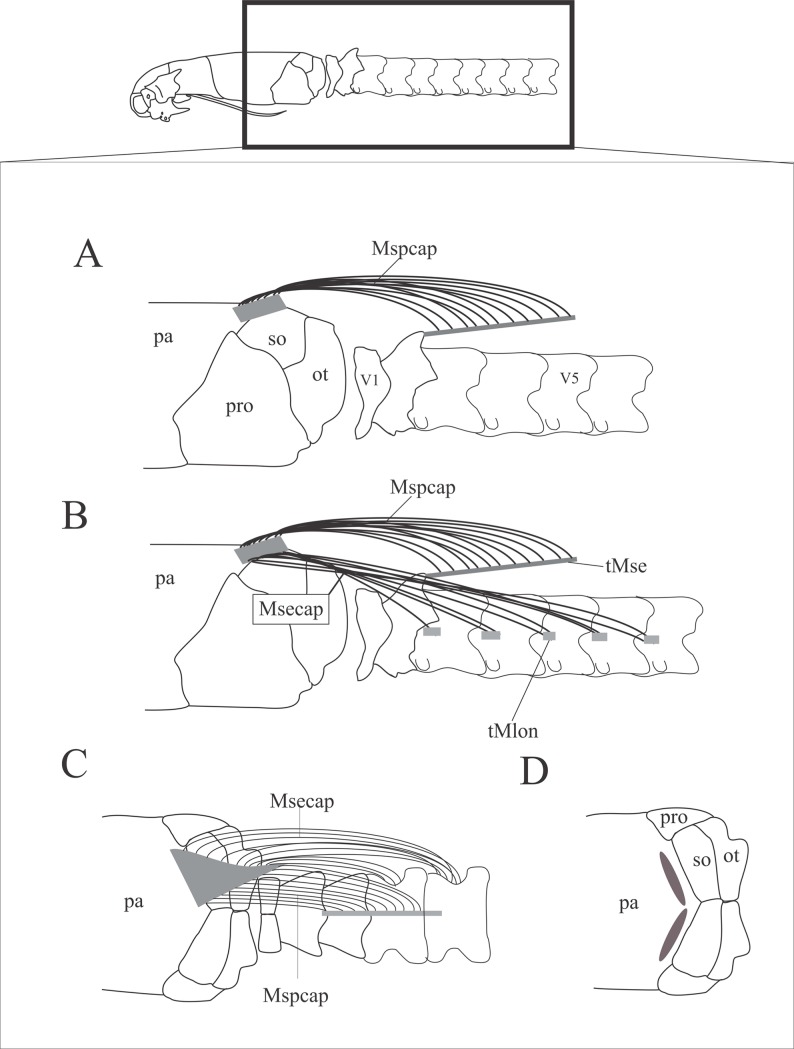
Schematic view of the location, origin and insertion sites of the *M*. *spinalis et semispinalis* capitis. A: Lateral view of the head and neck areas, with illustration of the *M*. *spinalis capitis* (A) and *M*. *spinalis et semispinalis capitis* (B) and dorsal view with location (C) and origin site (C,D) of the *M*. *spinalis et semispinalis*. Abbreviations: ot = otooccipital, pa = parietal, pro = prootics, so = supraoccipital, “V” = vertebrae of origin where V1 = atlas, V2 = axis, V3 = first thoracolombar vertebra and so on.

**Table 5:** Variability of the origin area of the *M*. *spinalis et semispinalis capitis* muscles and shape of insertion tendon for members of subfamily Epictinae. Symbols and abbreviations: 1 = posterior limit of *M*. *semispinalis* tendon; 2 = posterior limit of the *M*. *spinalis capitis* fibers along the *M*. *semispinalis* tendon; 3 = area of insertion of the *M*. *semispinalis capitis*; 4 = shape of the *M*. *spinalis et semispinalis capitis* tendon of iinsertion; “V” = vertebrae of origin where V1 = atlas, V2 = axis, V3 = first thoracolombar vertebra and so on.

Species	1	2	3	4	Notes
*Epictia ater*	V4	V4	V3 –V9	T-shaped	-
*Epictia tenella*	V3 or V5	V3 or V5	V6-V9 or V2-V8	T or P-shaped	In one specimen (50%) independent fibers of the *M*. *spinalis capitis* attach directly to V1, and ventral fibers of the *M*. *semispinalis* capitis attaches to dorsal fibers of the *M*. *longissimus capitis*, *pars transversalis capitis* and *M*. *transversalis*.
*Epictia phenops*	V5	V5	V3-V8	T-shaped	-
*M*. *lepitepileptus*	V5	V5	V3-V6	P-shaped	*M*. *spinalis capitis* attaches throughout the whole tendon of *M*. *semispinalis capitis*. Some posterior fibers of the *M*. *spinalis capitis* exceeds *M*. *semispinalis* tendon attaching to *M*. *transversalis* tendon from V6–V8
*Rena dulcis*	V6	V5	V3- V8	P-shaped	-
*Rena humilis*	V7 or V6	V7 or V5	V3-V6 or V2-V4	P-shaped	-
*Rena segrega*	V5	V5	V3-V9	P-shaped	A few *M*. *semispinalis capitis* fibers extend posterior-dorsally attaching to tendon of the transversal trunk muscles, from V5–V8.
*Rena unguirostris*	V5	V3	V3-V6	P-shaped	-
*S*. *cupinensis*	V5	V5	V3-V7	Triangular	-
*Te*.*bilineatum*	V5	V5	V3-V7	P-shaped	-
*Tril*.*brasiliensis*	V5	V5	V2-V5	P-shaped	-
*Trilepida dimidiata*	V5	V5	V2- V10	P-shaped	-
*Trilepida fuliginosa*	V6	V6	V3-V9	P-shaped	-
*Trilepida jani*	V7	V7	V3-V6	P-shaped	-
*Trilepida joshuai*	V5	V5	V4-V9	P-shaped	-
*Trilepida koppesi*	V5	V4	V3-V11	P-shaped	-
*Trilepida macrolepis*	V5	V4	V3-V8	P-shaped	-
*Trilepida salgueiroi*	V4	V3	V3-V10	Subtriangular	-

#### *Musculus longissimus capitis*, *pars transversalis capitis* (Fig 12; Table 6)

This muscle is located in the laterodorsal area of the neck, covering the occipital region ventral to the *M*. *semispinalis capitis*. The medial fibers originate directly from the *centrums* of the cervical and anterior thoracolumbar vertebrae, V2 –V13 (Table 6). Stouter muscle portions originate from the tendons that attach to postzygapophysis of the vertebrae V1 –V12 (Table 6). It inserts via a tendon onto the occipital region of the skull and might also insert onto the prootics posteriorly or anteriorly, otooccipitals anterolaterally, supraoccipitals lateroposteriorly or parietal lateroposteriorly (Table 6).

**Fig 12:**
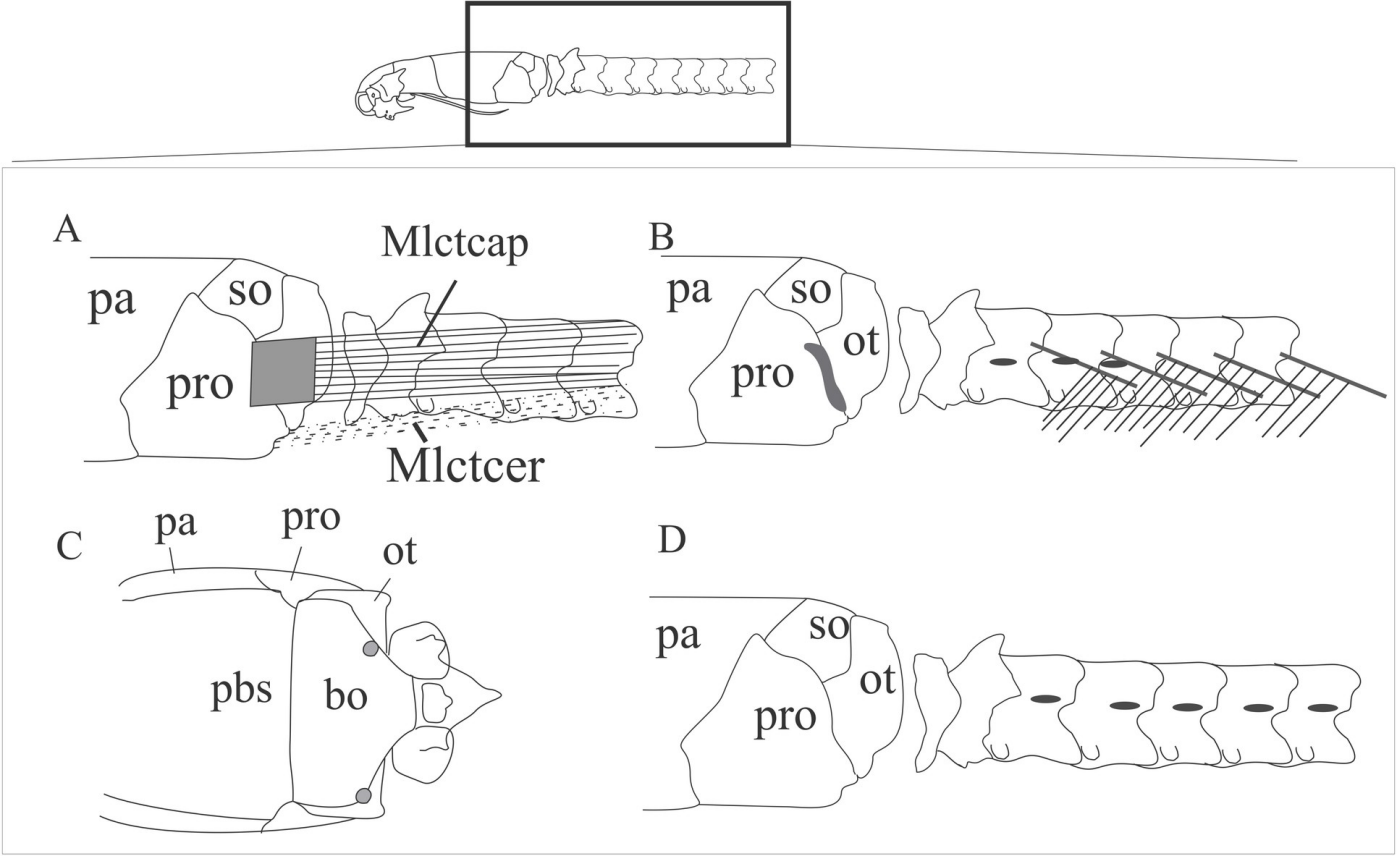
Schematic view of the location, origin and insertion of the *M*. *longissumus capitis*, *pars transversalis capitis* and the *M*. *longissumus capitis*, *pars transversalis cervicis*. A: location of the *M*. *longissumus capitis*, *pars transversalis capitis* and the *M*. *longissumus capitis pars transversalis*, *cervicis*. B: insertion site of the tendon of the *M*. *longissumus capitis*, *pars transversalis capitis* in the skull and its origin sites in the vertebrae and tendons. C: insertion site of the *M*. *longissumus capitis pars transversalis*, *cervicis* in the basioccipital. D: origin site of the *M*. *longissumus capitis pars transversalis*, *cervicis* in the vertebra. Abbreviations: bo = basioccipital, mlctcap = *M*. *longissimus capitis pars transversalis capitis*, mlctcer = *M*. *longissumus capitis pars transversalis cervicis*, ot = otooccipital, pa = parietal, pbs = parabasisphenoid, pro = prootic, so = supraoccipital.

**Table 6:** Variability related to origin and insertion of the *M*. *longissimus capitis*, *pars transversalis capitis* for members of Subfamily Epictinae. Symbols and abbreviations: 1 = Anteriormost origin of fibers of the *M*. *longissimus capitis*, *pars transversalis capitis* in tendons associated to the postzygapophyses; 2 = posteriormost origin of the *longissimus capitis*, *pars transversalis capitis* in tendons associated to the postzygapophyses; 3 = anteriormost fibers origin of the *M*. *longissimus capitis*, *pars transversalis capitis* in the vertebrae *centrum*; 4 = posteriormost origin of the *M*. *longissimus capitis*, *pars transversalis capitis* in the vertebrae *centrum*; 5 = insertion area of the *M*. *longissimus capitis*, *pars transversalis capitis* in the skull. “?” = indicates the impossibility of examination due to specimen damage, “V” = vertebrae of origin where V1 = atlas, V2 = axis, V3 = first thoracolombar vertebra and so on.

Species	1	2	3	4	5
*Epictia ater*	V2	V8	?	?	Prootic, Otooccipital, Supraoccipital
*Epictia tenella*	V2 or V1	V10 or V12	V3	V12	Prootic, Otooccipital, Supraoccipital or exclusively onto prootic, dorsal to the proximal epyphisis of quadrate
*Epictia phenops*	V1	V10	V3	V10	Prootic, posterior to proximal epyphisis of quadrate
*Mitophis lepitepileptus*	?	?	V3	V11	Prootic and parietal
*Rena dulcis*	?	?	V5	V10	Prootic-otoocipital suture or Prootic, dorsal to proximal epyphisis of quadrate
*Rena humilis*	?	?	?	?	Prootic-supraoccipital suture and otoocipital
*Rena segrega*	V1	?	V6	V12	Prootic, dorsal to proximal epyphisis of quadrate
*Rena unguirostris*	V1	?	?	V6	Prootic-otoocipital suture
*Siagonodon cupinensis*	?	?	?	?	Prootic, dorsal to proximal epyphisis of quadrate
*Tetracheilostoma bilineatum*	?	?	?	?	Prootic, dorsal to proximal epyphisis of quadrate
*Trilepida brasiliensis*	V1	?	V3	V7	Prootic, dorsal to proximal epyphisis of quadrate
*Trilepida dimidiata*	V1	?	V5	V13	Prootic, dorsal to proximal epyphisis of quadrate
*Trilepida fuliginosa*	V2	?	V4	V7	Prootic, anterior to prootic-otoocipital suture
*Trilepida jani*	V1	?	Absent	Absent	?
*Trilepida joshuai*	V1	?	V2	?	Prootic, anterior to prootic–otoocipital suture
*Trilepida koppesi*	V1	?	V5	V13	Prootic, dorsal to proximal epyphisis of quadrate
*Trilepida macrolepis*	V1	?	?	?	Prootic-otooccipital suture and prootic-supraoccipital suture
*Tril*. *salgueiroi*	V1	?	V4	V13	Prootic-otooccipital and prootic-supraoccipital suture

#### *Musculus longissimus capitis*, *pars transversalis cervicis* (Fig 12; Table 7)

This muscle is located ventrally to the subcutaneous muscles, extending along the lateroventral region of the body, also contributing to the lateral protection of the cervical vertebrae. In lateral view, it lies ventrally to the *M*. *longissumus capitis*, *pars transversalis capitis*, contributing to the lateral cover of the (posterior) skull and neck. This muscle originates from the neural arches of the atlas and axis, and in the synapophyses of the vertebrae posterior to them. In *Rena* spp. (except for *R*. *unguirostris*) and *Trilepida* spp., a wide portion of fibers insert onto the skull dorsoposteriorly to the *M*. *longissimus capitis*, *pars transversalis cervicis*, and might represent a dorsal portion of this muscle. The dorsal portion is absent in *R*. *unguirostris*, *Epictia* spp., *M*. *lepitepileptus*, *Te*. *bilineatum* and *S*. *cupinensis*. Its anteriormost limit in Epictinae lies in the region between V1 and V5 and the posterior limit between V7 and V28 (Table 7). This muscle inserts via a fusiform tendon onto the lateroposterior region of the basioccipital.

**Table 7:** Variability on the area of origin and insertion of the *M*. *longissimus capitis*, *pars transversalis cervicis* for members of Subfamily Epictinae. Abbreviations: 1 = anterior limit of origin; 2 = posterior limit of origin; 3 = dorsal portion; “V” represents vertebrae of origin where V1 = atlas, V2 = axis, V3 = first thoracolombar vertebra and so on.

Species	1	2	3
*Epictia ater*	V3	V7	Absent
*Epictia tenella*	V1	V8 or V7	Absent
*Epictia phenops*	V2	?	Absent
*Mitophis lepitepileptus*	V2	V7	Absent
*Rena dulcis*	V4	V11	Present
*Rena humilis*	V5	?	Present
*Rena segrega*	V2	V9	Present
*Rena unguirostris*	V3	V8	Absent
*Siagonodon cupinensis*	?	V10	Absent
*Tetracheilostoma bilineatum*	V2	V7	Absent
*Trilepida brasiliensis*	V2	?	Present
*Trilepida dimidiata*	V2	V13	Present
*Trilepida koppesi*	V3	?	Present
*Trilepida fuliginosa*	V2	?	Present
*Trilepida jani*	V1	V5	Present
*Trilepida joshuai*	V2	?	Present
*Trilepida macrolepis*	?	?	Present
*Trilepida salgueiroi*	V2	V28	Present

#### *Musculus obliquus capitis magnus* (Fig 13; S1 Table)

This is a neck muscle of the *M*. *transversospinalis* group and the most medial and ventral element of the epaxial muscles. It is completely covered by the *M*. *spinalis et semispinalis capitis* and *M*. *longissimus capitis*, *pars transversalis capitis*. The *M*. *obliquus capitis magnus* also provides coverage to the dorsoposterior area of the skull, and a dorsal cover to the cervical and anterior thoracolumbar vertebrae. It originates from the dorsal surface of the atlas, axis and first thoracolumbar vertebra (except for *S*. *cupinensis*; but see variation in S1 Table). It inserts onto the prootic-supraoccipital-otooccipital suture or exclusively onto one of these elements. Interspecific variations are provided in S1 Table.

**Fig 13:**
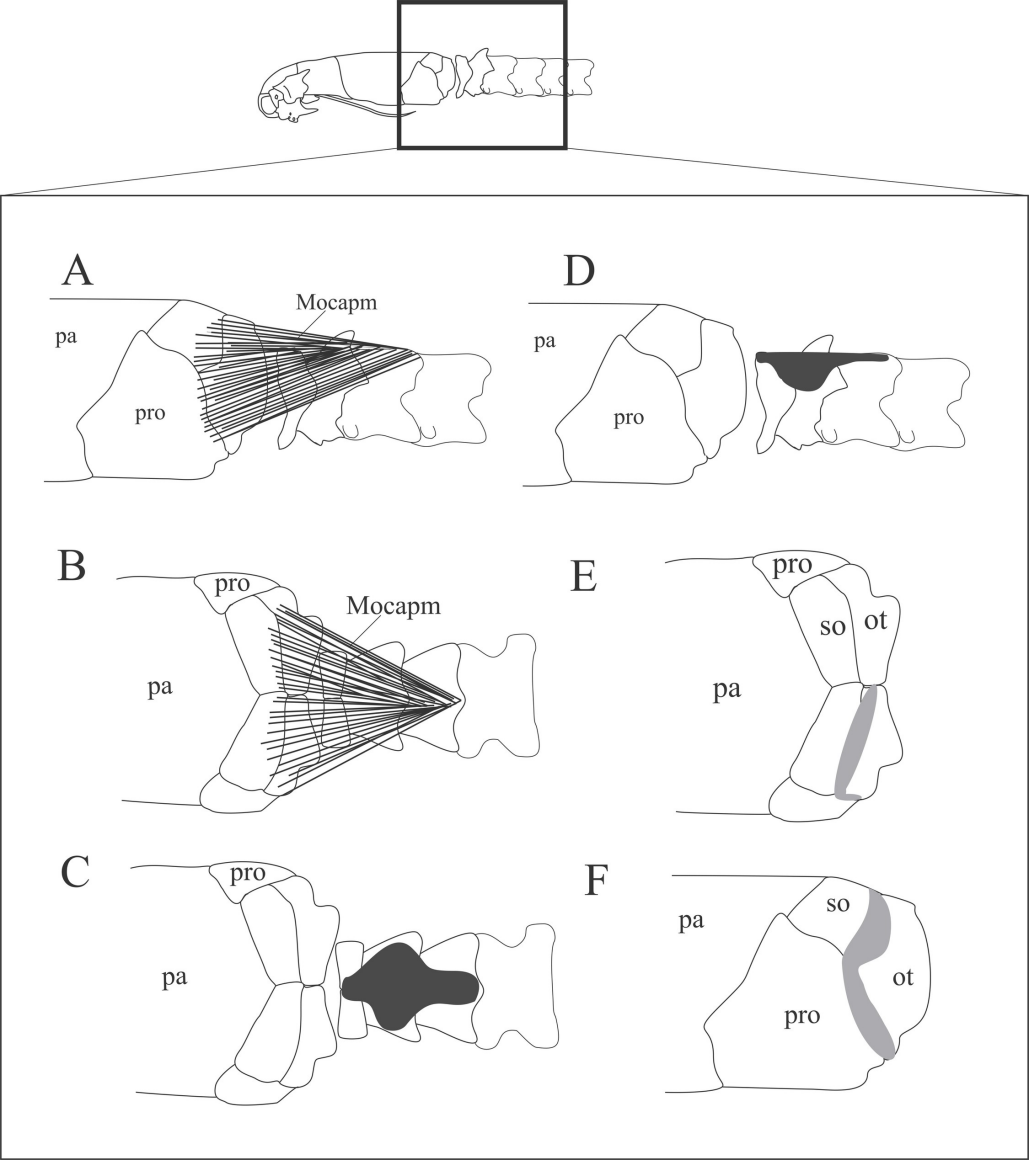
Schematic view of the location (A,B), origin (C,D), and insertion (E,F) of the *M*. *obliquus capitis magnus*. Dorsal (B,C,E) and lateral (A,D,F) views of the head. Abbreviations: Mocapm = *M*. *obliquus capitis magnus*, pa = parietal, pro = prootic, so = supraoccipital, ot = otooccipital.

#### *Musculus rectus capitis anterior*, *pars ventralis* (Fig 14; Table 8)

**Fig 14:**
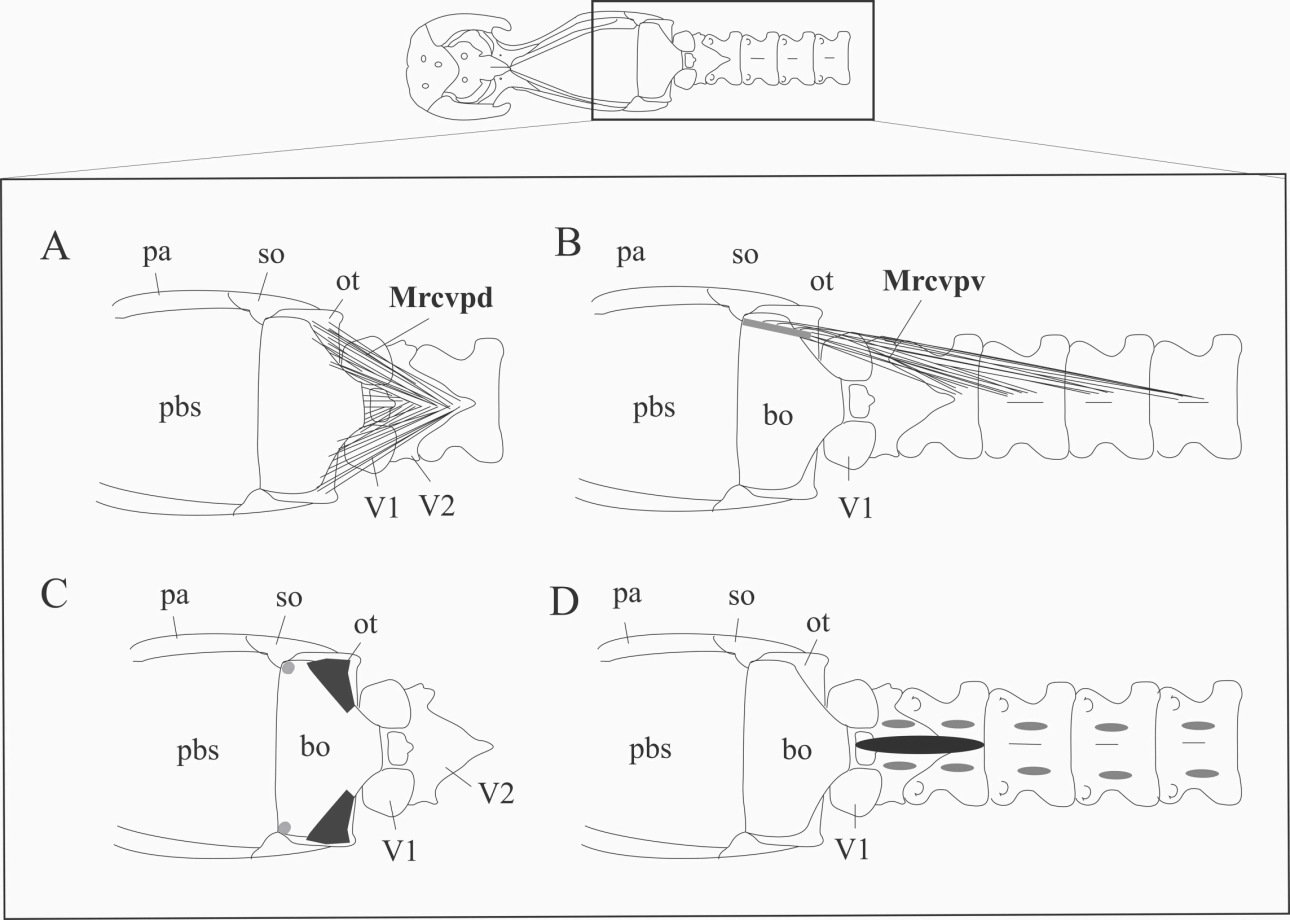
Schematic view of the head in ventral view with illustration of the location (A, B) origin (D) and insertion of the *M*. *rectus capitis anterior*, *pars ventralis* and *M*. *rectus capitis anterior pars dorsalis*. Black: *M*. *rectus capitis anterior*, *pars dorsalis*; Light gray: *M*. *rectus capitis anterior*, *pars ventralis*. Abbreviations: bo = basioccipital, Mrcvpd = *M*. *rectus capitis anterior*, *pars dorsalis*, Mrcvpv = *M*. *rectus capitis anterior*, *pars ventralis*, ot = otooccipital, pa = parietal, pbs = parabasisphenoid, pro = prootic, so = supraoccipital, V1 = atlas, V2 = axis.

The *M*. *rectus capitis anterior*, *pars ventralis* is a hypaxial muscle, located ventrally to the vertebrae, covering the *M*. *rectus capitis anterior*, *pars dorsalis* anteriorly (Fig 14B). It originates from the midline of the vertebrae’s ventral area (Fig 14D) and lateral to the intercentra II and II. The origins vary from V1 to V4 anteriorly and from V7 to V40 posteriorly (Table 8). It usually inserts via a fusiform tendon onto the posterolateral area of the basioccipital at the suture with the parabasisphenoid or exclusively onto the anterolateral area of the basioccipital (*Epictia ater*).

**Table 8:** Variability in the area of origin and insertion of the *M*. *rectus capitis anterior*, *pars ventralis* for members of Subfamily Epictinae. Abbreviations: 1 = anterior origin limit; 2 = posterior origin limit; 3 = posterior limit of extension of the tendon of insertion; 4 = insertion area in the basioccipital; AL = anterolateral face; BPS = basioccipital-parapasisphenoid suture; “V” + vertebrae of origin where V1 = atlas, V2 = axis, V3 = first thoracolombar vertebra and so on.

Species	1	2	3	4
*Epictia ater*	V3	V13	Posterior limit of basioccipital	AL
*Epictia tenella*	V4	V13	Posterior limit of basioccipital	BPS
*Epictia phenops*	V2	V12	Posterior limit of basioccipital	BPS
*Mitophis lepitepileptus*	V1	V7	V3	BPS
*Rena dulcis*	V3	V12	Posterior limit of basioccipital	BPS
*Rena humilis*	V2	?	Posterior limit of basioccipital	BPS
*Rena segrega*	V2	V18	Posterior limit of skull	BPS
*Rena unguirostris*	V3	V40	Posterior limit of skull	BPS
*Siagonodon cupinensis*	V3? V2?	V20	Half the basioccipital extension	BPS
*Tetracheilostoma bilineatum*	V2	V11	?	BPS
*Trilepida brasiliensis*	V2	V25	?	BPS
*Trilepida dimidiata*	V3	V22	?	BPS
*Trilepida koppesi*	V2	V27	?	BPS
*Trilepida fuliginosa*	V4	V17	?	BPS
*Trilepida jani*	V3	V22	?	BPS
*Trilepida joshuai*	V2	V28	?	BPS
*Trilepida macrolepis*	V2	V28	?	BPS
*Trilepida salgueiroi*	V2	V16	?	BPS

#### *Musculus rectus capitis anterior*, *pars dorsalis* (Fig 14)

The *Musculus rectus capitis*, *pars dorsalis* contributes to the deep neck muscles, being dorsal to *M*. *rectus capitis anterior*, *pars ventralis*. This muscle covers the posteroventral region of the skull from the basioccipital to the axis. It originates from the ventral face of the axis and laterally from the intercentra I and II. At the atlas, it arises from a narrow medial area of the intercentrum I and its articular facets. It might also arise from V3 in *E*. *tenella*, *E*. *phenops*, *Tr*. *joshuai* and *Tr*. *macrolepis*. This muscle inserts onto the basioccipital and otoccipital in areas adjacent to suture of both bones. (*E*. *ater*, *M*. *lepitepileptus*) or exclusively onto the ventral face of the otooccipital (*E*. *tenella*, *Rena spp*., *S*. *cupinensis*, *Te*. *bilineatum*, *Trilepida spp*; Fig 14D).

## Discussion

### Head muscles

Studies on the snake’s cephalic muscles have historically focused on anatomical descriptions, muscle bundles homologies between “lizards” and snakes [23] or even between supraspecific taxons amongst snakes (e.g., ‘Scolecophidia’ versus Aletinophidia) [43]. The drastic reconstruction of the jaw muscles from the lizard to ophidian condition have led to several losses and fusions of bundles present in “lizards” [23], and such modifications obscure the interpretation of several remaining muscular bundles in snakes [22]. The aponeurotic system and the innervation pattern of the snake’s jaw adductor muscles have allowed the proposition of several muscle homologies between “lizards” and snakes. However, “Scolecophidians” exhibit highly speciliazed phenotypes that lack an aponeurotic system in their jaw adductor muscles. Such phenotypes have for long obscured the homologies of the jaw adductor muscles between ‘Scolecophidians’ and “lizards” or even other snakes.

Previous studies on the the alethinophidian head muscles suggested two basic types of conformation for the jaw adductor muscles: “*levator anguli oris*” and the *“three externii*” [21,40]. Except for Typhlopoidea, three portions of the external adductor muscles are present in all snakes [13], including Leptotyphlopidae ([13,21,40], present study). The anterior portion of the adductor musculature consists of the *M*. *levator anguli oris* and the *M*. *adductor mandibulae externus superficialis* [23]. Both bundles are historically controversial regarding their interpretations by both anatomists and systematists [23], which is especially true for ‘Scolecophidians’. In contrast to the reduced *M*. *levator anguli oris* of Alethinophidia [21,39,40,60,61], the well-developed *M*. *levator anguli oris* in ‘Scolecophidia’ has traditionally been associated with an origin from the frontal/parietal and an insertion onto the mandible. However, some authors consider the superficial fibers of the *M*. *levator anguli oris* of threadnakes that insert onto the rictal plate as homologous the *M*. *levator anguli* oris of “lizards” as based on topographical homology hypothesis [23]. However, other authors [15,62,63] consider the superficialmost adductor element in snakes to correspond to the *M*. *levator anguli oris* of “lizards”. In “lizards”, the most superficial muscle that is referred to as *M*. *levator anguli oris* originates from the lateral extremity of the temporal fenestra, extends ventrally, and inserts onto the rictal plate [22,62]. In such a view, modifications might have occurred in order to accommodate the cephalic glands so that the *M*. *levator anguli oris* would insert totally or partially onto the venom gland or the mandible. The latter assumption was followed by several authors for Leptotyphlopidae [21,39,40,60], with the most superficial adductor fiberes inserting onto the mandible, what would correspond to the *M*. *levator anguli oris* in “lizards”.

The results gathered herein might suggest a distinct terminology from those nomenclature employed in previous studies [21,39,40,60,61]. Although the most superficial portion of fibers that lie medial to the *M*. *costocutaneus* is traditionally associated with a *M*. *levator anguli oris* [38], only the portion located anterior to the trigeminal nerve (that emerges superficially) inserts onto the lateral face of the dorsoposterior process of dentary. The superficial fibers, that are posterior to the anteroventrally descending trigeminal nerve, insert onto the posterior tip of the dorsal process of the coronoid and represent, very likely, an anterior portion of the *M*. *adductor externus medialis* (portion A; MAEMA). Such an assumption would restrict the *M*. *levator anguli oris* to a short muscle with an origin exclusively from the frontal or the frontal plus a small anterior area of the parietal (although that area is still wider than in other Alethinophidia [21,23]). Regardless of the terminology adopted for the “posterior portion” of the *M*. *levator anguli oris*, the homology of this muscle between “lizards” and snakes, as well as the *M*. *adductor externus superficialis*, is quite disputed in the literature, and must be addressed elsewhere in extensive and focused studies that include “lizards” and aletinophidian species.

Johnston [60] hypothesized the dorsoventral fibers posterior to the orbit, which were previously associated to an anteriormost portion of the *M*. *adductor externus superficialis* [21,40], as an anterior portion of the *M*. *levator anguli oris* (= *M*. *levator anguli oris* 1). In such a scenario, the *M*. *levator anguli oris* would insert both onto the rictal plate (same as “lizards”) and onto the dorsoposterior process of dentary. However, if we consider Zaher’s [19] proposal that in Alethinophidia the *M*. *levator anguli oris* represents the muscle that inserts onto the rictal plate, then the muscle herein identified as the *M*. *adductor externus superficialis* (both its anterior and posterior portions) might also represent the *M*. *levator anguli oris* located more medially. In such a scenario, the components of the *M*. *adductor externus medialis* would be divided into portions located more anteriorly, overlapping the hypothetical *M*. *levator anguli oris*. Thus, the MAEMA might represent a *M*. *adductor externus superficialis* considering it originates from the temporal region and inserts onto the coronoid in “lizards” [44]. In view of the hypothesis of the presence of a medial *M*. *levator anguli oris* (composed of both portions of the *M*. *adductor externus superficialis* of the present study) and a *M*. *adductor externus superficialis* lateral and posterior (= MAEMA in the present study), once again the muscle associated to *M*. *levator anguli oris* would represent an issue on the establishment of primary homology hypotheses in relations to “lizards” and snakes. A medial location of these superficial elements is reported in the Anomalepididae [41,42] and might also occur in some Leptotyphlopidae (species herein examined) as a result of their extreme miniaturization and/or due to its fossorial habits. In case the fibers posterior to the trigeminal nerve (herein *M*. *adductor externus medialis* portion A; MAEMA) represent in fact a posterior portion of the *M*. *levator anguli oris* (as suggested by previous authors), one should consider that the conditions found in the *M*. *levator anguli oris* in Leptotyphlopidae would then be apomorphic amongst snakes, with an insertion onto the coronoid bone. Even though such possibililty is very unlikely, future studies must address nerve pathways to refine homology hypothesis of the posterior fibers of the MAEMA in relation to the *M*. *levator anguli oris* of “lizards”. According to the results gathered herein, a well-developed *M*. *levator anguli oris* is limited to the subtribes Renina and Epictina and is reduced in Tetracheilostomina species. Unfortunately, comparisons with previous studies [13,14, 21,37] are not likely to be accurate considering the limited description of a few muscles, as well as for the difficulty on identifying the *M*. *levator anguli oris* solely based on available published images. However, illustrations of *Rena maxima* [21,40] suggest that this species also has a wide *M*. *levator anguli oris* as found in the present study. In ‘Scolecophidia’, a *Musculus levator anguli oris* is exclusively present in Leptotyphlopidae, being absent in Typhlopidae [21,42] or rudimentary in Anomalepididae [21,42].

In Epictinae, as well as in Anomalepididae and Typhlopidae [17,21,42], three components of the *adductores medialis* are present: *superficialis*, *medialis* and *profundus*. The *Musculus adductor externus superficialis* originates from the dorsolateral face of the parietal and converges anterodorsally toward the wide rictal plate in a typical arrangement for Leptotyphlopidae [13,21,40]. In Anomalepididae, this muscle originates from the postorbital region and descends to its insertion onto the coronoid, while in Typhlopidae there is a unique structural rearrangement amongst ‘Scolecophidia’, with the muscle being medial to the *adductor medialis* muscles [17,21].

The elongation of the quadrate anteriorly associated with a shortening of the mandible resulted in several rearrangements and modifications of the adductor muscles of Leptotyphlopidae in comparison to other snakes ([21], present study]. An example of such reorganization is evidenced in the *M*. *adductor mandibulae externus medialis* muscles in ‘Scolecophidia’, where a shortening of the ‘pre-coronoid’ area (and also the mouth) resulted in a shorter area of insertion onto the coronoid; as well as an anterior displacement of the *M*. *adductor medialis superficialis* and a posterior displacement of the *M*. *adductor externus profundus* ([21], present study).

In the specimens analyzed herein, the *M*. *adductor mandibulae externus medialis* is usually composed of three elements, with the posteriormost portion originating from the quadrate and the other two from the lateroposterior region of the parietal ([21,40] present study). However, the portion A (MAEMA) seems to be absent in *Siagonodon* and *Mitophis*, suggesting possible intergeneric variability in Epictinae. All parts converge anterolaterally and insert onto distinct regions of the coronoid, in a similar pattern as found in Typhlopidae (= *M*. *aduc*. *med*. *ext*. *medius sensu* Iordansky [17]). The *Musculus adductor externus medialis* A (MAEMA; present study) originates from and inserts onto the same areas in Typhlopidae, while the portion “B” (MAEMB) seems to be homologous to the *M*. *adductor externus medialis medius* of Typhlopidae, and portion C seems to be exclusively present in Leptotyphlopidae (present study); considering portion C is absent in Anomalepididae or Typhlopidae [21,41,42]. A different pattern of the *adductor medialis* is described in Anomalepididae with the portions located medially to the Harderian gland [21,41, 42], in contrast to the pattern found in typhlopids [17,21] and leptotyphlopids (present study).

In Leptotyphlopidae, the *M*. *adductor medialis profundus* is bipennate, originates from the quadrate, and inserts *via* a tendon onto the supracotylar process of the compound bone. Previous studies [21,40] report that this muscle arises from the dorsolateral region of the quadrate and inserts onto the posterolateral area of the mandible, anterior to the quadrato-mandibular joint, in a plesiomorphic condition for snakes [21]. Our data of the *M*. *adductor medialis profundus* shows that its possible origin is from the dorsal, lateral or ventral face of the quadrate via a tendon associated to the posterior region of this bone. Additionally, its insertion is via a tendon onto the supracotylar process of the compound bone.

The muscle herein tentatively assigned as *M*. *adductor mandibulae posterior* is located medially to the quadrate and might represent a ventromedial portion of the *M*. *adductor externus medialis* C (MAEMC), considering both muscles are usually indistinct. However, a medial insertion onto the prearticular and supracotilar medial face might indicate a distinct *M*. *adductor mandibulae posterior* itself. The difficulties of differentiating this muscle are due to an anterior location of the mandibular ramus of the trigeminal nerve, as found in *Lanthanotus* and several basal snakes [19,21]. A more refined study that includes the analysis of nerve pathways (mostly on the trigeminal nerve) might contribute to the corroboration if this portion is (i) a distinct *M*. *adductor mandibulae posterior* (as suggested in this study); (ii) a medial portion of the *M*. *adductor externus medialis* C (MAEMC) with fibers not inserting onto the coronoid, and thus the medial adductors would be composed of two portions originating exclusively from the skull; or (iii) if such element represents a medial portion of the *M*. *adductor profundus*. In Typhlopidae, the wide and peculiar muscle traditionally associated to the *M*. *retractor maxillae* has previously been suggested to be homologous to the *M*. *adductor mandibulae posterior* of “lizards” based on its innervation pattern [62]. On the other hand, the presence of a second nerve emerging from the mandibular branch of the trigeminal nerve obscures its clear homology statement [21]. Such a muscle has not been reported for Anomalepididae [21,41,42] and histological sections of *Anomalepis* and *Liotyphlops* did not reveal the presence of any muscle topologically equivalent to the *M*. *adductor mandibulae posterior* in Anomalepididae (see [41,42]). According to Haas [21], the variability observed in the presence/absence of the *M*. *adductor mandibulae posterior* as suggested by Lakjer [62] does not seem to reveal any valuable systematic information for snakes. In fact, the interspecific variation reported herein to Epictinae suggest those muscles were lost in some species of *Rena* (*R*. *unguirostris*, *R*. *segrega*) and *Trilepida* (*Tr*. *brasiliensis*, *Tr*. *dimidiata*, *Tr*. *jani*, *Tr*. *Macrolepis*, *Tr*. *salgueiroi*) reinforces such interpretation.

The presence of a single *M*. *pseudotemporalis* is characteristic of the snakes [19,21], which corresponds topographically to the *M*. *pseudotemporalis profundus* of “lizards” [21,62]. In Epictinae, this muscle is very similar to that of other ‘Scolecophidia’, although in the latter its origin does not extend anteriorly to include the lateral part of the frontal (present study). The reduced variation in the origin of that muscle is also reported in Alethinophidia [19,21].

As in other Leptotyphlopidae, the *M*. *levator pterygoidei* is absent in Epictinae [17,21]. In Typhlopidae, this muscle is present but highly modified and possibly serves the function of a well developed *M*. *retractor maxillae*. In Anomalepididae, the *M*. *levator pterygoidei* is present in a similar pattern as found in Alethinophidia [21,41,42].

The presence of a double *M*. *pterygoideus* (main and accessory portions) represents a typical characteristic amongst snakes [19,21], although the pattern found for Leptotyphlopidae ([21], present study) is more similar to Typhlopidae rather than Anomalepididae. In Leptotyphlopidae ([21], present study), the *M*. *pterygoideus* emerges from the posterior process of the maxilla, extends posteroventrally and inserts onto the short retroarticular process of the compound bone. In Leptotyphlopidae, the presence of this muscle reinforces the hypothesis that the process that develops posteriorly in the articular bone is in fact a true retroarticular process. According to McDowell [64], the presence of a well-developed palatal bundle of muscles in Leptotyphlopidae, with a relatively wide *M*. *pterygoideus*, might be associated to some snout complex mobility, as previously described for some Typhlopidae species [44]. However, such hypothesis seems very unlikely considering the extreme ossification of the palate in Leptotyphlopidae with no cartilaginous or fibrous connection triggering the flexion of the snout complex. In Typhlopidae, two portions of the *M*. *pterygoideus* are present, the main one inserting onto the retroarticular process with its origin from the maxilla, and the *accessorius* originating from the lateral surface of the pterygoid [17,21], like the pattern observed in Leptotyphlopidae (present study). In Anomalepididae, there is a single *M*. *pterygoideus* with its origin from the prefrontal (not from maxilla), displaying a unique conformation amongst snakes with the prefrontal participating in the maxillary movement [41,42]. The presence of a well-developed *M*. *pterygoideus* in Leptotyphlopidae is associated with a static maxilla may be related to the mandibular movements, allowing the maxilla to anchor the muscle to functionally act as a protractor of the mandible, performing a distinct function in Anomalepididae (present study). Based on our results, the *M*. *pterygoideus acessorius* may be divided into two portions (here called *M*. *pterygoideus acessorius anterior* and *posterior*), which might be distinct by a conspicuous gap. We hypothesize that the anterior elongation of the quadrate may have allowed the rearrangement of this muscle inserting onto its anteromecial face, element which is topographically equivalent to the compound bone of the Alethinophidian snakes (where this muscle inserts), with the *M*. *pterygoideus acessorius anterior* assuming the functional action of a *M*. *protractor quadrati* from Alethinophidia [19,21].

The muscle herein referred as “Unnamed Muscle 1” is exclusively present in *Rena humilis* (n = 1; 50%) and *Epictia tenella* (n = 1; 50%). We could not establish an association with any muscle previously described for snakes, thus, its presence demands further investigation on its primary homology statements and putative function. This muscle seems to be associated in some level with moving the glottis anteriorly and laterally (lateral expansion) for prey gasping during feeding.

The *Musculus protractor pterygoidei* is similar to other snakes [17,19,21,41, 42], with reduced interespecific variability in Epictinae. Its general pattern is more like Typhlopidae where it covers the posterior half of the pterygoid [17], although in Leptotyphlopidae it covers the pterygoid entirely or almost totally. In Typhlopidae, the *M*. *protractor pterygoidei* elevates the maxilla [17] and thus is important in feeding, while in Anomalepididae it is fused to the *M*. *levator pterygoidei* enabling the pterygoid protraction, but moving the maxilla [41,42]. In leptotyphlopids, the *M*. *protractor pterygoidei* inserts onto the pterygoid, which connects anteriorly to the palatine, the latter is associated with a static prefrontal and maxilla [65]. We speculate that the palatine provides an anterior support to anchor the posterior edge of the pterygoid moving through the contraction of the *M*. *protractor pterygoidei*, thus elevating its posterior region and acting as a *M*. *levator pterygoidei*. The elevation of the pterygoid might also act in combination with the *M*. *pterygoideus acessorius* protracting the mandible.

The *Musculus retractor pterygoidei* exhibits low interespecific variation, and the morphology of this muscle is relatively conserved amongst snakes, with a few variations of its insertion area, which might also occur in the dermis as in Anomalepididae, Typhloipdae and Viperidae ([21,41,42] = *M*. *retractor palatinae sensu* Iordansky [17]). This muscle is considered homologous to the *M*. *retractor bulbi ventralis* of “lizard” (see [17]), and its presence in a simplified conformation is important to understand the evolution of Squamates [21]. The apparent atrophy of the *M*. *retractor bulbi ventralis* followed by its rearrangement would corroborate the hypothesis of snake’s evolution from an ancestor with a well-developed brillar cavity and reduced optic apparatus, followed by a rearrangement of the *M*. *retractor pterygoidei*. Therefore, corroborating a scenario for a fossorial origin of snakes [21].

In vertebrates, six extrinsic eye muscles are usually present: four *rectus* muscles and two *obliquus* [66,67]. In Epictinae, two *obliquus* muscles and three *rectus* muscles are found (present study), and, as in all snakes, the *Musculus bursalis*, *M*. *retractor bulbi* and *M*. *depressor palpebralis inferior* are absent [67]. The absence of the oblique eye muscles has been previously considered a shared characteristic of ‘Scolecophidia’, since the previous studies did not report such muscles [39,41,42,61]. Thus, the present study is the first to report both *obliquus* and *rectus* muscles in ‘Scolecophidia’. In general, the *obliquus* muscles are the most common in Epictinae, and if functional, would perform the dorsoventral rotation of the eye. However, even if its insertion suggests this ocular movement, the muscles are extremely thin and appear to be too rudimentary to perform any movement. Due to their fragile nature, the muscles are easily damaged during dissection and its apparent absence in *Epictia ater*, *Rena unguirosris* and *Trilepida dimidiata* demands further investigation in this respect. In addition to the dissection of specimens, histological studies are necessary to confirm specific origins of the *rectus* muscles that were damaged during dissection. According to McDowell ([64], based on [41,42]) extrinsic eye muscles are absent in Anomalepididae, while Haas [13] reports the presence of rudimentary muscles associated to the optic nerve (possible from the *rectus* group according to our re-interpretation) in Typhlopidae.

The hypobranchial muscles associated with the hyoid, as well as the ventral constrictors, exhibited low inter- and intraspecific variation in Epictinae, in a similar pattern previously described in literature [12,21,43]. The variability of such elements amongst ‘Scolecophidia’ is also reduced in comparison to the dorsal and lateral head muscles (*adductores mandibulae* and *superficialis*), as will be discussed below. In Epictinae, the most remarkable variations found herein are associated with the insertion of the “Unnamed Muscle 2” and with the *M*. *geniotrachealis*, as well as the general pattern of the *M*. *cervicomandibularis*. Despite the inter- and intraspecific variation herein reported to all Epictinae, the “Unnamed Muscle 2” inserts onto the glottal tube. We could not identify name the aforementioned muscle, even if Langebartel [43] and Groombridge [12] have previously illustrated it.

The *M*. *geniotrachealis* varied inter- and intraspecifically in relation to its insertion onto the trachea. In a similar pattern as the “Unnamed muscle 2”, this muscle seems to be associated with the medial or posterior region of the glottal tube and might include tracheal rings itself. Its morphology is similar to that in Typhlopidae and Anomalepididae, as well as Alethinophidia [43]. Despite the *M*. *cervicomandibularis* showing some degree of interspecific variation with respect to its insertion, type (simple or double), and tendon arrangement (unipennate or bipennate), Langebartel [43] considers this muscle as a *M*. *neuromadibularis* based on the innervation pattern, in a way that the *M*. *cervicomandibularis* (when present), would be associated with the former. Groombridge [12] considered the *M*. *neuromandibularis* as absent in ‘Scolecophidia’, recognizing this element as a *M*. *cervicomandibularis* (terminology followed herein). Despite the distinct terminologies, some differences in the insertion of this muscle might be mentioned according to Langebartel’s result, who described two typical patterns of insertion in snakes: the first–found in ‘Scolecophidia’, Uropeltidae and Aniliidae–is characterized by a tendon inserting onto the mandible, without the association with the “*ceratomandibularis*”, as also found herein for Epictinae (even if some variation were found with respect to its insertion). Anomalepididae shares this pattern of the *M*. *neuromandibularis* (herein nominated as *M*. *cervicomandibularis*) not being associated with the “*M*. *cervicomandibularis”*, while typhlopids exhibit a “*M*. *cervicomandibularis*” without association with the “*M*. *neuromandibularis*”. Although the presence of two portions in Typhlopidae demands further investigations regarding its homology, the single element described for Leptotyphlopidae (and also Anomalepididae) [43] was not found in Epictinae. In a few of the species examined for the subfamily Epictinae, this muscle is double (*E*. *tenella*, *E*. *phenops*, *R*. *dulcis*), possibly resembling the Typhlopidae pattern. However, as we were not able to analyze the innervation pattern of these muscles, we preferred to follow Groombridge [12] in considering a *M*. *neuromandibularis* absent and, consequently, the presence of a *M*. *cervicomanbidularis* divided into two portions, even though one of those portions might represent a *M*. *neuromandibularis* as suggested by Langebartel [43]. The *M*. *cervicomandibularis* is bifid and inserts via tendon onto the dentary in *Tetracheilostoma bilineatum*, while in *Trilepida* spp. (except *Tr*. *macrolepis* and *Tr*. *salgueiroi*) it shares the same insertion tendon with the *M*. *ceratomandibularis*. This pattern is not found in other ‘Scolecophidia’ and apparently not in any Alethinophidia, being reported herein for the first time.

The *Musculus costocutaneus superior* is extremely adhered to the subcutaneous muscles, which makes its dissection/observation difficult ([43], present study). The two specimens dissected herein were similar to *Rena maxima* [43], i.e., the muscle is not attaching to the hyoid as reported for Typhlopidae [43]. This condition is distinct from Anomalepididae with the *M*. *costocutaneus superior* inserting onto the hyoid (located more anteriorly).

The *M*. *depressor mandibulae*, *M*. *genioglossus* and *M*. *geniomucosalis* show reduced variability when compared to other ‘Scolecophidia’ [12,43]. However, a well-developed *M*. *genioglossus* is characteristic of Leptotyphlopidae [12] possibly acting as the primary retractor of the mandible during feeding [50]. We follow Langerbatel [43] in naming such muscle as *M*. *genioglossus* instead of *M*. *geniohyoideus* as the author limits the term “geniohyoideus” exclusively to the family Anomalepididae, and the muscle that originates in the mandible and inserts onto the hyoid as “genioglossus”. According to such author, the geniohyoideus is commonly found in “lizards”, where it exists as the deep layer of the ramus-hyoid series, running from the mandible to the ceratohyal. In such a panorama, the geniohyoideus is considered absent in all snakes except the anomalepidids, where it is a broad muscle extending from the ramus to the hypohyal plus ceratohyal [43]. Even if there are a few homology issues regarding this muscle as well as other head muscles, homology problems will be addressed elsewere in a future detailed study (in prep) that including both “lizard” and snake taxa. The *Musculus geniomucosalis* apparently occurs exclusively in ‘Scolecophidians’ [12]. The difficulty on stablishing homologies have lead Groombridge [12] to propose two hypotheses: (i) if the *M*. *geniomucosalis* derives from the *M*. *geniotrachealis* (unique muscle in snakes), then this muscle would represent a synapomorphy of ‘Scolecophidia’; or (ii) if the *M*. *geniomucosalis* derives from a lateral portion of the *M*. *genioglossus* of “lizards” (which also inserts onto the mucosa in the specimens herein analyzed), then this character would be a symplesiomorphy of ‘Scolecophidia’, while Alethinophidia would present a derived state of the character (absence). A second portion of the *M*. *geniomucosalis* is herein described and might be (i) only found in Epictinae, considering it was not mentioned for Leptotyphlopinae [12] or (ii) representing a *M*. *hyotrachealis* with fibers associated posteriorly to the *M*. *geniomucosalis*. Groombridge [12] reports that in some species the *M*. *geniomucosalis* and the *M*. *hyotrachealis* might be fused at their origins from the mandible. However, in the species analyzed herein, the medial portion of the *M*. *geniomucosalis* that inserts onto the trachea (possibly the *M*. *hyotrachealis*) does not originate from the mandible and apparently inserts at some point anterior to the larynx. Their fusion seemingly occurs more posteriorly near to its insertion onto the oral cavity, where its distinction is relatively difficult.

The *M*. *depressor mandibulae* in snakes is composed of two portions: the quadratic and the post-cranial portions [21]. In Epictinae (present study), as well as in other Leptotyphlopidae and *Anomalepis* [13,21,40], only the quadratic portion is present, which always is covered by the medial adductor muscles and by the *M*. *cervicomandibularis*. In Typhlopidae and *Liotyphlops*, this muscle has a distinct origin, with the quadratic portion absent and with the occipital portion well developed (Typhlopidae) or slightly less robust (*Liotyphlops*; [21]).

The *M*. *cervicoquadratus* is present in all snakes, with its origin from the ventrolateral region of the body, extending anteriorly and medially to the *M*. *cervicomandibularis* (when present; [33]). The homology of the *M*. *cervicoquadratus* in snakes and “lizards” has been discussed, and is traditionally associated with the *M*. *sphincter colli* [68] or the *M*. *costocutaneus superior* [21,69]. Notwithstanding, distinct terminologies have also been used for this muscle, such as a *M*. *cervicoquadratus* (e.g., [16,46,70]), *M*. *retractor ossi quadrati* (e.g., [67]) or *M*. *retractor quadrati* (e.g., [13,69]). Based on a detailed study aiming to establish the homology of *M*. *cervicoquadratus*, Tsuihiji et al. [33] consider it as a homologous component of the *M*. *episternocleidomastoideus* (component the *M*. *cuccularis* complex from “lizards”) and, consequently, such muscle might represent a remnant scapular muscle in snakes. The conclusion of Tsuihiji et al. [33] seems satisfactory, although additional developmental studies are necessary to corroborate the embryological origin of this muscle, as sugested by the authors themselves. In Typhlopidae, these muscles emerge from the subcutaneous muscles that overlay the dermis [33], in a similar pattern as found in the present study for Epictinae. Similar to Typhlopidae and *Acrochordus granulatus* [33], Epictinae species (present study) are an exception amongst snakes in having the *M*. *cervicoquadratus* origin from the lateral face of the prootic and not from the quadrate (except in *E*. *ater* and *E*. *tenella*, where the origin might also include the proximal head of the quadrate). However, a few differences between Typhlopidae and Leptotyphlopidae (Epictinae) are evident. In Typhlopidae, this muscle is medial to the *M*. *depressor mandibulae* [33], while in Epictinae it lies laterally to it. Additionally, a second portion of the *M*. *cervicoquadratus* (dorsal portion) is present in all Typhlopidae [33], while only *Rena dulcis* presented a dorsal element as found in Typhlopidae. As this muscle is extremely adhered to the subcutaneous muscles, it seems most likely that the dorsal portion (if present in all species) was lost during dissection for several taxa from Epictinae. Thus, the confirmation of a dorsal portion of the *M*. *cerviquadratus* (*sensu* [33]) in other Leptotyphlopidae demands further investigation, mainly through histological sections.

The three ventral constrictors (*M*. *intermandibularis anterior*, *M*. *intermandibularis posterior pars anterior and pars posterior*) have a similar topographical relation in ‘Scolecophidia’ [12,43]. The pattern of arrangement of these muscles (obliquely), as well as the extension of those muscles is distinct from Alethinophidia, and scolecophidian snakes seem to represent an ancestral state for Squamata [12]. The ventral constrictors also present difficulties with respect to their terminology [12,41–44]. According to Groombridge [12] the intermandibular portions referred to as IM1 and IM4 are exclusive present in Anomalepididae, and absent in Leptotyphlopidae, Typhlopidae and Alethinophidia. We report the presence of the *M*. *ceratomandibularis* with three portions, in a similar pattern as previously described for *Rena maxima* and Aniliidae [43]. However, the lateral (*Tr*. *jani*) and medial (*M*. *lepitepileptus*) portions might be lost in some species. If this character is not due to a dissection mistake, a double *M*. *ceratomandibularis* is first reported herein for Leptotyphlopidae. In Typhlopidae, this muscle is thin and exclusively composed of the lingual portion, while in Anomalepididae it is absent; even though its presence has been previously mentioned [43].

### Neck muscles

In Epictinae, the *M*. *spinalis capitis*–*M*. *spinalis et semispinalis capitis* complex–did not show any variation on its origin and insertion. An insertion occurring exclusively onto the parietal seems to be only found in Leptotyphlopidae and Anomalepididae, bearing in mind that in other taxa the insertion is onto the supraoccipital ([42,64], present study). According to McDowell [64] the absence of a muscle that moves the roof of the skull (with dermal origin) would be related to the absence of metakinesis in snakes, with a similar pattern found in “lizards”. According to Tsuihiji et al. [34], the *M*. *spinalis capitis* tends to be shorter in ‘Scolecophidia’ (*Anilios nigrescens*, *Afrotyphlops schelegeli* and *Argyrophis muelleri* in Typhlopidae and *Rena dulcis* in Leptotyphlopidae) when compared to Alethinophidia, and such assumption was corroborated herein. Additionally, both burrowing and aquatic snake species tend to have shorter spinalis muscle-tendon portions [36], and, therefore, the strict fossorial habitat of scolecophidian might be associated to their short spinalis muscle-tendon portion.

However, the area of insertion of the *M*. *semispinalis capitis* in Epictinae (present study) is usually broader than previously reported for Leptotyphlopidae ([34], posterior limit at V3 in *Rena dulcis*). Although Jayne [31] and Tsuihiji et al. [34] found a similar pattern for Typhlopidae (posterior limit at V2), we found that it varies both intra- and interspecifically in Epictinae. Similarly, the *M*. *semispinalis capitis* is also short as in Typhlopidae and basal lineages of Alethinophidia (i.e., Tropidophiidae, Boidae and Pythonidae; [34]). Thus, the posterior limits previously reported for ‘Scolecophidia’ (V6–V9; [34]) are herein expanded, and might occur from V4–V10, with the posteriormost limits usually occurring in *Trilepida* spp., which is similar to Aniliidae and Tropidophiidae [34]. Although variation on the extension of the *spinalis* muscles might be associated with the huge variation on vertebrae number in snakes, as a higher number of vertebrae is directly related to longer areas of insertion [36], species herein analysed with higher number of vertebrae did not exhibit wider areas of insertion (but see Table 5). Finally, due to the adaptive hypothesis that the evolution of habitat usage has strongly influenced the morphology of the spinalis muscle-tendon portion in snakes [36], such variation found in scolecophidians must be addressed in future studies, considering differences in habitat type and excavatorial methods.

Another muscle that is usually reduced in ‘Scolecophidia’–the *M*. *retractor capitis anterior*, *pars dorsalis–*was previously reported with a posterior limit at V11–V15 in ‘Scolecophidia’ [34], what is herein expanded and might occur from V7 (*M*. *lepitepileptus*) to V40 (*R*. *unguirostris*). Therefore, species of *Epictia*, *Tetracheilostoma* and *Rena* (except *R*. *unguirostris*) have a moderate extension of attachments (V11–V18) and a similar pattern as previously reported in ‘Scolecophidia’. On the other hand, *Trilepida* and *Siagonodon* have a posteriormost elongation of this muscle (V16–V27) in comparison to *Epictia*, in a similar pattern to that found in other Alethinophidia. However, *Rena unguirostris* exceeds the posterior limit (V40) previously known for snakes as a whole (i.e., V28 for Loxocemidae, *Trilepida* spp., present study). Considering both Tsuihiji et al. [34] and our results, the posterior extension of the *M*. *rectus capitis anterior*, *pars ventralis* is highly variable and does not seem to contain any phylogenetic signal. In agreement with this, a wider posterior limit seems to occur in some fossorial Alethinophidia with no close phylogenetic affinities (e.g., *Eryx jaculus*, *Rhinophis blytthii* and *Uropeltis melanogaster*), ‘Scolecophidia’ (present study) and Amphisbaena [34]. Consequently, such an elongation might reflect locomotor demands for a strictly fossorial lifestyle, contributing to an optimal excavation performance in certain microhabitats. On the other hand, the presence of an elongated *M*. *rectus capitis anterior*, *pars ventralis* in species with no fossorial habits (e.g., *Euneces murinus*; with semi-aquatic habitus) indicates that the selection for muscle elongation is not only associated to the occupation of fossorial niches. Even though the extreme posterior elongation of this muscle in *Rena unguirostris* demands additional investigation, such feature could be associated to a distinct locomotor function due to its shovel-shaped snout (present study). Such a speculation finds an evolutionary parallel in the case of *Rhineura floridana* (amphisbaenid with a shovel-shaped snout), which has a *M*. *rectus capitis anterior*, *pars ventralis* posterior attaching at V28. In fact, the area of insertion in *Rena unguirostris* represents the most extreme case known for Squamata and demands future studies on its functional morphology.

The *M*. *longissumis capitis*, *pars transversalis cervicis* is also long, and its posterior elongation is possibly associated with fossorial habits [34]. This muscle is interspecifically highly variable in Epictinae with its posterior limit occurring from V7 (*Epictia* spp., *Tetracheilostoma* spp., *M*. *lepitepileptus*) to V28 (*Tr*. *salgueiroi*). If Tsuihiji et al. [34] assumptions are correct, then one possible explanation for the variability found herein is that it might indicate different elongations due to distinct demands of excavation associated with soil properties. Such posterior elongation as a direct requirement for locomotion has previously been corroborated for the *spinalis* muscles and should be tested for the *longissimus* in the future.

Descriptive studies of the *M*. *longissimus capitis*, *pars transversalis capitis*, *M*. *obliqus capitis magnus* and *M*. *rectus capitis anterior*, *pars dorsalis* are very scarce for snakes in general. We found that the general pattern of these muscles for Epictinae are consistent with those based on general patterns reported for other snakes [32,71]. The *M*. *obliqus capitis magnus*, *pars dorsalis* and the *M*. *rectus capitis anterior*, *pars dorsalis* are robust portions of fibers possibly associated to the elevation and depression of the head and, therefore, essential for the excavation processes. Its low inter- and intraspecific variation leads us to conclude that such muscles are conserved in the subfamily. However, the *M*. *longissimus capitis*, *pars transversalis capitis*, responsible for the lateral angulation of the head and possible neck undulation movements varies interspecifically in relation to its posterior limit, which is hard to distinguish from other trunk muscles.

### The neck-trunk boundary in snakes

The reduction and/or loss of limbs evolved several times amongst Squamata and are usually associated with the posterior elongation of the body and the loss of axial regionalization [72]. Although the full loss of regionalization has been opposed by Head & Polly [73], the complete absence of a pectoral girdle in several groups hampers the establishment of the neck-trunk boundaries in snakes and how such regions evolved from an ancestor with limbs [34].

The neck-trunk boundaries (or even the presence of a neck) in snakes based on morphological evidences has previously been disputed in the literature, and three basic hypotheses have arisen: (i) body elongation in snakes is a result of extreme neck elongation [74, 75]; (ii) the cervical region is completely absent in snakes [4]; and (iii) a neck is present, but several anatomical structures usually associated to the neck-trunk boundary with limbs are dissociated in snakes [34,76]. The presence of vertebral hypapophyses in the body vertebra lead Nopsca [74] and Caldwell [75] to support the hypothesis i. The hypothesis ii is based on *HOX* gene expression limits (*HOXC8* and *C6*), frequently associated with the neck-trunk boundaries in Tetrapoda and that are present until the craniovertebral limit in snakes [72]. The hypothesis ii was refuted by Woltering et al. [77] and Head & Polly [73], who found a silent *HOX* gene expression pattern (i.e., does not manifest phenotypically) in the neck-trunk limits in snakes. According to Head & Polly [73], the view of a regionalization loss and silent expression was demonstrated by the occurrence of morphological regionalization in the body of vertebrae. The arguments of Cundall & Greene [4] to support hypothesis ii include both morphological aspects of the trunk and pleuroperitoneal cavity in snakes, which only extend until the craniovertebral limit. However, current morphological evidences give little support to hypotheses i and ii [31, 33, 34]. Hypothesis iii is based on both morphological [33,34,76] and molecular [73,77] independent evidences. Some morphological results gathered in this study also support the hypothesis iii. According to this hypothesis, a series of muscles might represent potential candidates to infer the neck-trunk boundaries in snakes. Among the muscle complexes that are traditionally more informative in relation to the neck-trunk boundaries, one might mention the *M*. *cervicoquadratus* [33], *M*. *spinalis capitis*, *M*. *semispinalis capitis* and *M*. *rectus capitis anterior* [34]. The dissociation of such muscular groups was also verified in the present study. The posterior limit of the *M*. *spinalis et semispinalis* in Epictinae (V7) is completely dissociated from the posterior limits of the *M*. *spinalis et semispinalis* (V28) in a similar pattern as found in Squamata with reduced or absent limbs [31, 32, 34] and thus the muscle is not providing informative data on the head-neck boundary. According to Tsuihiji et al. [33, 34], the *M*. *cervicoquadratus* (homologous to the *M*. *cucullaris*, *sensu* Tsuihiji et al. [33]) might represent a potential informative muscle for neck-trunk inference. Considering the above arguments, we found the complete loss of the neck in some Epictinae as supported by the posterior limit of the *M*. *cervicoquadratus* at the posterior limit of the skull (*E*. *ater*, *M*. *lepitepileptus*, *Te*. *bilineatum*), thus supporting hypothesis ii for some genera or species. On the other hand, hypothesis iii would represent a widespread and usual pattern for the remaniend taxa in the subfamily Epictinae analysed herein. By contrast, our results might also indicate that the *M*. *cerviquadratus* does not represent an ideal candidate for such inference and that possibly the *M*. *spinalis et semispinalis* or the *M*. *rectus anterior* perhaps may be considered in this respect.

## Supporting information

S1 Appendix Material examined. (DOCX)

S1 Table Synthesis of inter- and intraspecific variability of the head and neck muscles for members of the Subfamily Epictinae. Abbreviations are as follows: ? = Unknown, NV = Not variable, N/A = Not applicable, BV = Bilaterally variable. “V” = vertebrae of origin where V1 = atlas, V2 –axis, V3 –first thoracolombar vertebra and so on.
(DOCX)
