# Supplementary material for: Moving beyond the surface: Comparative head and neck myology of threadsnakes (Epictinae, Leptotyphlopidae, Serpentes), with comments on the ‘scolecophidian’ muscular system
Source: PLoS One. 2019 Jul 18;14(7):e0219661. doi: 10.1371/journal.pone.0219661 (PMC6638936; doi:10.1371/journal.pone.0219661)
Supplement: S1 Appendix — (DOCX) [file pone.0219661.s001.docx]

**Material examined**

*Epictia ater* (*n*=1): **EL SALVADOR**: SAN MIGUEL: KU18384.

*Epictia phenops* (*n*=1): **MEXICO**: OAXACA: FMNH 111477.

*Epictia tenella* (*n*=2): **BRAZIL**: PARÁ: *Oriximiná: Porto Trombetas*: MNRJ 16827. **TRINIDAD AND TOBAGO**: TRINIDAD: *Guayagueyare*: MCZ 60801.

*Mitophis lepitepileptus* (*n*=1): **HAITI**: SUD-EST: *Soliette*: USNM275548,

*Rena dulcis* (*n*=2): **UNITED STATES OF AMERICA**: OKLAHOMA: *Comanche*: OMNH35564, 35580.

*Rena humilis* (*n*=2): **UNITED STATES OF AMERICA**: CALIFORNIA: *San Diego*: FMNH 33950, 34302.^.^

*Rena segrega* (*n*=2): **UNITED STATES OF AMERICA**: NEW MEXICO: *Doña Ana*: LACM, 134007, 134009.

*Rena unguirostris* (*n*=1): **ARGENTINA**: TINOGASTA: *Palo Blanco*: FML 1773.

*Siagonodon cupinensis* (*n*=1): **BRAZIL**: MATO GROSSO: *Barra do Tapirapés: Guarantã do Norte*: UFMT 5627.

*Tetracheilostoma billineatum* (*n*=1): **MARTINIQUE**: LE LAMENTIN: *without locality*: USNM 564808.

*Trilepida brasiliensis* (*n*=1): **BRAZIL**: MATO GROSSO DO SUL: *Corumbá*: UFMT 1159.

*Trilepida dimidiata* (*n*=1): **BRAZIL**: RORAIMA: *Boa Vista*: MZUSP 10090.

*Trilepida fuliginosa* (*n*=1): **BRAZIL**: GOIÁS: *Luziânia*: CHUNB 40847.

*Trilepida jani* (*n*=1): **BRAZIL:** MINAS GERAIS: *Ouro Preto*: LZV 813S.

*Trilepida joshuai* (*n*=1): **COLOMBIA:** ANTIOQUIA: *Jericó*: IBSP 8919.

*Trilepida koppesi* (*n*= 1): **BRAZIL**: GOIÁS: Aporé: MNRJ 24715.

*Trilepida macrolepis* (*n*=1): **BRAZIL**: PARÁ: *Parauapebas*: Floresta Nacional de Carajás: MPEG 23017.

*Trilepida salgueiroi* (*n*=1). **BRAZIL:** ESPÍTITO SANTO: *Governador Lindemberg*: MNRJ 12132.
