# Supplementary material for: Moving beyond the surface: Comparative head and neck myology of threadsnakes (Epictinae, Leptotyphlopidae, Serpentes), with comments on the ‘scolecophidian’ muscular system
Source: PLoS One. 2019 Jul 18;14(7):e0219661. doi: 10.1371/journal.pone.0219661 (PMC6638936; doi:10.1371/journal.pone.0219661)
Supplement: S1 Table — Abbreviations are as follows: ? = Unknown, NV = Not variable, N/A = Not applicable, BV = Bilaterally variable. “V” = vertebrae of origin where V1 = atlas, V2 –axis, V3 –first thoracolombar vertebra and so on. (DOCX) [file pone.0219661.s002.docx]

S1 Table. Synthesis of inter- and intraspecific variability of the head and neck muscles for members of the Subfamily Epictinae. Abbreviations are as follows: ? = Unknown, NV = Not variable, N/A = Not applicable, BV = Bilaterally variable. “V” = vertebrae of origin where V1 = atlas, V2 – axis, V3 – first thoracolombar vertebra and so on.

| **Species** | **Condition** | **Origin** | **Insertion** | **Anteriormost point of origin** | **Posteriormost point of origin** | **Anteriormost point of insertion** | **Posteriormost point of insertion** | **Notes** |  |  |  |  |  |  |  |  |  |
| --- | --- | --- | --- | --- | --- | --- | --- | --- | --- | --- | --- | --- | --- | --- | --- | --- | --- |
| ***Musculus levator anguli oris*** | | | | | | | | |  |  |  |  |  |  |  |  |  |
| *Epictia ater* | Present | Narrow | NV | First half of frontal lateral face | Frontoparietal suture | NV | NV | - |  |  |  |  |  |  |  |  |  |
| *Epictia phenops* | Present | Narrow | NV | Anterior limit parietal | First third of parietal | NV | NV | - |  |  |  |  |  |  |  |  |  |
| *Epictia tenella* | Present | Narrow | NV | Anterior limit parietal | First fourth or third of parietal | NV | NV | - |  |  |  |  |  |  |  |  |  |
| *Mitophis lepitepileptus* | Present | Very narrow | NV | First quarter of frontal lateral face | Frontal posterior limit | NV | NV | - |  |  |  |  |  |  |  |  |  |
| *Rena dulcis* | Present | Wide | NV | Posterior limit frontal | First half of parietal | NV | NV | BV |  |  |  |  |  |  |  |  |  |
| *Rena humilis* | Present | Wide | NV | Posterior limit frontal (n=1;50%) or anterior limit frontal (n=1; 50%) | First half of parietal (n=1; 50%) or frontoparietal suture (n=1; 50%) | NV | NV | - |  |  |  |  |  |  |  |  |  |
| *Rena segrega* | Present | Wide | NV | Posterior limit frontal | First half of parietal | NV | NV | - |  |  |  |  |  |  |  |  |  |
| *Rena unguirostris* | Present | Narrow | NV | First half of frontal lateral face | Frontoparietal suture | NV | NV | Fibers not converging ventrally |  |  |  |  |  |  |  |  |  |
| *Siagonodon cupinensis* | Present | Wide | NV | First half of frontal lateral face | First half of parietal | NV | NV | - |  |  |  |  |  |  |  |  |  |
| *Tetracheilostoma bilineatum* | Present | Narrow | NV | Short posterior area of frontal | Short anterior area of parietal | NV | NV | - |  |  |  |  |  |  |  |  |  |
| *Trilepida brasiliensis* | Present | Wide | NV | First half of frontal lateral face | First half of parietal | NV | NV | - |  |  |  |  |  |  |  |  |  |
| *Trilepida dimidiata* | Present | Wide | NV | First half of frontal lateral face | First half of parietal | NV | NV | - |  |  |  |  |  |  |  |  |  |
| *Trilepida fuliginosa* | Present | Wide | NV | First half of frontal lateral face | First half of parietal | NV | NV | - |  |  |  |  |  |  |  |  |  |
| *Trilepida jani* | Present | Wide | NV | First half of frontal lateral face | First half of parietal | NV | NV | - |  |  |  |  |  |  |  |  |  |
| *Trilepida joshuai* | Present | Wide | NV | First half of frontal lateral face | First half of parietal | NV | NV | - |  |  |  |  |  |  |  |  |  |
| *Trilepida koppesi* | Present | Wide | NV | First half of frontal lateral face | First half of parietal | NV | NV | - |  |  |  |  |  |  |  |  |  |
| *Trilepida macrolepis* | Present | Wide | NV | First half of frontal lateral face | First half of parietal | NV | NV | - |  |  |  |  |  |  |  |  |  |
| *Trilepida salgueiroi* | Present | Wide | NV | First half of frontal lateral face | First half of parietal | NV | NV | - |  |  |  |  |  |  |  |  |  |
| ***Musculus adductor mandibulae externus profundus*** | | | | | | | | |  |  |  |  |  |  |  |  |  |
| *Epictia ater* | Present | Quadrate proximal head *via* tendon, lateral and ventral lamina | Posterior lamina of the compound bone supracotylar process of compound bone *via* tendon | NV | NV | NV | NV | Origin: a few bundle of fibers attach to the lateral lamina of the prootic adjacent to the quadrate |  |  |  |  |  |  |  |  |  |
| *Epictia phenops* | Present | Quadrate proximal head *via* tendon | Through tendon onto the posterior lamina of the supracotylar process of the compound bone | NV | NV | NV | NV | - |  |  |  |  |  |  |  |  |  |
| *Epictia tenella* | Present | Quadrate proximal head *via* tendon; lateral, dorsal and ventral lamina | Through tendon onto the posterior (n=1, 50%) or medial (n=1; 50%) lamina of the supracotylar process of the compound bone | NV | NV | NV | NV | - |  |  |  |  |  |  |  |  |  |
| *Mitophis lepitepileptus* | Present | Quadrate lateral lamina; quadrate proximal head cartilage | Dorsal-posterior region of the supracotylar process lateral lamina | NV | NV | NV | NV | - |  |  |  |  |  |  |  |  |  |
| *Rena dulcis* | Present | Both the cartilage of the quadrate proximal head and also the dorsal lamina of the posterior region of the quadrate | posterior-dorsal lamina of supracotilar process, with some fibers inserting more medially | NV | NV | NV | NV | - |  |  |  |  |  |  |  |  |  |
| *Rena humilis* | Present | Both the cartilage of the quadrate proximal head and also the dorsal lamina of the posterior region of the quadrate | Tendon attachs to the posterior lamina of the supracotylar process. However, fibers dorsal to the tendon attach to the dorsal-medial region of the supracotylar process, while fibers ventral to the tendon attach laterally in the supracotylar process | NV | NV | NV | NV | - |  |  |  |  |  |  |  |  |  |
| *Rena segrega* | Present | Both the cartilage of the quadrate proximal head and also the dorsal lamina of the posterior region of the quadrate | Fibers insert exclusively through the tendon, which itself inserts on the posterior-medial portion of the supracotilar process | NV | NV | NV | NV | - |  |  |  |  |  |  |  |  |  |
| *Rena unguirostris* | Present | Narrow, with long tendon to the posterior portion of the quadrate; fibers originate from the proximal epiphysis and also along the quadrate lateral lamina | Insertion occurs (slightly more lateral) on the dorsal-posterior lamina of the supracotilar process | NV | NV | NV | NV | - |  |  |  |  |  |  |  |  |  |
| *Siagonodon cupinensis* | Present | Its origin includes the cartilage of the quadrate proximal epiphysis and all the quadrate lateral lamina, with fibers oriented anteriorly to insert onto the medial portion of the massive unit of fibers | Fibers insert through a tendon in the posterior-dorsal lamina of the supracotilar process Some dorsalmost and ventralmost fibers also insert medially and laterally to the supracotilar process of the quadrate. | NV | NV | NV | NV | - |  |  |  |  |  |  |  |  |  |
| *Tetracheilostoma bilineatum* | Present | Its origin also includes all the lateral, dorsal and ventral lamina of the quadrate, with fibers oriented anteriorly inserting on the medial portion of the extensive mass of fibers | Fibers insert through a tendon in the Laterodorsal lamina of quadrate supracotylar process. | NV | NV | NV | NV | - |  |  |  |  |  |  |  |  |  |
| *Trilepida brasiliensis* | Present | Its origin also includes the lateral lamina of the quadrate in its posterior half | Posterior lamina of the supracotylar process | NV | NV | NV | NV | - |  |  |  |  |  |  |  |  |  |
| *Trilepida dimidiata* | Present | Fibers do not originate in the lateral lamina, but only in the ventral portion of the quadrate in its posterior half | Posterior lamina of the supracotylar process | NV | NV | NV | NV | - |  |  |  |  |  |  |  |  |  |
| *Trilepida fuliginosa* | Present | Its origin also includes the lateral lamina of the quadrate in its posterior half | Posterior lamina of the supracotylar process | NV | NV | NV | NV | - |  |  |  |  |  |  |  |  |  |
| *Trilepida jani* | Present | Its origin also Includes the lateral lamina of the quadrate in its posterior half | Posterior lamina of the supracotylar process | NV | NV | NV | NV | - |  |  |  |  |  |  |  |  |  |
| *Trilepida joshuai* | Present | Fibers originate in the dorsolateral lamina of the quadrate along its whole extension | Posterior lamina of supracotylar process | NV | NV | NV | NV | - |  |  |  |  |  |  |  |  |  |
| *Trilepida koppesi* | Present | Fibers originate in the dorsolateral lamina of the quadrate along its whole extension | Posterior lamina of the supracotylar process | NV | NV | NV | NV | - |  |  |  |  |  |  |  |  |  |
| *Trilepida macrolepis* | Present | No fibers originate on the lateral, dorsal or ventral portion of the quadrate | Posterior lamina of supracotylar process | NV | NV | NV | NV | - |  |  |  |  |  |  |  |  |  |
| *Trilepida salgueiroi* | Present | Fibers originate in the dorsolateral lamina of the quadrate along its whole extension | Posterior lamina of the supracotylar process | NV | NV | NV | NV | - |  |  |  |  |  |  |  |  |  |
| ***Musculus adductor mandibulae posterior*** | | | | | | | | |  |  |  |  |  |  |  |  |  |
| *Epictia ater* | Present | ? | ? | ? | ? | ? | ? | - |  |  |  |  |  |  |  |  |  |
| *Epictia phenops* | Present | Narrow | Medial face of compound bone ventral to the supracotylar process | Medial face of quadrate proximal epyphisis | Medial face of quadrate proximal epyphisis | N/A | N/A | - |  |  |  |  |  |  |  |  |  |
| *Epictia tenella* | Present (n=1); Absent (n=1) | Narrow | Medial face of compound bone ventral to the supracotylar process | Medial face of quadrate proximal epyphisis | Medial face of quadrate proximal epyphisis | N/A | N/A | - |  |  |  |  |  |  |  |  |  |
| *Mitophis lepitepileptus* | Present | Narrow | Medial face of supracotylar process | Medial face anterior to quadrate proximal epyphisis | Medial face anterior to quadrate proximal epyphisis | N/A | N/A | - |  |  |  |  |  |  |  |  |  |
| *Rena dulcis* | Present | Wide | Medial face of compound bone at the articular lamina | Medial face anterior to quadrate distal epyphisis | Medial face anterior to quadrate proximal epyphisis | N/A | N/A | - |  |  |  |  |  |  |  |  |  |
| *Rena humilis* | Present | Wide | Medial face of compound bone at the articular lamina | Medial face anterior to quadrate distal epyphisis | Medial face anterior to quadrate proximal epyphisis | N/A | N/A | - |  |  |  |  |  |  |  |  |  |
| *Rena segrega* | Absent | N/A | N/A | N/A | N/A | N/A | N/A | - |  |  |  |  |  |  |  |  |  |
| *Rena unguirostris* | Absent | N/A | N/A | N/A | N/A | N/A | N/A | - |  |  |  |  |  |  |  |  |  |
| *Siagonodon cupinensis* | Present | ? | ? | ? | ? | ? | ? | - |  |  |  |  |  |  |  |  |  |
| *Tetracheilostoma bilineatum* | Present | Wide | Medial face of compound bone articular lamina | Medial face anterior to quadrate distal epyphisis | Medial face anterior to quadrate proximal epyphisis | N/A | N/A | - |  |  |  |  |  |  |  |  |  |
| *Trilepida brasiliensis* | Absent | N/A | N/A | N/A | N/A | N/A | N/A | - |  |  |  |  |  |  |  |  |  |
| *Trilepida dimidiata* | Absent | N/A | N/A | N/A | N/A | N/A | N/A | - |  |  |  |  |  |  |  |  |  |
| *Trilepida fuliginosa* | Present | Wide | Medial face of compound bone articular lamina | Medial face anterior to quadrate distal epyphisis | Medial face anterior to quadrate proximal epyphisis | N/A | N/A | - |  |  |  |  |  |  |  |  |  |
| *Trilepida jani* | Absent | N/A | N/A | N/A | N/A | N/A | N/A | - |  |  |  |  |  |  |  |  |  |
| *Trilepida joshuai* | Present | Wide | Medial face of compound bone articular lamina | Medial face anterior to quadrate distal epyphisis | Medial face anterior to quadrate proximal epyphisis | N/A | N/A | - |  |  |  |  |  |  |  |  |  |
| *Trilepida koppesi* | Present | Wide | Medial face of compound bone articular lamina | Medial face anterior to quadrate distal epyphisis | Medial face anterior to quadrate proximal epyphisis | N/A | N/A | - |  |  |  |  |  |  |  |  |  |
| *Trilepida macrolepis* | Absent | N/A | N/A | N/A | N/A | N/A | N/A | - |  |  |  |  |  |  |  |  |  |
| *Trilepida salgueiroi* | Present | Wide | Medial face of compound bone articular lamina | Medial face anterior to quadrate distal epyphisis | Medial face anterior to quadrate proximal epyphisis | N/A | N/A | - |  |  |  |  |  |  |  |  |  |
| ***Musculus pseudotemporalis*** | | | | | | | | |  | | |  |  |  |  | N/A | N/A |
| *Epictia ater* | Present | Anterolateral face of parietal and frontal | Medial area of prearticular lamina | N/A | N/A | N/A | N/A | - |  |  |  |  |  |  |  |  |  |
| *Epictia phenops* | Present | Anterolateral face of parietal | Medial area of prearticular lamina | N/A | N/A | N/A | N/A | - |  |  |  |  |  |  |  |  |  |
| *Epictia tenella* | Present | Anterolateral face of parietal and frontal | Medial area of prearticular lamina | N/A | N/A | N/A | N/A | - |  |  |  |  |  |  |  |  |  |
| *Mitophis lepitepileptus* | Present | Lateral face of parietal and frontal | ? | N/A | N/A | N/A | N/A | - |  |  |  |  |  |  |  |  |  |
| *Rena dulcis* | Present | Anterolateral face of parietal | Posterior area of prearticular lamina | N/A | N/A | N/A | N/A | - |  |  |  |  |  |  |  |  |  |
| *Rena humilis* | Present | Anterolateral face of parietal | Posterior area of prearticular lamina | N/A | N/A | N/A | N/A | - |  |  |  |  |  |  |  |  |  |
| *Rena segrega* | Present | Anterolateral face of parietal, including frontal (n=1) or not (n=1) | Posterior area of prearticular lamina | N/A | N/A | N/A | N/A | - |  |  |  |  |  |  |  |  |  |
| *Rena unguirostris* | Present | Anterolateral face of parietal | Posterior area of prearticular lamina | N/A | N/A | N/A | N/A | - |  |  |  |  |  |  |  |  |  |
| *Siagonodon cupinensis* | Present | Anterolateral face of parietal | Posterior area of prearticular lamina | N/A | N/A | N/A | N/A | - |  |  |  |  |  |  |  |  |  |
| *Tetracheilostoma bilineatum* | Present | Anterolateral face of parietal | Coronoid process of prearticular lamina | N/A | N/A | N/A | N/A | - |  |  |  |  |  |  |  |  |  |
| *Trilepida brasiliensis* | Present | Posterolateral face of parietal | ? | N/A | N/A | N/A | N/A | - |  |  |  |  |  |  |  |  |  |
| *Trilepida dimidiata* | Present | Anterolateral face of parietal | Posterior area of prearticular lamina | N/A | N/A | N/A | N/A | - |  |  |  |  |  |  |  |  |  |
| *Trilepida fuliginosa* | Present | Posterolateral face of parietal | Medial area of prearticular lamina | N/A | N/A | N/A | N/A | - |  |  |  |  |  |  |  |  |  |
| *Trilepida jani* | Present | Posterolateral face of parietal | Posterior area of prearticular lamina | N/A | N/A | N/A | N/A | - |  |  |  |  |  |  |  |  |  |
| *Trilepida joshuai* | Present | Posterolateral face of parietal | Medial area of prearticular lamina | N/A | N/A | N/A | N/A | - |  |  |  |  |  |  |  |  |  |
| *Trilepida koppesi* | Present | Posterolateral face of parietal | Posterior area of prearticular lamina | N/A | N/A | N/A | N/A | - |  |  |  |  |  |  |  |  |  |
| *Trilepida macrolepis* | Present | Posterolateral face of parietal | ? | N/A | N/A | N/A | N/A | - |  |  |  |  |  |  |  |  |  |
| *Trilepida salgueiroi* | Present | Posterolateral face of parietal | Posterior area of prearticular lamina | N/A | N/A | N/A | N/A | - |  |  |  |  |  |  |  |  |  |
| ***Musculus protractor pterygoidei*** | | | | | | | | |  |  |  |  |  |  |  |  |  |
| *Epictia ater* | Present | Parietal and parabasisphenoid | NV | N/A | N/A | At the level of frontoparietal suture | Posterior limit of pterygoid | - |  |  |  |  |  |  |  |  |  |
| *Epictia phenops* | Present | Parietal and parabasisphenoid | NV | N/A |  | At the level of frontoparietal suture | Posterior limit of pterygoid | - |  |  |  |  |  |  |  |  |  |
| *Epictia tenella* | Present | Parietal and parabasisphenoid | NV | N/A | N/A | Half of pterygoid (n=1) or at the level of frontoparietal suture (n=1) | Posterior limit of pterygoid | - |  |  |  |  |  |  |  |  |  |
| *Mitophis lepitepileptus* | Present | Parietal | NV | N/A | N/A | At the level of frontoparietal suture | Posterior limit of pterygoid | - |  |  |  |  |  |  |  |  |  |
| *Rena dulcis* | Present | Parietal and parabasisphenoid | NV | N/A | N/A | Anterior area of pterygoid | Posterior limit of pterygoid | - |  |  |  |  |  |  |  |  |  |
| *Rena humilis* | Present | Parietal and parabasisphenoid | NV | N/A | N/A | Anterior area of pterygoid | Posterior limit of pterygoid | - |  |  |  |  |  |  |  |  |  |
| *Rena segrega* | Present | Parietal and parabasisphenoid | NV | N/A | N/A | Half of pterygoid | Posterior limit of pterygoid | - |  |  |  |  |  |  |  |  |  |
| *Rena unguirostris* | Present | Parietal and parabasisphenoid | NV | N/A | N/A | Anterior area of pterygoid | Posterior limit of pterygoid | - |  |  |  |  |  |  |  |  |  |
| *Siagonodon cupinensis* | Present | Parietal and parabasisphenoid | NV | N/A | N/A | At the level of frontoparietal suture | Posterior limit of pterygoid | - |  |  |  |  |  |  |  |  |  |
| *Tetracheilostoma bilineatum* | Present | Parietal | NV | N/A | N/A | At the level of frontoparietal suture | Posterior limit of pterygoid | - |  |  |  |  |  |  |  |  |  |
| *Trilepida brasiliensis* | Present | Parietal and parabasisphenoid | NV | N/A | N/A | At the level of frontoparietal suture | Posterior limit of pterygoid | - |  |  |  |  |  |  |  |  |  |
| *Trilepida dimidiata* | Present | Parietal and parabasisphenoid | NV | N/A | N/A | At the level of frontoparietal suture | Posterior limit of pterygoid | - |  |  |  |  |  |  |  |  |  |
| *Trilepida fuliginosa* | Present | Parietal and parabasisphenoid | NV | N/A | N/A | At the level of frontoparietal suture | Posterior limit of pterygoid | - |  |  |  |  |  |  |  |  |  |
| *Trilepida jani* | Present | Parietal and parabasisphenoid | NV | N/A | N/A | At the level of frontoparietal suture | Posterior limit of pterygoid | - |  |  |  |  |  |  |  |  |  |
| *Trilepida joshuai* | Present | Parietal | NV | N/A | N/A | Half of pterygoid | Posterior limit of pterygoid | - |  |  |  |  |  |  |  |  |  |
| *Trilepida koppesi* | Present | Parietal and parabasisphenoid | NV | N/A | N/A | At the level of frontoparietal suture | Posterior limit of pterygoid | - |  |  |  |  |  |  |  |  |  |
| *Trilepida macrolepis* | Present | Parietal and parabasisphenoid | NV | N/A | N/A | At the level of frontoparietal suture | Posterior limit of pterygoid | - |  |  |  |  |  |  |  |  |  |
| *Trilepida salgueiroi* | Present | Parietal and parabasisphenoid | NV | N/A | N/A | At the level of frontoparietal suture | Posterior limit of pterygoid | - |  |  |  |  |  |  |  |  |  |
| ***Musculus pterygoideus acessorius* *anterior*** | | | | | | | | |  |  |  |  |  |  |  |  |  |
| *Epictia ater* | Present | Palatine ventral face and frontal | Posterior medial face of retroarticular process | N/A | N/A | N/A | N/A | - |  |  |  |  |  |  |  |  |  |
| *Epictia phenops* | Present | Palatine ventral face | Posterior medial face of retroarticular process | N/A | N/A | N/A | N/A | - |  |  |  |  |  |  |  |  |  |
| *Epictia tenella* | Present | Palatine ventral face | Anterior medial face of quadrate | N/A | N/A | N/A | N/A | - |  |  |  |  |  |  |  |  |  |
| *Mitophis lepitepileptus* | Present | Palatine lateral face | Articular lamina of compound bone | N/A | N/A | N/A | N/A | - |  |  |  |  |  |  |  |  |  |
| *Rena dulcis* | Present | Ventral and lateral faces of palatine | ? | N/A | N/A | N/A | N/A | - |  |  |  |  |  |  |  |  |  |
| *Rena humilis* | Present | Ventral and lateral faces of palatine and pterygoid | *Via* tendon onto the anteroventral face of quadrate | N/A | N/A | N/A | N/A | - |  |  |  |  |  |  |  |  |  |
| *Rena segrega* | Present | Ventral and lateral faces of palatine | Ventromedial face of retroarticular process | N/A | N/A | N/A | N/A | - |  |  |  |  |  |  |  |  |  |
| *Rena unguirostris* | Present | Ventral and lateral faces of palatine and pterygoid | *Via* tendon onto the anteroventral face of quadrate | N/A | N/A | N/A | N/A | - |  |  |  |  |  |  |  |  |  |
| *Siagonodon cupinensis* | Present | Palatine ventral face and pterygoid | Anteromedial face of quadrate | N/A | N/A | N/A | N/A | Continuous to the *M. pterygoideus acessorius posterior* |  |  |  |  |  |  |  |  |  |
| *Tetracheilostoma bilineatum* | Present | Ventral and lateral faces of palatine | Anteromedial face of quadrate | N/A | N/A | N/A | N/A | Insertion area posterior to the *M. pterygoideus* insertion |  |  |  |  |  |  |  |  |  |
| *Trilepida brasiliensis* | Present | Palatine ventral face and pterygoid | Anteromedial face of quadrate | N/A | N/A | N/A | N/A | - |  |  |  |  |  |  |  |  |  |
| *Trilepida dimidiata* | Present | Palatine ventral face and pterygoid | Anteromedial face of quadrate | N/A | N/A | N/A | N/A | - |  |  |  |  |  |  |  |  |  |
| *Trilepida fuliginosa* | Present | Palatine ventral face and pterygoid | Anteromedial face of quadrate | N/A | N/A | N/A | N/A | - |  |  |  |  |  |  |  |  |  |
| *Trilepida jani* | Present | Palatine ventral face and pterygoid | Anteromedial face of quadrate | N/A | N/A | N/A | N/A | - |  |  |  |  |  |  |  |  |  |
| *Trilepida joshuai* | Present | Palatine ventral face and pterygoid | Anteromedial face of quadrate | N/A | N/A | N/A | N/A | - |  |  |  |  |  |  |  |  |  |
| *Trilepida koppesi* | Present | Palatine ventral face and pterygoid | Anteromedial face of quadrate | N/A | N/A | N/A | N/A | - |  |  |  |  |  |  |  |  |  |
| *Trilepida macrolepis* | Present | Palatine ventral face and pterygoid | Anteromedial face of quadrate | N/A | N/A | N/A | N/A | - |  |  |  |  |  |  |  |  |  |
| *Trilepida salgueiroi* | Present | Palatine ventral face and pterygoid | Anteromedial face of quadrate | N/A | N/A | N/A | N/A | - |  |  |  |  |  |  |  |  |  |
| ***Musculus pterygoideus acessorius posterior*** | | | | | | | | |  |  |  |  |  |  |  |  |  |
| *Epictia ater* | Absent | N/A | N/A | N/A | N/A | N/A | N/A | - |  |  |  |  |  |  |  |  |  |
| *Epictia phenops* | Absent | N/A | N/A | N/A | N/A | N/A | N/A | - |  |  |  |  |  |  |  |  |  |
| *Epictia tenella* | Present (n=1) or absent (n=1) | Lateroventral face of parietal and pterygoid | NV | N/A | N/A | N/A | N/A | - |  |  |  |  |  |  |  |  |  |
| *Mitophis lepitepileptus* | Absent | N/A | N/A | N/A | N/A | N/A | N/A | - |  |  |  |  |  |  |  |  |  |
| *Rena dulcis* | Present | Lateroventral face of parietal and pterygoid | NV | N/A | N/A | N/A | N/A | - |  |  |  |  |  |  |  |  |  |
| *Rena humilis* | Present | Lateroventral face of parietal and pterygoid | NV | N/A | N/A | N/A | N/A | - |  |  |  |  |  |  |  |  |  |
| *Rena segrega* | Present | Parietal-parabasisphenoid suture | NV | N/A | N/A | N/A | N/A | - |  |  |  |  |  |  |  |  |  |
| *Rena unguirostris* | Absent | N/A | N/A | N/A | N/A | N/A | N/A | - |  |  |  |  |  |  |  |  |  |
| *Siagonodon cupinensis* | Present | See notes | N/A | N/A | N/A | N/A | N/A | Indistinct from *M. pterygoideus acessorius anterior* (see *M. pterygoideus acessorius anterior*) |  |  |  |  |  |  |  |  |  |
| *Tetracheilostoma bilineatum* | Absent | N/A | N/A | N/A | N/A | N/A | N/A | - |  |  |  |  |  |  |  |  |  |
| *Trilepida brasiliensis* | Present | Lateroventral face of parietal and pterygoid | NV | N/A | N/A | N/A | N/A | - |  |  |  |  |  |  |  |  |  |
| *Trilepida dimidiata* | Absent | N/A | N/A | N/A | N/A | N/A | N/A | - |  |  |  |  |  |  |  |  |  |
| *Trilepida fuliginosa* | Absent | N/A | N/A | N/A | N/A | N/A | N/A | - |  |  |  |  |  |  |  |  |  |
| *Trilepida jani* | Absent | N/A | N/A | N/A | N/A | N/A | N/A | - |  |  |  |  |  |  |  |  |  |
| *Trilepida joshuai* | Absent | N/A | N/A | N/A | N/A | N/A | N/A | - |  |  |  |  |  |  |  |  |  |
| *Trilepida koppesi* | Absent | N/A | N/A | N/A | N/A | N/A | N/A | - |  |  |  |  |  |  |  |  |  |
| *Trilepida macrolepis* | Absent | N/A | N/A | N/A | N/A | N/A | N/A | - |  |  |  |  |  |  |  |  |  |
| *Trilepida salgueiroi* | Present | Lateroventral face of parietal and pterygoid | NV | N/A | N/A | N/A | N/A | - |  |  |  |  |  |  |  |  |  |
| ***Musculus intermandibularis anterior*** | | | | | | | | |  |  |  |  |  |  |  |  |  |
| *Epictia ater* | Present | Posterior from dentary | Posterior | At the level of coronoid posterior limit | N/A | N/A | N/A | - |  |  |  |  |  |  |  |  |  |
| *Epictia phenops* | Present | Anterior from dentary | Anterior | At the level of the dorsoposterior process of dentary | N/A | N/A | N/A | - |  |  |  |  |  |  |  |  |  |
| *Epictia tenella* | Present | Anterior (n=1) or posterior (n=1) from dentary | Anterior | At the level of the dorsoposterior process of dentary | N/A | N/A | N/A | - |  |  |  |  |  |  |  |  |  |
| *Mitophis lepitepileptus* | Present | Anterior from dentary | Anterior | At the level of the dorsoposterior process of dentary | N/A | N/A | N/A | - |  |  |  |  |  |  |  |  |  |
| *Rena dulcis* | Present | ? | ? |  | N/A | N/A | N/A | - |  |  |  |  |  |  |  |  |  |
| *Rena humilis* | Present | ? | ? |  | N/A | N/A | N/A | - |  |  |  |  |  |  |  |  |  |
| *Rena segrega* | Present | Anterior from dentary | Anterior | At the level of the dorsoposterior process of dentary | N/A | N/A | N/A | - |  |  |  |  |  |  |  |  |  |
| *Rena unguirostris* | Present | Anterior from dentary | ? | ? | N/A | N/A | N/A | - |  |  |  |  |  |  |  |  |  |
| *Siagonodon cupinensis* | Present | Anteriorly from dentary | Anterior | At the level of posterior limit of dental concha | N/A | N/A | N/A | - |  |  |  |  |  |  |  |  |  |
| *Tetracheilostoma bilineatum* | Present | Anteriorly from dentary | ? | ? | N/A | N/A | N/A | - |  |  |  |  |  |  |  |  |  |
| *Trilepida brasiliensis* | Present | Medially from dentary | ? | ? | N/A | N/A | N/A | - |  |  |  |  |  |  |  |  |  |
| *Trilepida dimidiata* | Present | Medially from dentary | ? | ? | N/A | N/A | N/A | - |  |  |  |  |  |  |  |  |  |
| *Trilepida fuliginosa* | Present | Medially from dentary | ? | ? | N/A | N/A | N/A | - |  |  |  |  |  |  |  |  |  |
| *Trilepida jani* | Present | Medially from dentary | ? | ? | N/A | N/A | N/A | - |  |  |  |  |  |  |  |  |  |
| *Trilepida joshuai* | Present | Medially from dentary | ? | ? | N/A | N/A | N/A | - |  |  |  |  |  |  |  |  |  |
| *Trilepida koppesi* | Present | Medially from dentary | ? | ? | N/A | N/A | N/A | - |  |  |  |  |  |  |  |  |  |
| *Trilepida macrolepis* | Present | Medially from dentary | ? | ? | N/A | N/A | N/A | - |  |  |  |  |  |  |  |  |  |
| *Trilepida salgueiroi* | Present | Medially from dentary | ? | ? | N/A | N/A | N/A | - |  |  |  |  |  |  |  |  |  |
| ***Musculus intermandibularis posterior*, *pars anterior*** | | | | | | | | |  |  |  |  |  |  |  |  |  |
| *Epictia ater* | Present | Broad | NV | N/A | N/A | Posterior limit or articular | Cotylar head of quadrate | - |  |  |  |  |  |  |  |  |  |
| *Epictia phenops* | Present | Moderate | NV | N/A | N/A | Posterior limit of prearticular lamina | Posterior half of articular | - |  |  |  |  |  |  |  |  |  |
| *Epictia tenella* | Present | Moderate | NV | N/A | N/A | Posterior limit of prearticular lamina | Posterior half of articular | - |  |  |  |  |  |  |  |  |  |
| *Mitophis lepitepileptus* | Present | Narrow | NV | N/A | N/A | Anterior limit of prearticular lamina | Posterior limit of articular lamina | - |  |  |  |  |  |  |  |  |  |
| *Rena dulcis* | Present | Narrow | NV | N/A | N/A | Anterior limit of prearticular lamina | Posterior limit of articular lamina | - |  |  |  |  |  |  |  |  |  |
| *Rena humilis* | Present | Moderate | NV | N/A | N/A | Posterior ½ of angular | Retroarticular process | - |  |  |  |  |  |  |  |  |  |
| *Rena segrega* | Present | Moderate | NV | N/A | N/A | Posterior ½ of angular | Retroarticular process | - |  |  |  |  |  |  |  |  |  |
| *Rena unguirostris* | Present | Wide | NV | N/A | N/A | Posterior dentary | Posterior limit of prearticular face | - |  |  |  |  |  |  |  |  |  |
| *Siagonodon cupinensis* | Present | See notes | NV | N/A | N/A | Anteriormost unit onto the lateroventral face of dentary | Posteriormost unit onto the lateral face the angular | Two units present |  |  |  |  |  |  |  |  |  |
| *Tetracheilostoma bilineatum* | Present | Narrow | NV | N/A | N/A | Half of angular | Half of prearticular lamina | - |  |  |  |  |  |  |  |  |  |
| *Trilepida brasiliensis* | Present | Wide | NV | N/A | N/A | Anterior limit of angular | Posterior limit of prearticular lamina | - |  |  |  |  |  |  |  |  |  |
| *Trilepida dimidiata* | Present | Narrow | NV | N/A | N/A | Anterior limit of angular | Anterior limit of angular | - |  |  |  |  |  |  |  |  |  |
| *Trilepida fuliginosa* | Present | Narrow | NV | N/A | N/A | Anterior limit of angular | Anterior limit of angular | - |  |  |  |  |  |  |  |  |  |
| *Trilepida jani* | Present | Narrow | NV | N/A | N/A | Posterior limit of angular | Anterior prearticular face | - |  |  |  |  |  |  |  |  |  |
| *Trilepida joshuai* | Present | Narrow | NV | N/A | N/A | Anterior limit of angular | Anterior limit of angular | - |  |  |  |  |  |  |  |  |  |
| *Trilepida koppesi* | Present | Wide | NV | N/A | N/A | Anterior limit of angular | Posterior limit of prearticular lamina | - |  |  |  |  |  |  |  |  |  |
| *Trilepida macrolepis* | Present | Moderate | NV | N/A | N/A | Posterior limit of angular | Anterior retroarticular process | - |  |  |  |  |  |  |  |  |  |
| *Trilepida salgueiroi* | Present | ? | NV | N/A | N/A | ? | ? | - |  |  |  |  |  |  |  |  |  |
| ***Musculus intermandibularis posterior, pars posterior*** | | | | | | | | |  |  |  |  |  |  |  |  |  |
| *Epictia ater* | Present | Moderate | NV | N/A | N/A | Anteroventral face of compound bone at the level of the surangular foramen | Posterior limit of retroarticular process | - |  |  |  |  |  |  |  |  |  |
| *Epictia phenops* | Present | Moderate | NV | N/A | N/A | Ventral face of compound bone at the level of the surangular foramen | Posterior limit of retroarticular process | - |  |  |  |  |  |  |  |  |  |
| *Epictia tenella* | Present | Narrow | NV | N/A | N/A | Ventral face of compound bone at the level of posterior limit of coronoid | Posterior limit of compound bone | Insertion site does not include the retroarticular process |  |  |  |  |  |  |  |  |  |
| *Mitophis lepitepileptus* | Present | Narrow | NV | N/A | N/A | Ventral face of compound bone at the level of ½ extension of coronoid | Retroarticular process | - |  |  |  |  |  |  |  |  |  |
| *Rena dulcis* | Present | Moderate | NV | N/A | N/A | Ventral face of compound bone at the level of ½ extension of posterior surangular foramen | Retroarticular process | - |  |  |  |  |  |  |  |  |  |
| *Rena humilis* | Present | Moderate | NV | N/A | N/A | Ventral face of compound bone at the level of ½ extension of posterior surangular foramen | Retroarticular process | - |  |  |  |  |  |  |  |  |  |
| *Rena segrega* | Present | Wide | NV | N/A | N/A | Ventral face of compound bone at the level anterior limit of coronoid | Retroarticular process | - |  |  |  |  |  |  |  |  |  |
| *Rena unguirostris* | Present | Narrow | NV | N/A | N/A | Anterior limit of retroarticular process | Retroarticular process | - |  |  |  |  |  |  |  |  |  |
| *Siagonodon cupinensis* | Present | Narrow | NV | N/A | N/A | Ventral face of compound bone at the level of posterior limit of coronoid | Retroarticular process | - |  |  |  |  |  |  |  |  |  |
| *Tetracheilostoma bilineatum* | Present | Moderate | NV | N/A | N/A | Lateroventral face of compound bone, at the level of anterior limit of coronoid | Retroarticular process | - |  |  |  |  |  |  |  |  |  |
| *Trilepida brasiliensis* | Present | Narrow | NV | N/A | N/A | Ventral face of compound bone at the level of posterior limit of posterior surangular foramen | Retroarticular process | - |  |  |  |  |  |  |  |  |  |
| *Trilepida dimidiata* | Present | Narrow | NV | N/A | N/A | Ventral face of compound bone at the level of posterior limit of posterior surangular foramen | Posterior limit of compound bone | Insertion site does not include the retroarticular process |  |  |  |  |  |  |  |  |  |
| *Trilepida fuliginosa* | Present | Extremely narrow | NV | N/A | N/A | Anterior limit of retroarticular process | Posterior limit of retroarticular process | - |  |  |  |  |  |  |  |  |  |
| *Trilepida jani* | Present | Moderate | NV | N/A | N/A | Ventral face of compound bone at the level of the area between the anterior and posterior surangular foramen | Posterior limit of compound bone | Insertion site does not include the retroarticular process |  |  |  |  |  |  |  |  |  |
| *Trilepida joshuai* | Present | Narrow | NV | N/A | N/A | Ventral face of compound bone at the level of the area of ½ the extension of posterior surangular foramen | Posterior limit of compound bone | Insertion site does not include the retroarticular process |  |  |  |  |  |  |  |  |  |
| *Trilepida koppesi* | Present | Moderate | NV | N/A | N/A | Ventral face of compound bone at the level of the area of ½ the extension of posterior surangular foramen | Retroarticular process | - |  |  |  |  |  |  |  |  |  |
| *Trilepida macrolepis* | Present | Wide | NV | N/A | N/A | Posterior ½ of angular | Posterior limit of compound bone | Insertion site does not include the retroarticular process |  |  |  |  |  |  |  |  |  |
| *Trilepida salgueiroi* | Present | ? | NV | N/A | N/A | ? | ? | - |  |  |  |  |  |  |  |  |  |
| ***Musculus cervicomandibularis*** | | | | | | | | |  |  |  |  |  |  |  |  |  |
| *Epictia ater* | Present | Insertion *via* tendon: Fibers originate in the posterior tip of the tendon | Single | At the level of V5 | NV | NV | NV | - |  |  |  |  |  |  |  |  |  |
| *Epictia phenops* | Present | Insertion *via* tendon: Fibers originate unipenately on tendon | Double | Both at the level of V4-V5 | NV | NV | NV | - |  |  |  |  |  |  |  |  |  |
| *Epictia tenella* | Present | Insertion *via* tendon: Bipenate | Double | Ventral unit at level of V3; dorsal unit at the level of V7 | Ventral unit at level of V3; dorsal unit at the level of V7 | NV | NV | - |  |  |  |  |  |  |  |  |  |
| *Mitophis lepitepileptus* | Present | Insertion *via* tendon: Bipenate | ? | ? | ? | NV | NV | - |  |  |  |  |  |  |  |  |  |
| *Rena dulcis* | Present | *Via* double tendon | Double | Dorsal at the level of V2; Ventral at V7 | Dorsal at the level of V2; Ventral at V7 | NV | NV | Insertion: The dorsal associates to dorsal fibers and attaches to laterodorsal lamina of the retroarticular process. Most of the fibers attach unipenately to tendon that inserts on the posteroventral region of the dentary |  |  |  |  |  |  |  |  |  |
| *Rena humilis* | Present | *Via* single tendon bifurcated anteriorly | ? | ? | ? | NV | NV | - |  |  |  |  |  |  |  |  |  |
| *Rena segrega* | Present | *Via* single tendon; fibers bipenate | Single | At the level of V4 | At the level of V4 | NV | NV | - |  |  |  |  |  |  |  |  |  |
| *Rena unguirostris* | Present | *Via* single tendon bifurcated anteriorly | ? | ? | ? | NV | NV | - |  |  |  |  |  |  |  |  |  |
| *Siagonodon cupinensis* | Present | *Via* single tendon; unipenate | ? | Posterior to V4 | ? | NV | NV | - |  |  |  |  |  |  |  |  |  |
| *Tetracheilostoma bilineatum* | Present | *Via* single tendon; bipenate | Single | At the level of V3 | At the level of V3 | NV | NV | - |  |  |  |  |  |  |  |  |  |
| *Trilepida brasiliensis* | Present | *Via* single tendon; bipenate | Single | ? | ? | NV | NV | - |  |  |  |  |  |  |  |  |  |
| *Trilepida dimidiata* | Present | ? | ? | ? | ? | NV | NV | - |  |  |  |  |  |  |  |  |  |
| *Trilepida fuliginosa* | Present | *Via* single tendon; bipenate | ? | At the level of V3 | At the level of V3 | NV | NV | - |  |  |  |  |  |  |  |  |  |
| *Trilepida jani* | Present | *Via* single tendon; bipenate | ? | At the level of V2 | At the level of V2 | NV | NV | - |  |  |  |  |  |  |  |  |  |
| *Trilepida joshuai* | Present | *Via* biphid tendon; bipenate | Double | Ventrally at the level of V3 and dorsally at the level of V4 | Ventrally at the level of V3 and dorsally at the level of V4 | NV | NV | - |  |  |  |  |  |  |  |  |  |
| *Trilepida koppesi* | Present | ? | ? | ? | ? | NV | NV | - |  |  |  |  |  |  |  |  |  |
| *Trilepida macrolepis* | Present | ? | ? | ? | ? | NV | NV | - |  |  |  |  |  |  |  |  |  |
| *Trilepida salgueiroi* | Present | ? | ? | ? | ? | NV | NV | - |  |  |  |  |  |  |  |  |  |
| ***Musculus depressor mandibulae*** | | | | | | | | |  |  |  |  |  |  |  |  |  |
| *Epictia ater* | Present | Posterior | NV | Posterior face of quadrate proximal epiphysis | N/A | N/A | N/A | - |  |  |  |  |  |  |  |  |  |
| *Epictia phenops* | Present | Posterior | NV | Dorsal face of quadrate proximal epiphysis | N/A | N/A | N/A | - |  |  |  |  |  |  |  |  |  |
| *Epictia tenella* | Present | Posterior | NV | Ventral face of the quadrate proximal epiphysis process | N/A | N/A | N/A | - |  |  |  |  |  |  |  |  |  |
| *Mitophis lepitepileptus* | Present | Anterior | NV | Posteroventral limit of quadrate medial face | N/A | N/A | N/A | - |  |  |  |  |  |  |  |  |  |
| *Rena dulcis* | Present | Posterior | NV | Dorsal face of quadrate proximal epiphysis | N/A | N/A | N/A | Tendon long and contouring the proximal epiphysis posteriorly |  |  |  |  |  |  |  |  |  |
| *Rena humilis* | Present | Posterior | NV | Dorsal face of quadrate proximal epiphysis | N/A | N/A | N/A | Tendon long and contouring the proximal epiphysis posteriorly |  |  |  |  |  |  |  |  |  |
| *Rena segrega* | Present | Posterior | NV | Dorsal face of quadrate proximal epiphysis | N/A | N/A | N/A | Tendon long and contouring the proximal epiphysis posteriorly |  |  |  |  |  |  |  |  |  |
| *Rena unguirostris* | Present | Posterior | NV | Dorsal face of quadrate proximal epiphysis | N/A | N/A | N/A | Tendon long and contouring the proximal epiphysis posteriorly |  |  |  |  |  |  |  |  |  |
| *Siagonodon cupinensis* | Present | Posterior | NV | Dorsal face of quadrate proximal epiphysis | N/A | N/A | N/A | Tendon long and contouring the proximal epiphysis posteriorly |  |  |  |  |  |  |  |  |  |
| *Tetracheilostoma bilineatum* | Present | Posterior | NV | Dorsal face of quadrate proximal epiphysis | N/A | N/A | N/A | Tendon long and contouring the proximal epiphysis posteriorly |  |  |  |  |  |  |  |  |  |
| *Trilepida brasiliensis* | Present | Posterior | NV | Dorsal face of quadrate proximal epiphysis | N/A | N/A | N/A | Tendon long and contouring the proximal epiphysis posteriorly |  |  |  |  |  |  |  |  |  |
| *Trilepida dimidiata* | Present | Posterior | NV | Dorsal face of quadrate proximal epiphysis | N/A | N/A | N/A | Tendon long and contouring the proximal epiphysis posteriorly |  |  |  |  |  |  |  |  |  |
| *Trilepida fuliginosa* | Present | Posterior | NV | Dorsal face of quadrate proximal epiphysis | N/A | N/A | N/A | Tendon long and contouring the proximal epiphysis posteriorly |  |  |  |  |  |  |  |  |  |
| *Trilepida jani* | Present | Posterior | NV | Dorsal face of quadrate proximal epiphysis | N/A | N/A | N/A | Tendon long and contouring the proximal epiphysis posteriorly |  |  |  |  |  |  |  |  |  |
| *Trilepida joshuai* | Present | Posterior | NV | Dorsal face of quadrate proximal epiphysis | N/A | N/A | N/A | Tendon long and contouring the proximal epiphysis posteriorly |  |  |  |  |  |  |  |  |  |
| *Trilepida koppesi* | Present | Posterior | NV | Dorsal face of quadrate proximal epiphysis | N/A | N/A | N/A | Tendon long and contouring the proximal epiphysis posteriorly |  |  |  |  |  |  |  |  |  |
| *Trilepida macrolepis* | Present | Posterior | NV | Dorsal face of quadrate proximal epiphysis | N/A | N/A | N/A | Tendon long and contouring the proximal epiphysis posteriorly |  |  |  |  |  |  |  |  |  |
| *Trilepida salgueiroi* | Present | Posterior | NV | Dorsal face of quadrate proximal epiphysis | N/A | N/A | N/A | Tendon long and contouring the proximal epiphysis posteriorly |  |  |  |  |  |  |  |  |  |
| ***Musculus cervicoquadratus*** | | | | | | | | |  |  |  |  |  |  |  |  |  |
| *Epictia ater* | Present | Anterior | Prootic and quadrate proximal epiphysis | At the level of the posterior limit of basioccipital | At the level of the posterior limit of basioccipital | N/A | N/A | - |  |  |  |  |  |  |  |  |  |
| *Epictia phenops* | Present | ? | Prootic | ? | ? | N/A | N/A | Same tendon of *M. longissimus capitis, pars transversalis capitis* |  |  |  |  |  |  |  |  |  |
| *Epictia tenella* | Present | ? | Prootic and quadrate proximal epiphysis | At the level of V4 | At the level of V4 | N/A | N/A | - |  |  |  |  |  |  |  |  |  |
| *Mitophis lepitepileptus* | Present | Anterior | Prootic | At the level of ½ extension of basioccipital | At the level of ½ extension of basioccipital | N/A | N/A | Ventral to insertion of *M. longissimus capitis, pars transversalis capitis* |  |  |  |  |  |  |  |  |  |
| *Rena dulcis* | Present | Posterior | Prootic and quadrate proximal epiphysis | At the level of V3 | At the level of V3 | N/A | N/A | In one specimen (OMNH 35564) a dorsal unit of fibers of the *M. cerviquadratus* extend posteriorly and dorsally inserting onto the rib muscles and might represent a *M. cervicoquadratus dorsalis*. |  |  |  |  |  |  |  |  |  |
| *Rena humilis* | Present | Posterior | Prootic | ? | ? | N/A | N/A | - |  |  |  |  |  |  |  |  |  |
| *Rena segrega* | Present | Posterior | Prootic | At the level of V2 | At the level of V2 | N/A | N/A | - |  |  |  |  |  |  |  |  |  |
| *Rena unguirostris* | Present | Posterior | Prootic | ? | ? | N/A | N/A | - |  |  |  |  |  |  |  |  |  |
| *Siagonodon cupinensis* | Present | Posterior | Prootic | At the level of V2 | At the level of V2 | N/A | N/A | - |  |  |  |  |  |  |  |  |  |
| *Tetracheilostoma bilineatum* | Present | Posterior | Prootic | ? | ? | N/A | N/A | - |  |  |  |  |  |  |  |  |  |
| *Trilepida brasiliensis* | Present | Posterior | Prootic | At the level of V2 | At the level of V2 | N/A | N/A | - |  |  |  |  |  |  |  |  |  |
| *Trilepida dimidiata* | Present | Posterior | Prootic | At the level of V2 | At the level of V2 | N/A | N/A | - |  |  |  |  |  |  |  |  |  |
| *Trilepida fuliginosa* | Present | Posterior | Prootic | At the level of V2 | At the level of V2 | N/A | N/A | - |  |  |  |  |  |  |  |  |  |
| *Trilepida jani* | Present | Posterior | Prootic | At the level of V2 | At the level of V2 | N/A | N/A | - |  |  |  |  |  |  |  |  |  |
| *Trilepida joshuai* | Present | Posterior | Prootic | At the level of V3 | At the level of V3 | N/A | N/A | - |  |  |  |  |  |  |  |  |  |
| *Trilepida koppesi* | Present | Posterior | Prootic | At the level of V2 | At the level of V2 | N/A | N/A | - |  |  |  |  |  |  |  |  |  |
| *Trilepida macrolepis* | Present | Posterior | Prootic | ? | ? | N/A | N/A | - |  |  |  |  |  |  |  |  |  |
| *Trilepida salgueiroi* | Present | Posterior | Prootic | ? | ? | ? | ? | - |  |  |  |  |  |  |  |  |  |
| ***Musculus obliquus capitis magnus*** | | | | | | | | |  |  |  |  |  |  |  |  |  |
| *Epictia ater* | Present | Narrow | Adjacent to prootic-supraoccipital-otooccipital suture not descending through otooccipital | Narrow dorsal area of V1 | Narrow dorsal area of V3 |  |  | - |  |  |  |  |  |  |  |  |  |
| *Epictia phenops* | Present | Wide | Adjacent to prootic-supraoccipital-otooccipital suture not descending through otooccipital | Wide dorsally at V1 | Narrow at V3 | N/A | N/A | - |  |  |  |  |  |  |  |  |  |
| *Epictia tenella* | Present | Narrow | Adjacent to prootic-supraoccipital-otooccipital suture descending (n=1) or not (n=1) through otooccipital | Narrow dorsal area of V1 | Narrow dorsal area of V3 | N/A | N/A | - |  |  |  |  |  |  |  |  |  |
| *Mitophis lepitepileptus* | Present | Wide | Prootic-otooccipital suture | Wide dorsally at V1 | Narrow at V3 | N/A | N/A | Origin area narrows posteriorly from V1 to V3 |  |  |  |  |  |  |  |  |  |
| *Rena dulcis* | Present | Wide | Adjacent to prootic-supraoccipital-otooccipital suture descending through otooccipital | Wide dorsally at V1 | Narrow at V3 | N/A | N/A | Origin area narrows posteriorly from V1 to V3 |  |  |  |  |  |  |  |  |  |
| *Rena humilis* | Present | Wide | Adjacent to prootic-supraoccipital-otooccipital suture descending through otooccipital | Wide dorsally at V1 | Narrow at V3 | N/A | N/A | Origin area narrows posteriorly from V1 to V3 |  |  |  |  |  |  |  |  |  |
| *Rena segrega* | Present | Wide | Adjacent to prootic-supraoccipital-otooccipital suture descending over otooccipital | Wide dorsally at V1 | Wide dorsal area of V3 | N/A | N/A | Area of origin narrow at V2 |  |  |  |  |  |  |  |  |  |
| *Rena unguirostris* | Present | Narrow | Prootics in dorsal and lateral face, cupraoccipital, otoccipital, and parietal | Narrow dorsal area of V1 | Narrow dorsal area of V3 | N/A | N/A | - |  |  |  |  |  |  |  |  |  |
| *Siagonodon cupinensis* | Present | Wide | Adjacent to prootic-supraoccipital-otooccipital suture descending over otooccipital | Wide dorsally at V1 | Narrow at V3 | N/A | N/A | Origin area narrows posteriorly from V1 to V3 |  |  |  |  |  |  |  |  |  |
| *Tetracheilostoma bilineatum* | Present | Narrow | Adjacent to prootic-supraoccipital-otooccipital suture descending over otooccipital | Narrow dorsal area of V1 | Narrow dorsal area of V3 | N/A | N/A | - |  |  |  |  |  |  |  |  |  |
| *Trilepida brasiliensis* | Present | Wide | Adjacent to prootic-supraoccipital-otooccipital suture descending over otooccipital | Wide dorsally at V1 | Narrow at V3 | N/A | N/A | Origin area narrows posteriorly from V1 to V3 |  |  |  |  |  |  |  |  |  |
| *Trilepida dimidiata* | Present | Wide | Adjacent to prootic-supraoccipital-otooccipital suture descending over otooccipital | Wide dorsally at V1 | Narrow at V3 | N/A | N/A | Origin area narrows posteriorly from V1 to V3 |  |  |  |  |  |  |  |  |  |
| *Trilepida fuliginosa* | Present | Wide | Adjacent to prootic-supraoccipital-otooccipital suture descending over otooccipital | Wide dorsally at V1 | Narrow at V3 | N/A | N/A | Origin area narrows posteriorly from V1 to V3 |  |  |  |  |  |  |  |  |  |
| *Trilepida jani* | Present | Wide | Adjacent to prootic-supraoccipital-otooccipital suture descending over otooccipital | Wide dorsally at V1 | Narrow at V3 | N/A | N/A | Origin area narrows posteriorly from V1 to V3 |  |  |  |  |  |  |  |  |  |
| *Trilepida joshuai* | Present | Wide | Adjacent to prootic-supraoccipital-otooccipital suture descending over otooccipital | Wide dorsally at V1 | Narrow at V3 | N/A | N/A | Origin area narrows posteriorly from V1 to V3 |  |  |  |  |  |  |  |  |  |
| *Trilepida koppesi* | Present | Wide | Adjacent to prootic-supraoccipital-otooccipital suture descending over otooccipital | Wide dorsally at V1 | Narrow at V3 | N/A | N/A | Origin area narrows posteriorly from V1 to V3 |  |  |  |  |  |  |  |  |  |
| *Trilepida macrolepis* | Present | Wide | Adjacent to prootic-supraoccipital-otooccipital suture descending over otooccipital | Wide dorsally at V1 | Narrow at V3 | N/A | N/A | Origin area narrows posteriorly from V1 to V3 |  |  |  |  |  |  |  |  |  |
| *Trilepida salgueiroi* | Present | Wide | Adjacent to prootic-supraoccipital-otooccipital suture descending over otooccipital | Wide dorsally at V1 | Narrow at V3 | N/A | N/A | Origin area narrows posteriorly from V1 to V3 |  |  |  |  |  |  |  |  |  |
| ***Musculus ceratomandibularis*** | | | | | | | | |  |  |  |  |  |  |  |  |  |
| *Epictia ater* | Present | Posteroventral limit of the dental concha | Lateral unit at the level of 2^nd^ rib; Medial unit onto its opposite at the level of V6; middle unir onto the cornua and lingual process of hyoid | N/A | N/A |  |  | Three units present |  |  |  |  |  |  |  |  |  |
| *Epictia phenops* | Present | ? | ? | N/A | N/A |  |  | Three units present |  |  |  |  |  |  |  |  |  |
| *Epictia tenella* | Present | Posterior; posterior to anteroventral limit of dorsoposterior process of dentary | Lateral and medial units damaged; middle unit onto the cornua and also lingual process of hyoid | N/A | N/A |  |  |  |  |  |  |  |  |  |  |  |  |
| *Mitophis lepitepileptus* | Present | *Via* long tendon to the posteroventral limit of the dentary | Lateral unit at the level of the first rib; medial unit onto the lingual process of hyoid | N/A | N/A |  |  | Only lateral and middle units present; Tendon of origin originates ventrally to the *M. cervicumandibularis* tendon of insertion |  |  |  |  |  |  |  |  |  |
| *Rena dulcis* | Present | *Via* short tendon to the lateral face of dorsoposterior process of the dentary | Lateral unit at the level of 2^nd^ rib; Middle unit onto anterior limit of hyoid cornua; Medial unit onto its opposite at the level of posterior limit of quadrate | N/A | N/A |  |  |  |  |  |  |  |  |  |  |  |  |
| *Rena humilis* | Present | *Via* short tendon to the lateral face of dorsoposterior process of the dentary | Lateral unit damaged; Middle unit onto hyoid lingual process; Medial unit onto its opposite at the level of V3 | N/A | N/A |  |  |  |  |  |  |  |  |  |  |  |  |
| *Rena segrega* | Present | *Via* short tendon to the lateral face of dorsoposterior process of the dentary | Lateral and medial units damaged; Middle unit onto hyoid cornua | N/A | N/A |  |  |  |  |  |  |  |  |  |  |  |  |
| *Rena unguirostris* | Present | *Via* short tendon to the lateral face of dorsoposterior process of the dentary | ; Medial unit onto its opposite at the level of posterior limit of quadrate; Middle unit onto hyoid lingual process and cornua; | N/A | N/A |  |  | Only medial and middle units present |  |  |  |  |  |  |  |  |  |
| *Siagonodon cupinensis* | Present | *Via* long tendon to the lateral lamina of dental cocnha | Lateral unit at the level of the 1^st^ rib; Middle unit onto hyoid cornua; Medial unit onto its opposite at the level of V2 | N/A | N/A |  |  |  |  |  |  |  |  |  |  |  |  |
| *Tetracheilostoma bilineatum* | Present | *Via* tendon to the body of dentary | Lateral unit at the level of 1^st^ rib; Middle unit onto cornua; Medial unit onto its opposite at the level of V1 | N/A | N/A |  |  | Tendon of origin bifid and shared with the *M. cervicomandibularis* |  |  |  |  |  |  |  |  |  |
| *Trilepida brasiliensis* | Present | *Via* tendon to the dorsoposterior process of the dentary | Lateral unit at the level of V3; Middle unit damaged; Medial unit onto its opposite at the level of V1/V2 | N/A | N/A |  |  | Tendon of origin bifid and shared with the *M. cervicomandibularis* |  |  |  |  |  |  |  |  |  |
| *Trilepida dimidiata* | Present | *Via* tendon to the dorsoposterior process of the dentary | Lateral unit at the level of posterior limit of parabasisphenoid; Middle unit onto hyoid cornua and lingual process; Medial unit onto its opposite at the level of V6 | N/A | N/A |  |  | Tendon of origin bifid and shared with the *M. cervicomandibularis* |  |  |  |  |  |  |  |  |  |
| *Trilepida fuliginosa* | Present | *Via* tendon to the dorsoposterior process of the dentary | Lateral unit damaged; Middle unit onto hyoid cornua; Medial unit onto its opposite at the level of V1 to V2 | N/A | N/A |  |  | Tendon of origin bifid and shared with the *M. cervicomandibularis* |  |  |  |  |  |  |  |  |  |
| *Trilepida jani* | Present | ? | Lateral unit at the level of V3; Medial unit onto its opposite at the level of V4 | N/A | N/A |  |  | Lateral and medial units present |  |  |  |  |  |  |  |  |  |
| *Trilepida joshuai* | Present | *Via* tendon to the dorsoposterior process of the dentary | Lateral unit at the level of V7; Middle unit onto hyoid cornua and lingual process; Medial unit onto its opposite at the level of V5 | N/A | N/A |  |  | Tendon of origin bifid and shared with the *M. cervicomandibularis* |  |  |  |  |  |  |  |  |  |
| *Trilepida koppesi* | Present | *Via* tendon to the dorsoposterior process of the dentary | Lateral unit damaged; Middle unit onto hyoid lingual process; Medial unit onto its opposite at the level of V3 to V5 | N/A | N/A |  |  | Tendon of origin bifid and shared with the *M. cervicomandibularis* |  |  |  |  |  |  |  |  |  |
| *Trilepida macrolepis* | Present | *Via* tendon to the dorsoposterior process of the dentary | Lateral unit damaged; Middle unit onto hyoid lingual process; Medial unit onto its opposite at the level of V3 | N/A | N/A |  |  |  |  |  |  |  |  |  |  |  |  |
| *Trilepida salgueiroi* | Present | *Via* tendon to the dorsoposterior process of the dentary | Lateral unit damaged; Middle unit onto hyoid lingual process; Medial unit damaged | N/A | N/A |  |  |  |  |  |  |  |  |  |  |  |  |
